# A Highly Reactive Cysteine-Targeted Acrylophenone Chemical Probe That Enables Peptide/Protein Bioconjugation and Chemoproteomics Analysis

**DOI:** 10.1021/jacsau.5c00692

**Published:** 2025-11-28

**Authors:** Constantin M. Nuber, Anna V. Milton, Benedikt Nissl, Maria C. Isaza Alvarez, Benjamin R. G. Bissinger, Manjima B. Sathian, Cedric D. Pignot, Annsophie Haberhauer, Dongqing Wu, Céline Douat, Sabine Schneider, Stephan M. Hacker, Pavel Kielkowski, David B. Konrad

**Affiliations:** † Department of Pharmacy, 9183 Ludwig-Maximilians-Universität München, Butenandtstr. 5-13, 81377 Munich, Germany; ‡ Department of Chemistry, Ludwig-Maximilians-Universität München, Butenandtstr. 5-13, 81377 Munich, Germany; § Department of Molecular Physiology, Leiden Institute of Chemistry, Universiteit Leiden, Einsteinweg 55, 2333 CC Leiden, The Netherlands; ∥ Department of Pharmaceutical Sciences, Universität Wien, Josef-Holaubek-Platz 2, 1090 Vienna, Austria

**Keywords:** Cysteine-reactive chemotypes, Bioconjugation, Chemoproteomics, Chemical probe development, Reaction kinetics

## Abstract

To date, a variety of covalent cysteine-reactive chemical probes have been reported. The most common ones include maleimide for bioconjugation and iodoacetamide alkyne (IAA) for chemoproteomics analyses. Their applicability, however, is limited due to, e.g., the hydrolytic lability of maleimide adducts and the slow reaction kinetics as well as the inability to record the entirety of the cysteinome with IAA. This is compounded by the missing potential to fine-tune their reactivity tailored to advanced applications. To generate a high-reactivity, cysteine-selective chemical probe with broad utility, we have performed an in-depth investigation into the acrylophenone scaffold. The aryl group connected to the vinyl ketone chemotype can be readily substituted, which provides the potential to fine-tune the reactivity and install a bioorthogonal handle. We took advantage of this feature by modifying acrylophenone-alkyne (APA) with two ortho chlorine groups to generate *ortho*-dichloroacrylophenone-alkyne (CAPA), which increased the stability of the probe and the yield of its cysteine adducts. To showcase the reactivity, we performed reaction rate analyses with model reagents. The selectivity was demonstrated by specifically labeling cysteine residues within two peptides under physiological conditions. To investigate its utility toward bioconjugation reactions, we performed the stoichiometric labeling of two proteins. Remarkably, CAPA was successfully implemented as a high-reactivity cysteine-selective chemical probe into activity-based protein profiling (ABPP) experiments using both in-gel fluorescence (in-gel ABPP) and mass spectrometry analyses. The chemoproteomics workflow, named isoDTB-ABPP, allowed us to highlight that CAPA provides a complementary approach to IAA in expanding the coverage of the cysteinome.

## Graphical abstract

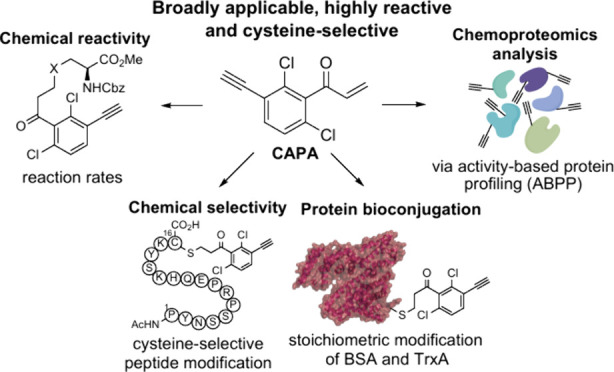

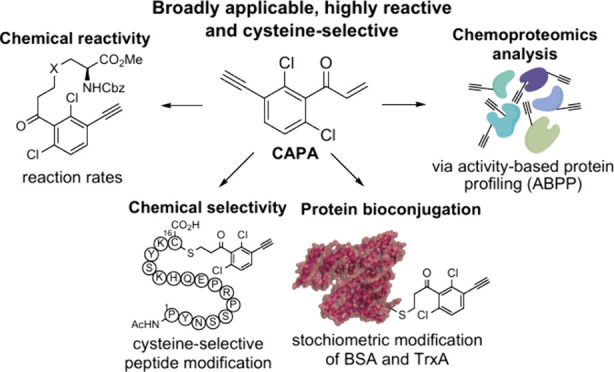

## Introduction

Chemical tools that enable amino acid-specific labeling have consolidated the understanding of the functions of their target sites and proteins over more than two decades.^1^ In particular, the bioconjugation of cysteine side chains is widely used.^2,3^ Despite its low abundance, cysteine is present in 97% of all human proteins,^4^ which makes it an ideal target. The chemical properties of cysteine, especially its high nucleophilicity, make it targetable with small electrophiles. Its high nucleophilicity originates from the large radius of the sulfur atom, which enables better stabilization of negative charges, compared to the smaller oxygen, e.g., which results in a p*K*_a_ of 8.2 or lower, depending on the local protein microenvironment.^5–7^ In many cases, this feature allows deprotonation under physiological conditions. These properties have imparted cysteine with a unique position among the proteinogenic amino acids. Functional groups (named “chemotypes” or “warheads”) that are able to selectively label cysteine residues, have led to the development of numerous chemical probes (Figure 1). The majority of cysteine-reactive probes that were developed over the past years are based on two different reaction pathways: (a) a nucleophilic substitution (S_N_), e.g., to iodoacetamide-alkyne (IAA, **1**)^8^ or (b) a Michael addition, e.g., to maleimides (**2**) (Figure 1).^9^

**Figure 1:**
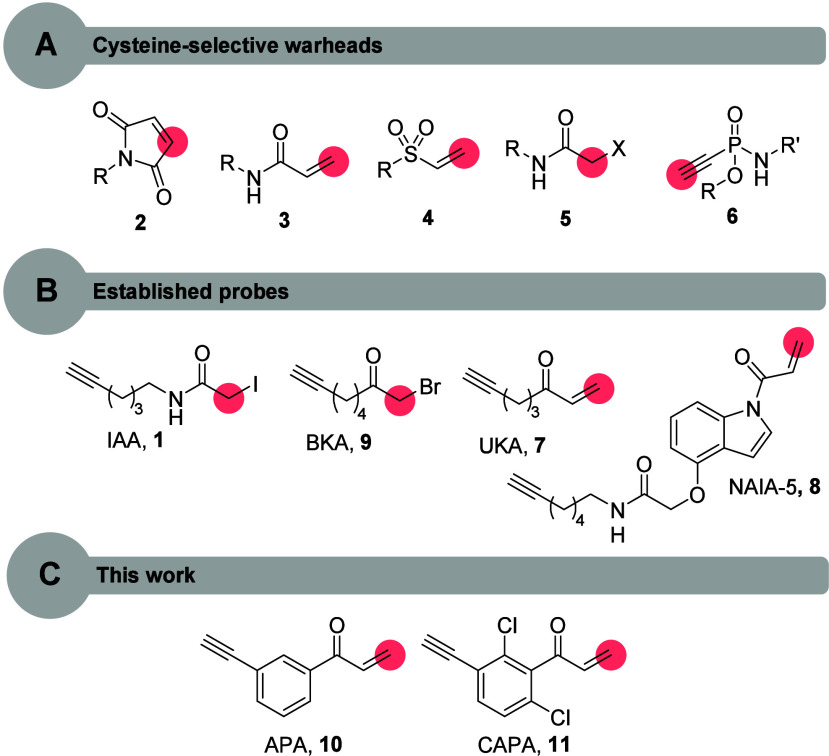
(A) Overview of selected literature-known cysteine-selective warheads. (B) Established high-reactivity cysteine-targeted probes. (C) Newly developed high-reactivity cysteine-selective probes (red: electrophilic position).

The low-reactivity warheads acrylamide (**3**),^10,11^ vinyl sulfone (**4**),^12,13^ and chloroacetamide (**5**),^14^ for example, can be used for the development of targeted covalent inhibitors (TCIs),^15,16^ because they require a ligand structure to bind to a protein binding pocket in proximity to a cysteine to facilitate a reaction. Moderate-reactivity chemotypes, such as maleimide (**2**)^17^ and phosphonamidate (**6**) that form stable covalent bonds have been employed for the generation of antibody–drug conjugates (ADCs).^18–20^ Remarkably, the high reactivity of iodoacetamide (**1**) and alkyl vinyl ketone (**7**) compounds^21^ was used to establish activity-based chemical probes (ABPs)^22^ that are used along with the chemoproteomics method activity-based protein profiling (ABPP) to pursue reactivity profiling^2^ or as competitors in ligandability profiling experiments.^23^ This powerful method has enabled the identification of ligand structures that access protein binding pockets on targets that were considered undruggable and paved the way for understanding the mode-of-action (MoA) of drugs as well as the function of proteins within the proteome.^24^ More recent examples^25^ for cysteine-reactive chemical probes that were reported include *N*-acryloylindole-alkynes (NAIAs),^26^ such as NAIA-5 (**8**), heteroaromatic sulfones,^27^ hypervalent iodine reagents^28^ and organogold derivatives.^29^ With the publication of the comprehensive repository for human cysteine chemoproteomics data named CysDB in 2023, however, only 24% of the cysteinome (62,888 residues) was recorded.^30,31^ We reason that the extension of the reach of chemoproteomics experiments can be facilitated through the identification and in-depth characterization of new cysteine-reactive chemotypes and chemical probes that label a fraction of residues complementary to the established tools. This extended reach is envisaged to enable, for example, the detection of cysteines that reside within binding pockets on proteins that were considered undruggable in the past, which forms the foundation for the discovery of ligand structures that engage these targets.

It is highly desirable that new chemical probes host the opportunity to modify their electronic properties, which may enable assessing changes in the coverage of residues and fine-tuning their reactivity depending on the application. At the same time, it would allow harnessing these tools in various applications, such as bioconjugation or the generation of ADCs. Due to the chemical structure of established probes, such as IAA (**1**), α-bromomethyl ketone-alkyne (BKA, **9**), and unsaturated ketone alkyne (UKA, **7**), fine-tuning their reactivity with electron-active functional groups is not possible without strongly impacting the steric properties of the molecule. While adding an *N*-benzyl group to iodoacetamide in place of the alkyl moiety might be possible without significantly impacting the size of the chemical probe,^32^ the substituents that are added to this benzylamide segment do not have an effect with the same significance to the chemotype reactivity as those added through the direct conjugation of an aryl group to a ketone.^33^ Although the reactivity of the NAIA scaffold (**8**) can be modulated with electron-active groups, this chemical probe class is susceptible to hydrolysis of the amide bond, which limits substitution to functionalities that do not enhance its lability.^26,34^

A challenge for the development of new high-reactivity chemical probes is to establish high selectivity for one particular amino acid side chain, which is compounded by the desired applicability on peptides, proteins, or even entire proteomes, which requires taking a deviation in p*K*_a_ values into account depending on local protein microenvironments. In addition, the synthetic chemical probes have to be suitable to use in aqueous media, at biocompatible temperatures (37 °C), and at near-neutral pH. They have to tolerate buffer salts and remain intact in sophisticated biological workflows. To ensure compatibility with mass spectrometry analysis, they need to be stable toward fragmentation or result in useful diagnostic ions. Finally, since peptides and proteins are often restricted to low concentrations, the covalent binding requires a high reaction rate and yield.^35^ Thus, the identification of appropriate chemical probes for targeting specific amino acids in proteins remains an ongoing quest in chemical biology.

## Results and Discussion

Herein, we perform the synthesis and in-depth characterization of the tunable aryl vinyl ketone functionality as a foundation for the development of cysteine-reactive chemical tools. This study led to the addition of a new type of highly reactive, amino-acid-selective compounds to the small molecule probe toolbox with broad applicability, including for bioconjugation and chemoproteomics analysis. The probe design enables modular modifications based on the desired reactivity. While only a limited number of modifications can be implemented to adjust the reactivity of the iodoacetamide chemotype, our scaffold allows, for example, the addition of electron-withdrawing or electron-donating groups to the aryl ring with a strong impact on the reactivity and stability. In addition, it allows the modification of the ketone group to mask the reactivity, which was demonstrated in the generation of a photolabile version of BKA (**9**), whereby the free chemical probe can be generated through a triggered release.^33,36^ The enhancement of probe stability was of particular interest to us, since the commonly used IAA (**1**) is sensitive to light.^37^ Our design for chemical probes is based on the incorporation of (1) a phenyl core as a linker that is amendable to electron active substitution to enable modulating the reactivity, (2) an α,β-unsaturated ketone chemotype, and (3) an alkyne handle in the *meta* position for bioorthogonal modifications.

The initially investigated chemical probe, namely, acrylophenone-alkyne (**10**, APA), was synthesized in 4 steps (for details see Supporting Information). For this compound, we observed decomposition during storage (−20 °C for >1 month) which was accompanied by a gradual color change to yellow and the formation of a precipitate. A clean sample could be obtained from this mixture through a trituration with MeCN (see Supporting Information for details). Since acrylophenone derivatives are commonly employed to generate polymers, we hypothesize that the decomposition is the result of polymerization.^38–40^

To stabilize the compound, we harnessed the adaptability of our probe design and installed two chlorine atoms *ortho* to the ketone functionality. This modification was envisaged to increase the steric bulk and, thereby, shield the two proximal carbon atoms within the ketone and the alkene units from reactive interactions akin to the principal of the Yamaguchi reagent.^41^ We reasoned that this substitution additionally directs nucleophilic attacks to the terminal alkene carbon.

The computed structure of APA (**10**) is characterized by a planar, fully conjugated structure, while the steric bulk of the chlorine atoms of *ortho*-dichloroacrylophenone-alkyne (CAPA, **11**) forces the vinyl ketone structure to twist out of the plane and, thereby, adopt an orthogonal position to the aryl π-system (see force field calculations, Figure 2). CAPA (**11**) was synthesized in 5 steps (for details see Supporting Information) and found to be particularly bench-stable, whereby stock solutions (20 mM in MeCN) can be stored at −20 °C for more than 6 months with multiple freeze–thaw cycles without a change in the concentration.

**Figure 2:**
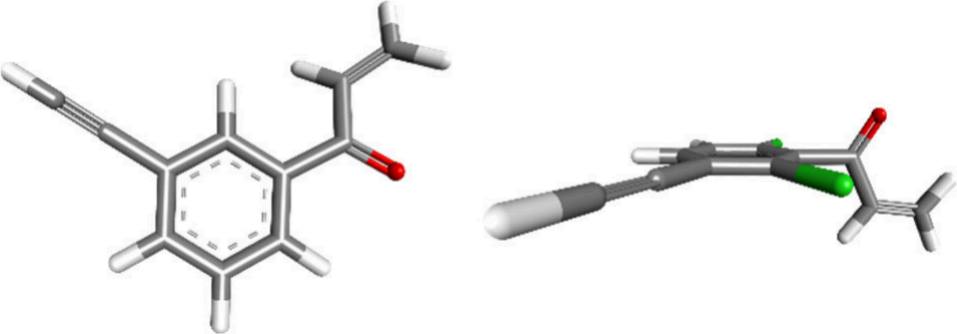
Minimized energy 3D structures of acrylophenone-alkyne (APA, **10**) (left) and *ortho*-dichloroacrylophenone-alkyne (CAPA, **11**) (right). Chlorine atoms are highlighted in green (Maestro, Forcefield OPLS4, Meth. PRCG, 22 iterations, convergence threshold = 0.05 kJ mol^–1^).

With the chemical probes in hand, we characterized their reactivity toward the most nucleophilic amino acids using the model systems *N*-Cbz-l-Cys-OMe (**S1**), *N*-Cbz-l-Lys-OMe (**S3**) and *N*-Cbz-l-Ser-OMe (**S21**). To enable monitoring the reaction rate at a time scale compatible with high performance liquid chromatography (HPLC) analysis, the experiments were performed at 0 °C (for details, see Supporting Information). Remarkably, compared to the commonly used IAA (**1**), APA (**10**) and CAPA (**11**) show a significantly higher reactivity toward cysteine thiols (**S1**) in phosphate-buffered saline (PBS) pH 7.4 systems, forming Cys-IAA (**12**), Cys-APA (**13**), and Cys-CAPA (**14**) adducts, respectively (Figure 3A). While IAA (**1**) is reacting with second-order rate constants of *k*_2_(IAA) = 0.0720 L/(mol·s) with *N*-Cbz-l-Cys-OMe (**S1**) in an S_N_2 reaction (Figure 3B), both APA (**10**) and CAPA (**11**) show sulfa-Michael additions with fast, exponential rates: *k*_2_(APA) = 13.2 L/(mol·s) (Figure 3C), *k*_2_(CAPA) = 5.82 L/(mol·s) (Figure 3D). This results in 25% average product formation for IAA (**1**) in 3 h while APA (**10**) and CAPA (**11**) yield 71% and 83%, respectively. It is worth noting that, at room temperature, CAPA (**11**) binds the cysteine model compound in 94% yield after 10 min and >99% in 20 min (for details see Supporting Information Section 3.3, Table S1). Overall, APA (**10**) has the highest reaction rate, while CAPA (**11**) provides the highest yields. While the chlorine atoms are mildly electron-withdrawing in nature, we reason that these *ortho*-substituents, which force the vinyl ketone out of the planar arrangement, limit its conjugation to the π-system, which decreases the reactivity. The decreased level of conjugation to the aryl ring can be observed through a downfield shift of the carbonyl signal in the ^13^C NMR.^42^ To assess potential off-target reactivity, we monitored the reaction of the electrophiles APA (**10**) and CAPA (**11**) with the most common nucleophilic off-target amino acids using *N*-Cbz-l-ys-OMe (**S3**) and *N*-Cbz-l-Ser-OMe (**S21**) as model systems. We found that the probes remain largely unaffected by lysine and serine, even at rt, allowing the recovery of 84–99% after 3 h (for details see Supporting Information, Section 3.5, Figures S2 and S4). Since pH values as well as the p*K*_a_ of cysteine thiols in certain protein microenvironments can deviate from those that are exposed to PBS on the protein surface,^44^ we next determined the pH-dependent reactivity of APA (**10**) and CAPA (**11**). We found that for CAPA (**11**), a higher pH (*k*_2_(CAPA, pH 8.2) = 29.7 L/(mol·s)) leads to enhanced reactivity, while a lower pH is associated with a decreased reaction rate in PBS (*k*_2_(CAPA, pH 6.6) = 2.45 L/(mol·s)) at 0 °C (Figure 3E,F). In addition, concomitant changes in the average yields after 3 h were observed, providing Cys-CAPA (**14**) in 76% (pH 6.6) and 91% (pH 8.2), respectively. These results are in line with other examples of sulfa-Michael additions.^45^ For APA (**10**), similar trends were observed: A lower pH leads to a decreased reaction rate (*k*_2_(APA, pH 6.6) = 4.12 L/(mol·s)) at 0 °C along with a lower average yield for the formation of Cys-APA (**13**) of 44% after 3 h (Figure S4A). While leading to a faster consumption of APA (**10**), an increase to pH 8.2 is associated with significantly lower yields, providing only 17% of **13** after 3 h (Figure S4B), which hindered the generation of meaningful second-order rate constants due to the decomposition of the chemical probe.

**Figure 3:**
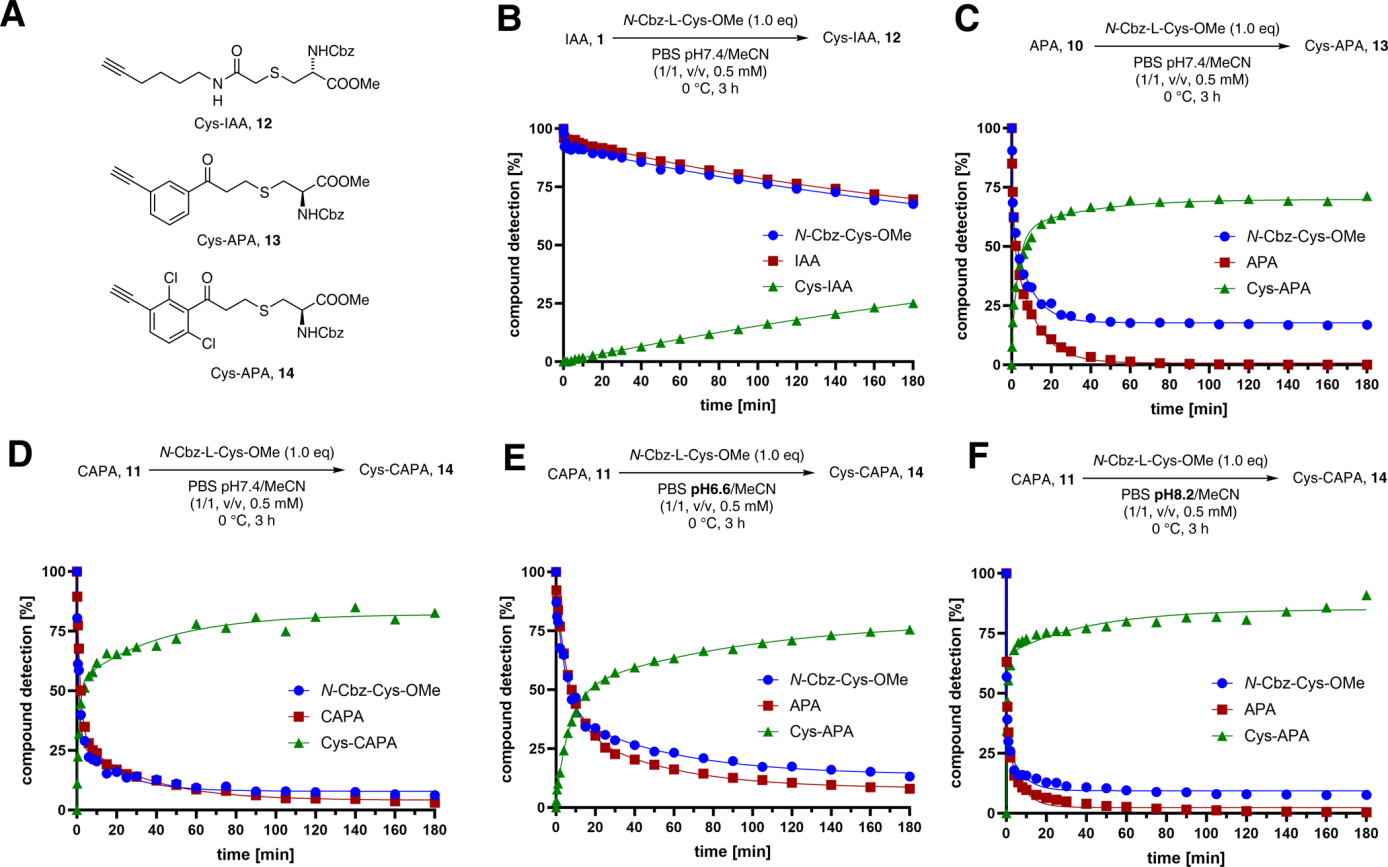
(A) Chemical structure of Cys-IAA (**12**), Cys-APA (**13**), and Cys-CAPA (**14**). (B, C, D) Time-dependent monitoring of the absolute amount of (B) IAA (**1**), (C) APA (**10**), or (**D**) CAPA (**11**) in a reaction with N-Cbz-l-Cys-OMe (**S1**) and the corresponding products **12**, **13**, and **14** in PBS at pH 7.4 at 0 °C. (E, F) Monitoring of the absolute amount of CAPA (**11**) and N-Cbz-l-Cys-OMe (**S1**) and yield of the Michael adduct during the reaction at 0 °C in PBS at (E) pH 6.6 and (F) pH 8.2; (B–G) All data was normalized; 4-nitrobenzonitrile was used as an internal standard; aliquots were taken at specific time points and quenched with H_2_O/MeCN (1/1) containing 0.1% of TFA.; *n* = 3.

These results highlight the strong potential of CAPA (**11**) as high-reactivity cysteine-selective chemical probe and provide further evidence that APA (**10**) is unstable under certain conditions. Based on these factors, we have chosen to pursue an in-depth characterization with regard to bioconjugation and ABPP chemoproteomics experiments of CAPA (**11**), while including a comparison to APA (**10**) for their reactivity with chemical model systems and gel-based ABPP (in-gel ABPP) experiments. To uncover the extent of its utility, we assessed the application of CAPA (**11**) in peptide as well as protein bioconjugation and chemoproteomics experiments that harness an ABPP workflow, which relies on an effective and selective binding to cysteine thiol residues in, e.g., cell lysates.

To investigate whether CAPA (**11**) is compatible with copper­(I)-catalyzed azide–alkyne cycloaddition (CuAAC) reactions, which are a prerequisite for many bioconjugation or chemoproteomics workflows, we synthesized the cysteine-CAPA-desthiobiotin (DTB) adduct (DTB-Cys-CAPA, **15**) via coupling of **14** to desthiobiotin-PEG_3_-azide (DTB-PEG_3_-N_3_, **16**), using standard “click” chemistry reaction conditions (Scheme 1). This experiment allowed us to deduce that the substitution pattern of the acrylophenone alkyne-based probe (**11**) was compatible with the reaction and allowed us to assess whether the installation of a triazole retained a stable covalent carbon–sulfur bond within the CAPA–cysteine Michael adduct. These features are necessary to enrich the labeled peptides or proteins via the DTB purification tag for follow-up experiments.^46^

**Scheme 1:**
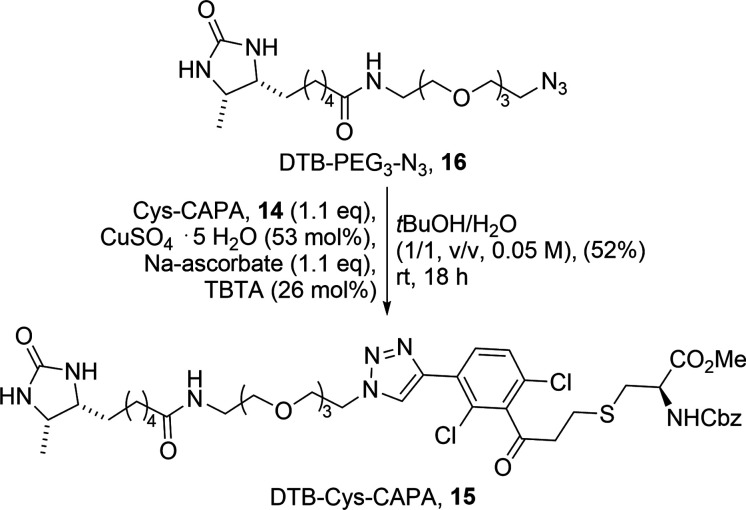
Copper­(I)-Catalyzed Azide–Alkyne Cycloaddition (CuAAC) Reaction of DTB-PEG_3_-N_3_ (**16**) and Cys-CAPA (**14**) to Give Cys-CAPA-DTB (**15**)

The reversibility of the probe–thiol conjugates, prior to and after the CuAAC reaction, was examined in different buffer systems to assess pH-dependent stability as well as stability toward the biological reducing agent glutathione (GSH), which harbors a thiol group and is present at high concentrations (up to 10 mM) in cells.^47^ In acidic (pH 4.0), neutral (pH 7.4) or basic (pH 9.2) environments, the adducts were recovered in >99%, 97%, 78% (for **13**), >99%, 91%, 67% (for **14**), and >99%, 90%, 72% (for **15**) over 24 h at rt (for details see Supporting Information, Section 3.4, Figure S3). GSH was used to test the reversibility of the covalent adduct by analyzing the exchange of *N*-Cbz-l-Cys-OMe (**S1**) for this natural reducing agent with **10**, **11**, and **15** under physiological conditions (PBS buffer pH 7.4 at 37 °C). After 6 h, the probe–thiol conjugates were recovered at 87% (for CAPA, **11**) and >99% (for APA, **10**). The Cys-CAPA-DTB (**15**) could be recovered at 94%, 89%, and 70% after 3, 6, and 24 h under the same conditions, highlighting that the formation of the triazole does not impact the stability of the Cys-CAPA (**14**) adduct. It is worth noting that our reversibility tests were performed at 37 °C, while most biological workflows are done at room temperature or on ice after probe treatment, which decreases the rate of decomposition.^48^

Verification of cysteine selectivity in the presence of multiple amino acids was examined by modifying two peptides (laminin (925–933), a 9-mer with the sequence CDPGYIGSR, and nitric oxide synthase (NOS) blocking peptide (599–613), a 16-mer with the sequence Ac-PYNSSPRPEQHKSYKC). The modification was performed by adding 1.0 equiv of CAPA (**11**) to a solution of the peptide in 1/1 PBS pH 7.4/MeCN at a 100 μM concentration and stirring for 1 h at room temperature. Full conversion was reached even at 15–20 mg scales and the modified peptides could be isolated by preparative HPLC in 42% (**17**) and 69% (**18**) yield (Scheme 2, see Supporting Information for experimental details). The N-terminal cysteine is flanked by aspartic acid within laminin (925–933), while the C-terminal cysteine is flanked by lysine in the NOS blocking peptide (599–613). This feature provided the opportunity to use an Asp-N endoproteinase and a trypsin digest, respectively, to cleave the labeled cysteine residues off the respective peptides and unambiguously validate the bound residue by mass spectrometry (MS) analysis. Performing this experiment demonstrated the chemoselectivity of the probe in complex settings and in the presence of a variety of nucleophilic amino acids, including arginine, asparagine, glutamic acid, glutamine, histidine, lysine, serine and tyrosine (for details see Supporting Information, Section 4, Figures S5 and S7).

**Scheme 2:**
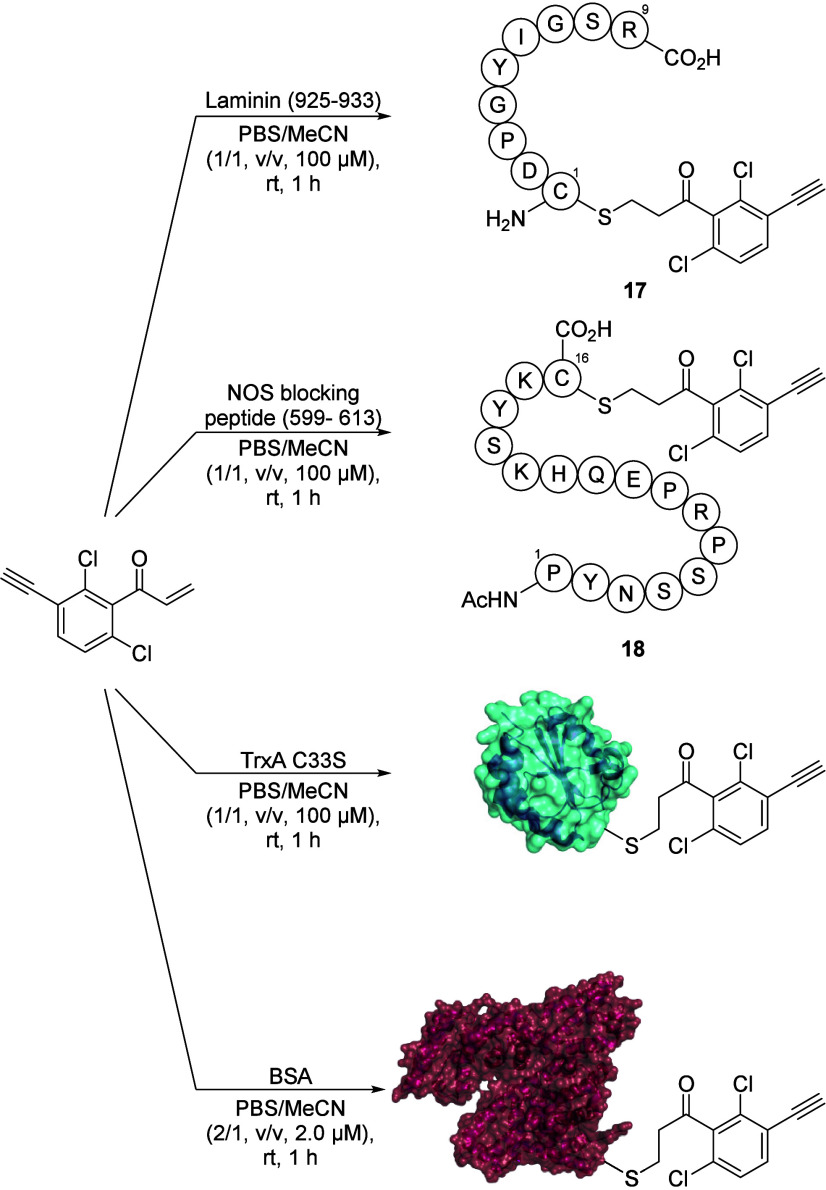
Bioconjugation of the Peptides Laminin (925–933) and NOS Blocking Peptide (599–613) and the Proteins TrxA C33S as well as BSA with CAPA (**11**)­^a^

a Protein models: PDB 6H1Y (TrxA) and 4F5S (BSA).

Next, the modification of whole proteins was performed with stoichiometric amounts of CAPA (**11**) using (a) the thioredoxin-1 (TrxA) C33S mutant, which contains one cysteine,^49^ dissolved in 1/1 PBS pH 7.4/MeCN at a 100 μM concentration and stirred for 1 h, and (b) bovine serum albumin (BSA), wherein the wild-type protein contains a single reduced cysteine that is not engaged in a disulfide bond, dissolved in 2/1 PBS pH 7.4/MeCN at a 2 μM concentration and stirred for 1 h. We observed efficient modification through an analysis of the probe–protein complex formation by MS. The highest intensity mass was found to be 13.18667 kDa [M + H]^+^ for TrxA C33S and 1588.106 Da [M + 42H]^42+^ for BSA (for details see Supporting Information, Section 5, Figures S8–S12).

The next step within our in-depth characterization of the cysteine-targeted chemical probes entailed assessing the concentration-dependent labeling of protein lysates with APA (**10**) and CAPA (**11**) compared to IAA (**1**) using in-gel ABPP experiments.^36^ This experiment was performed by treating PC9 cell lysates with the chemical probes for 1 h followed by coupling to tetramethylrhodamine (TAMRA) azide (**S25**) via CuAAC and visualizing the labeled proteins on an SDS-PAGE gel via fluorescence imaging. Figure 4A highlights that at equal concentrations the highest signal intensity was obtained with APA (**10**) followed by IAA (**1**) and CAPA (**11**). The stability of protein adducts of the probes (**1**, **10**, and **11**) in protein lysates was determined using an adapted in-gel ABPP workflow. In short, PC9 cell lysates were treated with the corresponding probes for 1 h at a 10 μM concentration and subjected to CuAAC with TAMRA azide. The reaction was quenched with Laemmli buffer followed by an incubation period ranging from 0–24 h at room temperature before running the SDS-PAGE. We found that the probe–protein complexes for all three tested compounds (**1**, **10**, and **11**) remain stable throughout all time points (Figure 4C). This experiment complements the stability analysis via HPLC by demonstrating the suitability of the chemical probes to working with complex samples in an ABPP workflow. Remarkably, comparing the in-gel ABPP results from APA (**10**) and CAPA (**11**) with IAA (**1**) demonstrates that both new compounds have strong overlaps in the engaged bands with the established probe (Figure 4A,C, examples highlighted by a blue asterisk), which are complemented by a unique set of new fluorescent signals (examples highlighted by a red asterisk). For both experiments, a Coomassie stain was performed to confirm equal loading of the proteins (Figure 4B,D)

**Figure 4:**
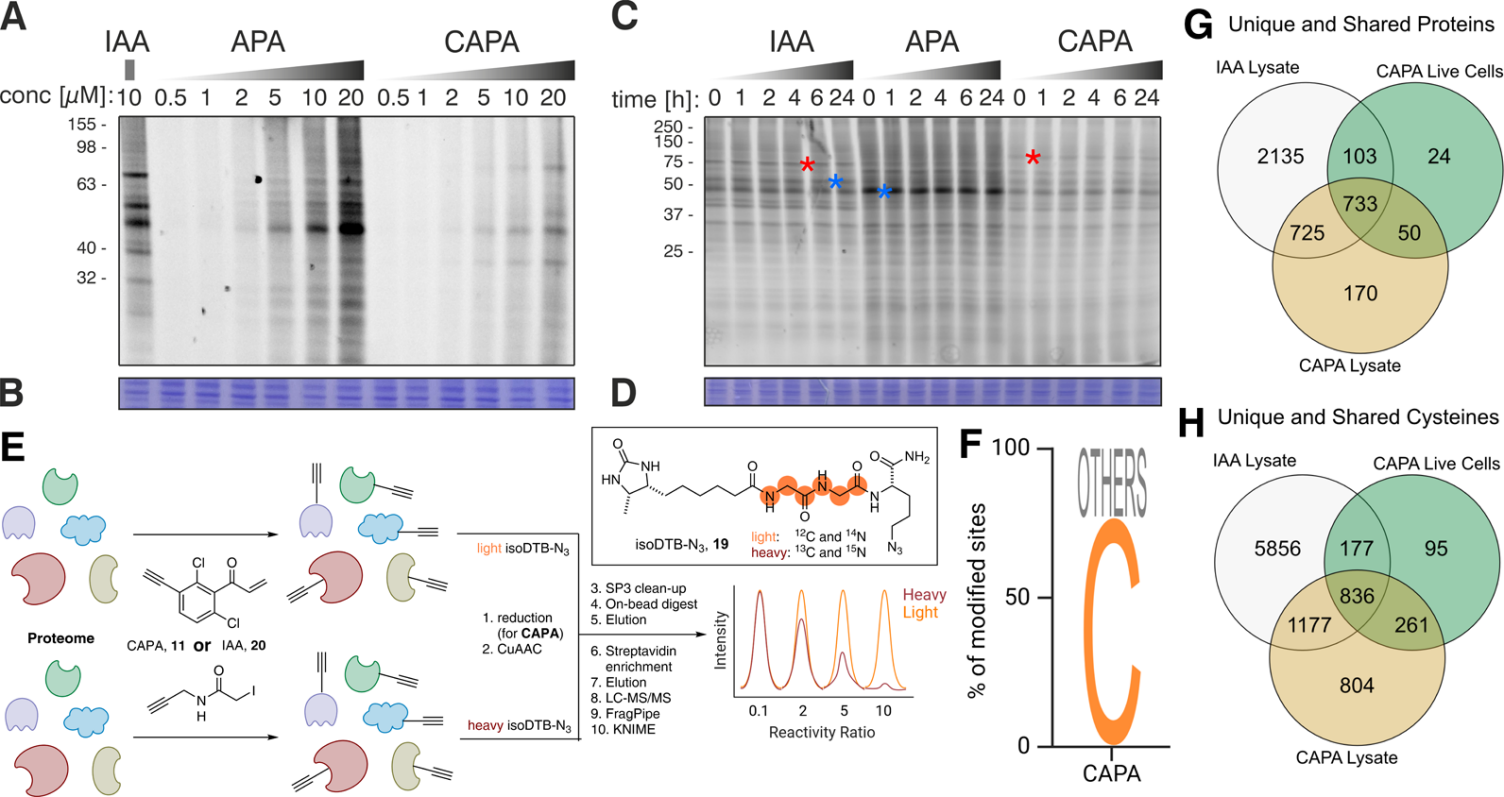
(A) Concentration-dependent labeling of protein lysates with APA (**10**) and CAPA (**11**) compared to IAA (**1**). The soluble fraction of PC9 cell lysates was treated for 1 h with the corresponding probe and subsequently subjected to a CuAAC with TAMRA azide. The reaction was quenched with Laemmli buffer after 1 h and analyzed by combining SDS-PAGE with fluorescence imaging. (B) Total protein was stained with Coomassie to visualize equal loading and the absence of protein degradation. (C) Assessing the stability of APA (**10**) and CAPA (**11**) adducts in protein lysate compared to IAA (**1**) to determine the stability in combination with complex samples and an ABPP workflow. The soluble fraction of PC9 cell lysates was treated for 1 h with 10 μM of the corresponding probe and subjected to CuAAC with TAMRA azide. The reaction was quenched with Laemmli buffer after 1 h followed by an incubation period for the indicated time. The analysis was performed by combining SDS-PAGE with fluorescence imaging. (D) Total protein was stained with Coomassie to visualize equal loading and absence of protein degradation. (E) IsoDTB-APBB workflow to investigate the unbiased chemoselective labeling as well as protein coverage at 200 μM probe concentration using the chemoinformatics tools Fragpipe and KNIME for data analysis. (F) Amino acid selectivity of CAPA (**11**) in HeLa cell lysate subjected to isoDTB-ABPP. (G) Comparison of unique proteins identified by IAA (**20**) in HeLa cell lysate and CAPA (**11**) in HeLa cell lysate and in live HeLa cells subjected to isoDTB-ABPP. (H) Comparison of unique cysteines identified by IAA (**20**) in HeLa cell lysate and CAPA (**11**) in HeLa cell lysate and in live HeLa cells subjected to isoDTB-ABPP.

Prior studies have described the need to perform a reduction of the ketone within vinyl ketone chemotypes, such as BKA (**9**), to the corresponding alcohols after protein labeling to enable mass spectrometry-based chemoproteomics experiments.^33,36,50,51^ An optimization of the reduction of Cys-CAPA (**14**) in PBS resulted in the identification of conditions that harnessed a pH of 8.2 and a 330 mM final concentration of NaBH_4_ at 0 °C for 30 min and room temperature for 15 min. The conditions were implemented in a chemoproteomic workflow (for details, see Supporting Information, Section 7), which was combined with an adjustment of the pH prior to and after the reduction step to enable transitioning back to the protocol.

Based on our preliminary investigations into the performance of a CuAAC reaction with CAPA (**11**), the pH-dependent stability of its cysteine adducts (**13** and **14**) and a reduction protocol, we explored the potential of using CAPA (**11**) as a cysteine-reactive probe in an adapted version of the ABPP workflow that relied on the isotopically labeled DTB tags (isoDTB-ABPP).^52^ We used a general and unified isoDTB-ABPP workflow that was recently established and aims to democratize chemoproteomics analysis by building a foundation for nonspecialized groups to harness this powerful analytical method (Figure 4E). This workflow relies on commercially available isoDTB tags to enable the enrichment of the CAPA-bound proteins and to differentiate two differently labeled proteomes as part of MS analysis. A detailed description of the workflow is provided in the Supporting Information. Briefly, 2 × 100 μL of HeLa cell lysate (2 mg/mL in PBS) was treated with 200 μM IAA (**20**) or CAPA (**11**) and incubated for 1 h. For the CAPA-bound lysate, a reduction step was performed based on the optimized conditions described above. This included adjusting the pH of the lysate to 8.2 through addition of aqueous NaOH (1.6 μL, 0.1 M) followed by a reduction with an aqueous solution of NaBH_4_ (20 μL, 2 M). The reaction was quenched with acetic acid (3.0 μL), and the samples were neutralized with aqueous NaOH (28 μL, 0.1 M). Next, a CuAAC with heavy or light isoDTB azide (**19**) was performed for 1 h. The light- and heavy-treated isoDTB samples were combined and subjected to SP3 cleanup followed by on-bead digestion using trypsin. After elution from the SP3 beads, the isoDTB-bound peptides were enriched with high-capacity streptavidin beads, eluted, and concentrated via SpeedVac. The peptide pellet was resuspended in 30 μL of H_2_O containing 0.1% of TFA, and 5 μL were analyzed by LC-MS/MS. With this method, the amino acid selectivity, the cysteine coverage, and the overlap to IAA (**20**) was explored.

Identification and quantification of heavy and light isoDTB-bound peptides was performed with Fragpipe.^53,54^ To investigate the amino acid selectivity, the offset search function in MSFragger was used,^55^ combined with downstream processing of the data using KNIME. Using this approach, unambiguously annotated engagement sites were assessed which determined that CAPA-bound sites corresponded to cysteines in 76% (Figure 4F), which denotes the chemoselectivity of the chemical probe on a proteome-wide scale. After confirming the selectivity of CAPA (**11**) toward cysteine, a closed search was performed according to our protocol. Here, we identified 3078 CAPA-modified cysteines on 1678 proteins, whereby 1065 sites were unique to the new chemical probe (CAPA, **11**) and 2013 cysteines overlapped with the labeling profile of IAA (**20**) (Figure 4G,H). To assess the utility of CAPA, we moved from using the chemical probe in cell lysate to live HeLa cells, which allowed profiling of 1369 modified cysteines on 910 proteins. Remarkably, within our study, 390 cysteines were labeled that were not annotated in the CysDB, where 75 were found using CAPA which showcased that the use of new cysteine-reactive probes holds the opportunity to expand the reach of conventional probes.^30,31^ Together with the in-gel ABPP experiment, this result highlights that CAPA (**11**) can be used to complement IAA (**20**) in order to increase the coverage of the detected cysteinome. Our results further demonstrate the utility of the unified isoDTB-ABPP workflow by showcasing the ability to adapt it to the use of new chemical probes.

## Conclusion and Outlook

In conclusion, we investigated the utility of the acrylophenone scaffold as a basis for the development of new high-reactivity chemical probes that enable the selective modification of cysteine residues. The study was conducted with APA (**10**) and CAPA (**11**). We examined their reactivity using reaction kinetics measurements with a chemical model system and the stability of the covalent adducts at different pH ranges as well as toward the thiol-based reducing agent GSH. While APA (**10**) is not stable for prolonged periods of time, the freshly synthesized compound could be used in these investigations and be employed in cell lysates to perform an in-gel ABPP experiment. CAPA (**11**) was subjected to an in-depth study to showcase its cysteine selectivity and suitability in labeling reactions with peptides, bioconjugation experiments with proteins, and chemoproteomics experiments. To employ CAPA (**11**) in chemoproteomics analyses, we have adapted a general, unified isoDTB-ABPP workflow which showcases the utility of this method and its ability to study new chemical probes. Remarkably, CAPA (**11**) showed an overlap of 2013 labeled cysteines with the commonly used IAA (**20**) and, at the same time, enabled labeling of 1065 unique cysteines. Comparing our results to the CysDB, 390 cysteines were found that were not previously annotated, 75 of which were labeled by CAPA, which aids in expanding the coverage of the detected cysteinome.

Due to the in-depth investigation that we have performed with CAPA (**11**), we reason that our approach could serve as a “blueprint” for the stepwise characterization of new covalent chemical probes to assess their reactivity level, amino acid selectivity profile, and utility for labeling reactions with peptides, bioconjugation experiments with proteins, and chemoproteomics experiments.

It is worth noting that our results establish vinyl ketones as high-reactivity cysteine-targeted warheads. The literature hosts several examples that describe the identification of covalent inhibitors for certain proteins, which contain derivatives of this chemotype, such as withangulatin A (WA).^56–58^ Based on our data, it is unlikely that the reactivity of vinyl ketones allows protein-specific covalent labeling, but rather leads to the engagement of multiple proteins by the chemical probes. This is exemplified by separate reports, each describing different protein targets to WA including as an inhibitor to PRDX6,^58^ COX-2,^56^ and TrxR1.^57^

Compared with IAA (**1** and **20**), CAPA (**11**) holds the potential to mask the reactivity of its chemotype, which can, in turn, be released through a defined trigger. This principle was demonstrated for BKA (**9**, Figure 1), which was used as a foundation to generate a photoactivatable chemical probe by transforming the ketone group into an acetal which could be removed with light.^33,36^ On this basis, we are in the process of investigating the ability to mask the vinyl ketone group of CAPA (**11**) and, thereby, enable the extension of the repertoire of responsive elements to release the chemical probe. This approach will allow us to study biological processes that rely on the selected triggers on a system-wide level in live cells.

## Supplementary Material





## Notes

Experimental procedures, reaction rate measurement data, additional plots, and spectra (PDF)
KNIME Analysis (XLSX)
