# Supplementary material for: A Highly Reactive Cysteine-Targeted Acrylophenone Chemical Probe That Enables Peptide/Protein Bioconjugation and Chemoproteomics Analysis
Source: JACS Au. 2025 Nov 28;5(12):5908–16. doi: 10.1021/jacsau.5c00692 (PMC12728623; doi:10.1021/jacsau.5c00692)
Supplement: Supplementary file 1 [file au5c00692_si_001.pdf]

A Highly Reactive Cysteine-targeted Acrylophenone Chemical Probe that Enables Peptide/Protein Bioconjugation and Chemoproteomics Analysis

Constantin M. Nuber[1,#], Anna V. Milton[1,#], Benedikt Nissl[1,#], Maria C. Isaza Alvarez[1], Benjamin R. G. Bissinger[1], Manjima B. Sathian[1], Cedric D. Pignot[1], Annsophie Haberhauer[1], Dongqing Wu[2], Céline Douat[1], Sabine Schneider[2], Stephan M. Hacker[3], Pavel Kielkowski[2], and David B. Konrad*[1,4]

[1]Department of Pharmacy, Ludwig-Maximilians-Universität München, Butenandtstr. 5-13, 81377 Munich, Germany

[2]Department of Chemistry, Ludwig-Maximilians-Universität München, Butenandtstr. 5-13, 81377 Munich, Germany

[3]Department of Molecular Physiology, Leiden Institute of Chemistry, Universiteit Leiden, Einsteinweg 55, 2333 CC Leiden, The Netherlands

[4]Department of Pharmaceutical Sciences, Universität Wien, Josef-Holaubek-Platz 2, 1090 Vienna, Austria

david.benjamin.konrad@univie.ac.at

Supporting Information

1. General Considerations 3

2. Chemical Procedures 4

2.1 Amino Acids 4

2.2 IAA 5

2.3 APA 7

2.4 CAPA 9

2.5 Cys-CAPA-DTB 12

3. Kinetic Measurements 15

3.1 General Procedure for Kinetic Measurements of Sulfa-Michael Additions 15

3.2 General Procedure for Stability Experiments of Sulfa-Michael Adducts 15

3.3 General Procedure for Determining Second-Order Rate Constants 15

3.4 Kinetic Measurements of Sulfa-Michael Additions, Plotted Data 16

3.5 Kinetic Measurements of Sulfa-Michael Additions, Raw Data 19

4. Peptide Modifications and Digests 24

5. Protein Modification 27

6. Protein Expression 32

7. Chemoproteomics Experiments 33

7.1 Cell culture 33

7.2 Proteome Preparation 33

7.3 In-gel ABPP Experiments 33

7.4 ABPP Coupled to LC-MS/MS 33

7.5 LC-MS/MS Measurement and Data processing 35

8. NMR Spectra 38

9. References 62

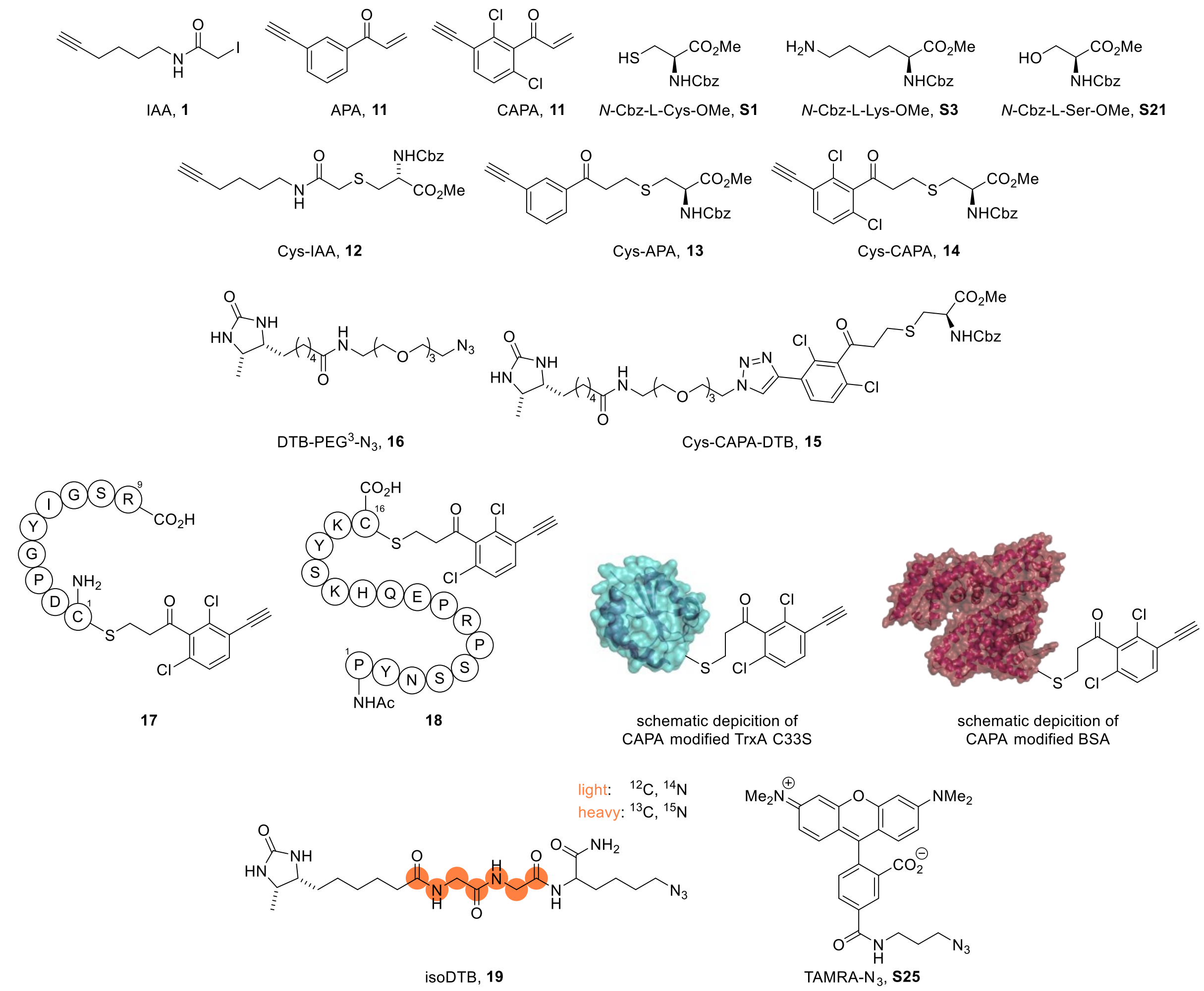

Figure S1: Selection of frequently used compounds in this work.

1. General Considerations

Unless otherwise stated: Commercially available starting materials were used without further purification. All reactions were carried out under a nitrogen atmosphere in flame-dried glassware. Syringes, which were used to transfer anhydrous organic phases or reagents, were purged with nitrogen prior to use. Dry organic phases, such as acetone, DCM, DMF, Et_2O, MeCN, THF and toluene were purchased from Thermo Scientific (≥99.9%) extra dry over molecular sieve, AcroSeal. Acetone, MeCN and toluene was purchased in HPLC gradient grade (≥99.9%) from Fisher Scientific. Water was purified with a Sartorius Arium water purification system. Aqueous (aq.) salt solutions were saturated (sat.). Dulbecco's phosphate buffered saline (PBS) solution and sodium hydrogen phosphate buffer solution pH9.2 were purchased from Sigma-Aldrich. Citric acid buffer solution pH4.0 was purchased from PanReac AppliChem. Raney-Ni was purchased from Sigma-Aldrich (W.R. Grace and Co. Raney 2800, slurry, in H_2O, active catalyst, product number: 221678). Chromatography purifications were performed using silica gel (SiO_2, 60 M, 0.040-0.063 mm) from Macherey-Nagel. Automated chromatography purifications were performed using an Advion Interchim puriFlash XS520Plus with normal phase (NP) PF-15SIHP-F0025 or reverse phase (RP) PF-15C18AQ-F0025 columns. The spots were visualized under UV (254 nm and 366 nm) and/or by staining the TLC plate with $KMnO_4$ solution (K_2CO_3, 10 g; $KMnO_4$, 1.5 g; H_2O, 150 mL; NaOH 10% in H_2O, 1.25 mL), *p*-anisaldehyde solution (conc. H_2SO_4, 10 mL; EtOH, 200 mL; AcOH, 3 mL; *p*-anisaldehyde, 4 mL), ninhydrin (0.3 g ninhydrin; *n*-BuOH, 100 mL; conc. AcOH, 3 mL) or vanillin (vanillin, 15 g; EtOH, 250 mL, conc. H_2SO_4, 2.5 mL). Yields refer to isolated yields of compounds estimated to be >95% pure as determined by ^1H NMR and/or HPLC analysis. The ^1H and ^{13}C spectra were recorded on Bruker Ascend 400 (400 MHz and 101 MHz for ^1H and ^{13}C, respectively). NMR-spectra were measured at 298 K and were analyzed with the program

MestreNova. Chemical shifts are reported as δ values in ppm relative to the residual organic phase peak (^{1}H NMR, ^{13}C NMR) in deuterated chloroform ($CDCl_3$: δ 7.26 ppm for ^{1}H NMR and δ 77.16 ppm for ^{13}C NMR), deuterated methanol (CD_3OD: δ 2.31 ppm for ^{1}H NMR and δ 49.00 ppm for ^{13}C NMR), deuterated DMSO ($(CD_3)_2SO$: δ 2.50 ppm for ^{1}H NMR and δ 39.52 ppm for ^{13}C NMR) or deuterated water (D_2O: δ 4.79 ppm for ^{1}H NMR). Abbreviations for signal coupling are as follows: s (singlet), d (doublet), t (triplet), q (quartet), quint (quintet), m (multiplet) and br (broad). Analytical and semi-preparative high performance liquid chromatography (HPLC) was performed on a Thermo Scientific Dionex UltiMate 3000-Serie equipped with a Diode Array Detector (DAD). Column eluent was monitored by UV detection at 214, 254, 280 and 300 nm and a flow rate of 1.0 mL × min^{-1} at 50 °C (for analytical) or 5.0 mL × min^{-1} at 25 °C (for semi-preparative) was applied. Purifications on an analytical scale were performed on a Nucleodur C18 Gravity column (4 × 100 mm, 5 µm, from Macherey-Nagel) and purifications on a semi-preparative scale were performed on a Nucleodur C18 Gravity column (10 × 250 mm, 5 µm, from Macherey-Nagel) using an automated fraction collector system from ThermoFisher Scientific. Analytical high performance liquid chromatography coupled with mass spectrometry (LC-MS) was performed on a rapid separation (RS) Thermo Scientific dionex ultimate 3000 series equipped with a Bruker microTOF II. For liquid chromatography high resolution mass spectrometry (LC-HRMS) analysis, the mass spectrometer (MS) was calibrated with ESI-L Low Concentration Tuning Mix from Agilent Technologies prior to a measurements and the spectra were referenced accordingly. Low resolution (LR) or HRMS was recorded on Finnigan MAT 95Q, Finnigan MAT 90 or JEOL JMS-700 instrument. Direct injections for intact protein HRMS as well as peptide MS/MS experiments were performed on a Thermo Scientific Oribtrap Eclipse Tribrid via Thermo Scientific HESI-Spray and Thermo Scientific FAIMS interface.

2. Chemical Procedures

2.1 Amino Acids

N-Cbz-L-Cys-OMe (**S1**)

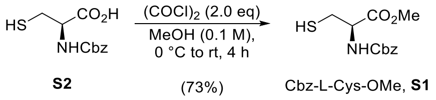

To a solution of *N*-Cbz-L-Cys (**S2**) (10.5 mmol, 2.68 g, 1.0 eq) in MeOH (100 mL) was added $(COCl)_2$ (21.0 mmol, 1.80 mL, 2.0 eq) dropwise at 0 °C and the reaction was slowly allowed to warm up to rt. After 4 h, brine was added (60 mL) and MeOH was concentrated under reduced pressure, followed by extraction with DCM (3 × 30 mL), drying over $MgSO_4$ and filtration. The organic phase was concentrated under reduced pressure and the crude compound was purified by automated liquid column chromatography (Interchim Puriflash, NP, hexane/EtOAc: 8/2 to 6/4) to yield *N*-Cbz-L-Cys-OMe (**S1**) (7.67 mmol, 2.06 g, 73%) as colorless solid.

$\boldsymbol{R}_\mathbf{f}$ = 0.11 (hexane/EtOAc/TEA: 79/20/1, UV, CAM)

^{1}H NMR (400 MHz, $CDCl_3$) δ 7.40 – 7.26 (m, 5H), 5.71 (d, *J* = 8.0 Hz, 1H), 5.11 (s, 2H), 4.67 (dt, *J* = 8.1, 5.2 Hz, 1H), 3.75 (s, 3H), 3.16 (d, *J* = 5.2 Hz, 2H), 1.77 – 1.58 (br, 1H) ppm.

^{13}C NMR (101 MHz, $CDCl_3$) δ 170.91, 155.78, 136.15, 128.67, 128.38, 128.30, 67.32, 53.31, 52.93, 41.18 ppm.

The analytical data is in accordance to literature.[1]

<u>*N*-Cbz-L-Lys-OMe (**S3**)</u>

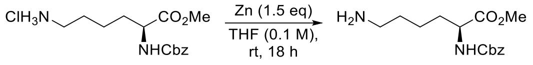

S4 (quant.) Cbz-L-Lys-OMe, **S3**

A suspension of *N*-Cbz-L-Lys-OMe • HCl (**S4**) (756 µmol, 250 mg, 1.0 eq) and zinc (1.16 mmol, 76.0 mg, 1.5 eq) in THF (1 mL) was stirred at rt. After 18 h, the mixture was filtered and the organic phase was concentrated under reduced pressure. The crude *N*-Cbz-L-Lys-OMe (**S3**) (quant.) was used without further purification.

^{1}H NMR (400 MHz, CD_3OD) δ 7.39 – 7.26 (m, 5H), 5.09 (s, 3H), 4.20 (dd, *J* = 9.3, 5.0 Hz, 1H), 3.72 (s, 3H), 2.91 (t, *J* = 7.6 Hz, 2H), 1.92 – 1.81 (m, 1H), 1.69 (dtdd, *J* = 18.3, 14.0, 9.4, 6.0 Hz, 4H), 1.46 (dtd, *J* = 15.1, 9.0, 6.4 Hz, 3H) ppm.

N-Cbz-L-Lys-OMe (**S3**) was synthesized according to a literature procedure.[2]

2.2 IAA

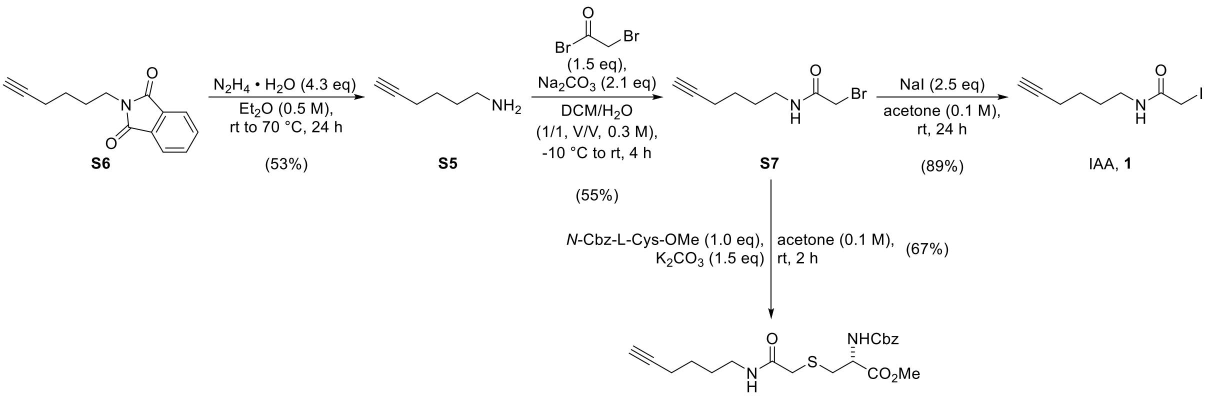

Cys-IAA, **12**

<u>Hex-5-yn-1-amine (**S5**)</u>

To a suspension of 2-(hex-5-yn-1-yl)isoindoline-1,3-dione (**S6**) (22.0 mmol, 5.00 g, 1.0 eq) in EtOH (45 mL) was added N_2H_4 • H_2O (94.6 mmol, 4.60 mL, 4.3 eq) dropwise and the mixture was heated to 70 °C. After 24 h, H_2O (50 mL) and aq. HCl (1 M, 100 mL) were added to adjust to pH4. The colorless participate was filtered off and the liquid phase was concentrated under reduced pressure. The residue was dissolved in aq. NaOH (6 M, 200 mL) to give a solution with pH10 followed by extraction with DCM (3 × 150 mL). The combined organic phase was washed with brine (150 mL). dried over Na_2SO_4 and filtered. The organic phase was concentrated under reduced pressure and the crude hex-5-yn-1-amine (**S5**) (11.7 mmol, 1.13 g, 53%) was used without further purification.

***R*$_f$** = 0.20 (DCM, $KMnO_4$, ninhydrin)

^{1}H NMR (300 MHz, $CDCl_3$) *δ* 2.95 (t, *J* = 7.7 Hz, 1H), 2.20 (h, *J* = 3.2 Hz, 2H), 1.94 (td, *J* = 2.7, 0.7 Hz, 1H), 1.55 (dtd, *J* = 4.3, 3.1, 2.2 Hz, 4H), 1.21 (s, 2H) ppm.

^{13}C NMR (100 MHz, D_2O) *δ* 87.64, 72.21, 41.57, 28.38, 27.05, 19.66 ppm.

The analytical data is in accordance to literature.[3]

<u>2-Bromo-*N*-(hex-5-yn-1-yl)acetamide (**S7**)</u>

To a solution of hex-5-yn-1-amine (**S5**) (1.50 mmol, 146 mg, 1.0 eq) in DCM (3.0 mL) and aq. $NaHCO_3$ (3.0 mL) and 2-bromoacetyl bromide (2.25 mmol, 196 µL, 1.5 eq) was added at –10 °C while stirring vigorously and the reaction was allowed to warm up to rt. After 4 h, the mixture was extracted with EtOAc (3 × 10 mL). The combined organic phase was washed with aq. $NaHCO_3$ (30 mL), aq. HCl (1 M,

30 mL) and brine (30 mL), dried over $MgSO_4$ and filtered. The organic phase was concentrated under reduced pressure and the crude compound was purified by liquid column chromatography (NP, DCM) to yield 2-bromo-*N*-(hex-5-yn-1-yl)acetamide (**S7**) (830 µmol, 180 mg, 55%) as colorless viscous liquid.

***R*f** = 0.60 (pentane/Et_2O: 7/3, UV, $KMnO_4$)

^{1}H NMR (300 MHz, $CDCl_3$) δ 6.53 (s, 1H), 3.88 (s, 2H), 3.32 (q, *J* = 6.9 Hz, 2H), 2.24 (td, *J* = 6.8, 2.7 Hz, 2H), 1.97 (t, *J* = 2.6 Hz, 1H), 1.76–1.62 (m, 2H), 1.62–1.55 (m, 2H) ppm.

^{13}C NMR (100 MHz, $CDCl_3$) δ 165.44, 83.95, 69.00, 39.78, 29.46, 28.45, 25.65, 18.17 ppm.

The analytical data is in accordance to literature.[4]

2-Iodo-*N*-(hex-5-yn-1-yl)acetamide (IAA, **1**)

To a solution of 2-bromo-*N*-(hex-5-yn-1-yl)acetamide (**S7**) (977 µmol, 213 mg, 1.0 eq) in acetone (9.2 mL) was added NaI (2.44 mmol, 366 mg, 2.5 eq). After 24 h, the organic phase was concentratedunder reduced pressure and H_2O (30 mL) was added followed by extraction with DCM (3 × 30 mL), drying over $MgSO_4$ and filtration. The combined organic phase was concentrated under reduced pressure and the crude compound was purified by liquid column chromatography (NP, pentane/Et_2O: 1/1) to yield 2-iodo-*N*-(hex-5-yn-1-yl)acetamide (IAA, **1**) (868 µmol, 230 mg, 89%) as colorless solid.

NOTE: The reaction procedure was performed in the absence of light.

***R*f** = 0.47 (pentane/Et_2O: 7/3, UV, $KMnO_4$)

^{1}H NMR (300 MHz, $CDCl_3$) δ 6.24 (s, 1H), 3.69 (s, 2H), 3.29 (td, *J* = 6.9, 5.8 Hz, 2H), 2.23 (td, *J* = 6.8, 2.7 Hz, 2H), 1.96 (t, *J* = 2.6 Hz, 1H), 1.70–1.61 (m, 2H), 1.61–1.51 (m, 2H) ppm.

^{13}C NMR (100 MHz, $CDCl_3$) δ 166.98, 84.01, 68.99, 40.02, 28.41, 25.65, 18.19, –0.22 ppm.

The analytical data is in accordance to literature.[5]

Methyl *N*-((Benzyloxy)carbonyl)-*S*-(2-(hex-5-yn-1-ylamino)-2-oxoethyl)-L-cysteinate (**12**)

To a solution of 2-bromo-*N*-(hex-5-yn-1-yl)acetamide (**S7**) (110 µmol, 24.0 mg, 1.0 eq) in acetone (1.1 mL) was added *N*-Cbz-L-Cys-OMe (**S1**) (110 µmol, 30.0 mg, 1.0 eq) and K_2CO_3 (165 µmol, 23.0 mg, 1.5 eq). After 2 h, aq. NH_4Cl (5 mL) was added, followed by extraction with EtOAC (3 × 10 mL), drying over $MgSO_4$ and filtration. The combined organic phase was concentrated under reduced pressure and the crude compound was purified by automated liquid column chromatography (Interchim Puriflash, NP, hexane/EtOAc: 4/6 to 7/3) to yield methyl *N*-((benzyloxy)carbonyl)-*S*-(2-(hex-5-yn-1-ylamino)-2-oxoethyl)-L-cysteinate (**12**) (74 µmol, 30 mg, 67%) as colorless dark red viscous liquid.

***R*f** = 0.29 (hexane/EtOAc: 4/6, $KMnO_4$)

^{1}H NMR (400 MHz, $CDCl_3$) δ 7.41 – 7.30 (m, 5H), 6.72 (s, 1H), 5.75 (d, *J* = 7.9 Hz, 1H), 5.13 (s, 2H), 4.61 (dt, *J* = 7.8, 5.3 Hz, 1H), 3.78 (s, 3H), 3.32 – 3.26 (m, 2H), 3.22 (d, *J* = 1.8 Hz, 2H), 3.07 (dd, *J* = 14.0, 4.8 Hz, 1H), 2.97 (dd, *J* = 13.9, 6.0 Hz, 1H), 2.22 (td, *J* = 6.8, 2.7 Hz, 2H), 1.95 (t, *J* = 2.6 Hz, 1H), 1.69 – 1.60 (m, 2H), 1.60 – 1.50 (m, 2H) ppm.

^{13}C NMR (101 MHz, $CDCl_3$) δ 170.95, 168.25, 155.99, 136.14, 128.71, 128.44, 128.25, 84.08, 68.93, 67.40, 53.86, 53.06, 39.43, 36.66, 35.74, 28.64, 25.78, 18.19 ppm.

HRMS (ESI pos): *m/z*: calcd for $C_{20}H_{27}N_2O_5S^+$ $[M+H]^+$: 407.1635; found: 407.1631.

2.3 APA

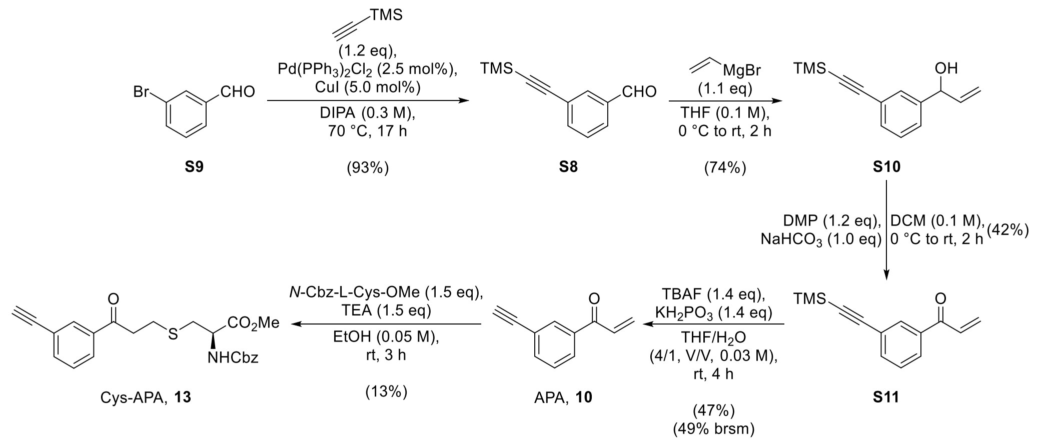

3-((Trimethylsilyl)ethynyl)benzaldehyde (**S8**)

To a solution of $Pd(OAc)_2$ (1.20 mmol, 269 mg, 2.0 mol%), PPh_3 (2.40 mmol, 629 mg, 5.0 mol%), 3-bromobenzaldehyde (**S9**) (60.0 mmol, 11.1 g, 1.0 eq) in TEA (60 mL) was added TMS-acetylene (66.0 mmol, 9.1 mL, 1.1 eq) and the mixture was heated to 89 °C. After 18 h, the mixture was allowed to cool to rt and filtered. The organic phase was concentrated under reduced pressure and the crude compound was purified by automated liquid column chromatography (Interchim Puriflash, NP, hexane/EtOAc: 9/1 to 7/3) to yield 3-((trimethylsilyl)ethynyl)benzaldehyde (**S8**) (48.6 mmol, 9.82 g, 81%) as colorless viscous liquid.

$\boldsymbol{R}_\mathbf{f}$ = 0.15 (hexane/EtOAc: 96/4, UV, $KMnO_4$)

^{1}H NMR (400 MHz, $CDCl_3$) δ 9.98 (s, 1H), 7.96 (td, J = 1.7, 0.6 Hz, 1H), 7.82 (dt, J = 7.7, 1.4 Hz, 1H), 7.70 (dt, J = 7.7, 1.4 Hz, 1H), 7.48 (t, J = 7.7 Hz, 1H), 0.26 (s, 9H) ppm.

^{13}C NMR (101 MHz, $CDCl_3$) δ 191.71, 137.58, 136.42, 133.60, 129.21, 129.12, 124.45, 103.44, 96.36, –0.02 ppm.

The analytical data is in accordance to literature.[6]

1-(3-((Trimethylsilyl)ethynyl)phenyl)prop-2-en-1-ol (**S10**)

To a solution of 3-((trimethylsilyl)ethynyl)benzaldehyde (**S8**) (48.5 mmol, 9.82 g, 1.0 eq) in THF (500 mL) was added vinylMgBr (53.5 mmol, 53.5 mL, 1.1 eq, 1.0 M in THF) dropwise at 0 °C and the reaction was allowed to warm to rt. After 2 h, aq. NH_4Cl (250 mL) was added, followed by extraction with EtOAc (3 × 300 mL) and drying over $MgSO_4$ and filtration. The organic phase was concentrated under reduced pressure and the crude compound was purified by automated liquid column chromatography (Interchim Puriflash, NP, hexane/EtOAc: 99/1 to 95/5) to yield 1-(3-((trimethylsilyl)ethynyl)phenyl)prop-2-en-1-ol (**S10**) (35.9 mmol, 8.27 g, 74%) as colorless viscous liquid.

$\boldsymbol{R}_\mathbf{f}$ = 0.23 (hexane/Et_2O: 96/4, UV, $KMnO_4$)

^{1}H NMR (400 MHz, $CDCl_3$) δ 7.49 – 7.47 (m, 1H), 7.38 (dt, J = 7.2, 1.7 Hz, 1H), 7.34 – 7.31 (m, 1H), 7.31 – 7.26 (m, 1H), 6.00 (ddd, J = 17.1, 10.3, 6.1 Hz, 1H), 5.34 (dt, J = 17.1, 1.4 Hz, 1H), 5.21 (dt, J = 10.3, 1.3 Hz, 1H), 5.17 (d, J = 6.2, 1.5 Hz, 1H), 0.25 (s, 9H) ppm.

^{13}C NMR (101 MHz, $CDCl_3$) δ 142.75, 139.92, 131.36, 129.99, 128.59, 126.63, 123.40, 115.75, 105.02, 94.43, 75.09, 0.09 ppm.

HRMS (EI pos): m/z: calcd for $C_{14}H_{18}OSi^+$ $[M]^+$: 230.1121; found: 230.1124.

1-(3-((Trimethylsilyl)ethynyl)phenyl)prop-2-en-1-one (**S11**)

To a solution of 1-(3-((trimethylsilyl)ethynyl)phenyl)prop-2-en-1-ol (**S10**) (5.78 mmol, 1.33 g, 1.0 eq) in DCM (60 mL) was added Dess-Martin periodinane (DMP) (6.93 mmol, 2.94 g, 1.2 eq) and $NaHCO_3$ (5.78 mmol, 485 mg, 1.0 eq) in sequence at 0 °C. After 4 h, aq. $Na_2S_2O_3$ (60 mL) was added, followed by extraction with EtOAc (3 × 70 mL), drying over $MgSO_4$ and filtration. The organic phase was concentrated under reduced pressure and the crude compound was purified by automated liquid column chromatography (Interchim Puriflash, NP, hexane/EtOAc: 100/0 to 97/3) to yield 1-(3-((trimethylsilyl)ethynyl)phenyl)prop-2-en-1-one (**S11**) (2.43 mmol, 548 mg, 42%) as colorless viscous liquid.

$\boldsymbol{R_f}$ = 0.62 (hexane/EtOAc: 96/4, UV)

^{1}H NMR (400 MHz, $CDCl_3$) δ 8.02 (t, J = 1.7 Hz, 1H), 7.88 (ddd, J = 7.9, 1.8, 1.2 Hz, 1H), 7.65 (dt, J = 7.6, 1.5 Hz, 1H), 7.42 (td, J = 7.8, 0.6 Hz, 1H), 7.13 (dd, J = 17.1, 10.6 Hz, 1H), 6.44 (dd, J = 17.1, 1.6 Hz, 1H), 5.95 (dd, J = 10.6, 1.6 Hz, 1H), 0.26 (s, 9H) ppm.

^{13}C NMR (101 MHz, $CDCl_3$) δ 190.44, 137.44, 136.19, 132.36, 132.28, 130.82, 128.79, 128.63, 123.96, 103.99, 95.76, 0.02 ppm.

HRMS (EI pos): *m/z*: calcd for $C_{13}H_{13}OSi^+$ $[M–CH_3]^+$: 213.0730; found: 213.0729.

1-(3-Ethynylphenyl)prop-2-en-1-one (APA, **10**)

To a solution of 1-(3-((trimethylsilyl)ethynyl)phenyl)prop-2-en-1-one (**S11**) (2.27 mmol, 518 mg, 1.0 eq) and KH_2PO_4 (3.18 mmol, 432 mg, 1.4 eq) in THF/H_2O (75 mL, 4/1, V/V) was added TBAF (3.20 mmol, 3.20 mL, 1.4 eq, 1.0 M in THF) dropwise. After 4 h, aq. NH_4Cl (75 mL) was added, followed by extraction with EtOAc (3 × 50 mL), drying over $MgSO_4$ and filtration. The organic phase was concentrated under reduced pressure and the crude compound was purified by automated liquid column chromatography (Interchim Puriflash, NP, hexane/EtOAc: 100/0 to 97/3) to yield 1-(ethynylphenyl)prop-2-en-1-one (APA, **10**) (1.07 mmol, 167 mg, 47%) as colorless viscous liquid.

$\boldsymbol{R_f}$ = 0.25 (hexane/EtOAc: 95/5, UV, $KMnO_4$)

^{1}H NMR (400 MHz, $CDCl_3$) δ 8.05 (t, J = 1.8 Hz, 1H), 7.92 (ddt, J = 7.9, 2.0, 1.0 Hz, 1H), 7.68 (dt, J = 7.7, 1.4 Hz, 1H), 7.45 (td, J = 7.8, 0.7 Hz, 1H), 7.12 (ddd, J = 17.2, 10.6, 0.7 Hz, 1H), 6.45 (dd, J = 17.1, 1.6 Hz, 1H), 5.96 (dd, J = 10.6, 1.5 Hz, 1H), 3.14 (s, 1H) ppm.

^{13}C NMR (101 MHz, $CDCl_3$) δ 190.35, 137.54, 136.36, 132.50, 132.22, 130.96, 128.99, 128.91, 122.95, 82.70, 78.47 ppm.

HRMS: (EI pos): *m/z*: calcd for $C_{11}H_7O^+$ $[M–H]^+$: 155.0491; found: 155.0487.

Methyl *N*-((Benzyloxy)carbonyl)-*S*-(3-(3-ethynylphenyl)-3-oxopropyl)-L-cysteinate (Cys-APA, **13**)

To a solution of 1-(3-ethynylphenyl)prop-2-en-1-one (APA, **10**) (64.0 µmol, 10.0 mg, 1.0 eq) in EtOH (1.3 mL) was added *N*-Cbz-L-Cys-OMe (**S1**) (36.0 µmol, 26.0 mg, 1.5 eq) and TEA (36.0 µmol, 10.0 µL, 1.5 eq) in sequence. After 2 h, aq. NH_4Cl (10 mL) was added, followed by extraction with DCM (3 × 10 mL), drying over $MgSO_4$ and filtration. The organic phase was concentrated under reduced pressure and the crude compound was purified by automated liquid column chromatography (Interchim Puriflash, NP, hexane/EtOAc: 9/1 to 8/2) to yield methyl *N*-((benzyloxy)carbonyl)-*S*-(3-(3-ethynylphenyl)-3-oxopropyl)-L-cysteinate (Cys-APA, **13**) (8.23 µmol, 3.50 mg, 13%) as yellowish viscous liquid.

$\boldsymbol{R_f}$ = 0.51 (hexane/EtOAc: 8/2, UV)

^{1}H NMR (400 MHz, $CDCl_3$) δ 8.05 (t, J = 1.8 Hz, 1H), 7.91 (dt, J = 7.9, 1.5 Hz, 1H), 7.68 (dt, J = 7.8, 1.4 Hz, 1H), 7.43 (t, J = 7.8 Hz, 1H), 7.38 – 7.29 (m, 5H), 5.67 (d, J = 8.0 Hz, 1H), 5.12 (s, 2H), 4.67 –

4.61 (m, 1H), 3.77 (s, 3H), 3.22 (t, J = 7.1 Hz, 2H), 3.14 (s, 1H), 3.06 (t, J = 4.6 Hz, 2H), 2.91 (t, J = 7.1 Hz, 2H) ppm.

^{13}C NMR (126 MHz, $CDCl_3$) δ 197.15, 171.27, 155.88, 136.72, 136.27, 131.90, 128.98, 128.69, 128.42, 128.38, 128.30, 128.27, 123.05, 82.61, 78.60, 67.30, 53.90, 52.90, 39.01, 35.22, 26.95 ppm.

HRMS (ESI pos): *m/z*: calcd for $C_{23}H_{24}NO_5S^+$ [M+H]$^+$: 426.1370; found: 426.1368.

2.4 CAPA

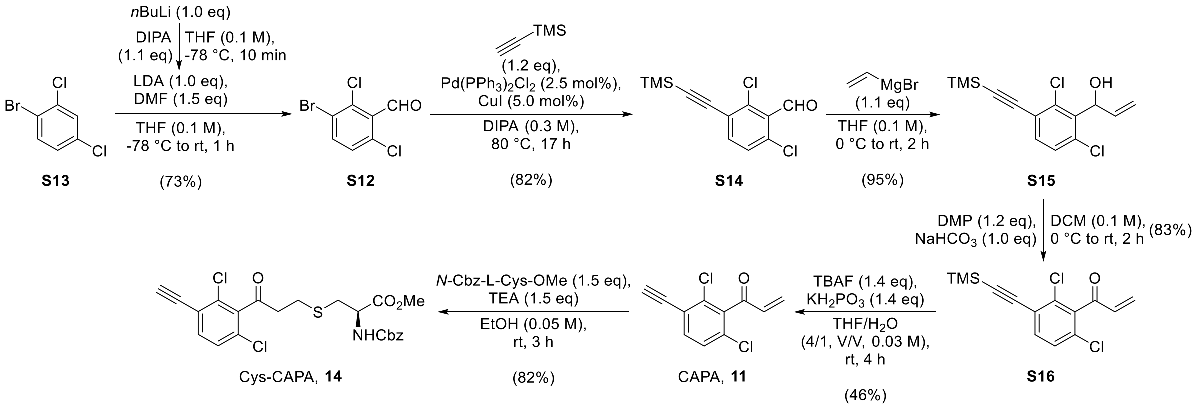

3-Bromo-2,6-dichlorobenzaldehyde (**S12**)

To a mixture of *N*-diisopropylamine (DIPA) (4.87 mmol, 680 µL, 1.1 eq) in THF (44 mL) was added *n*BuLi (4.43 mmol, 1.80 mL, 1.0 eq, 2.41 M in hexane) dropwise at –78 °C. After 10 min, 1-bromo-2,4-dichlorobenzene (**S13**) (4.43 mmol, 1.00 g, 1.0 eq) was added dropwise. After 15 min, DMF (6.64 mmol, 510 µL, 1.5 eq) was added dropwise and stirring was continued at –78 °C for 1 h. The reaction was allowed to warm to rt and aq. HCl (1 M, 20 mL) was added, followed by extraction with EtOAc (3 × 25 mL), drying over $MgSO_4$ and filtration. The organic phase was concentrated under reduced pressure and the crude compound was purified by automated liquid column chromatography (Interchim Puriflash, NP, hexane/EtOAc: 100/0 to 97/3) to yield 3-bromo-2,6-dichlorobenzaldehyde (**S12**) (3.23 mmol, 657 mg, 73%) as colorless viscous liquid.

***R*f** = 0.37 (hexane/EtOAc: 97/3, UV, $KMnO_4$)

^{1}H NMR (400 MHz, $CDCl_3$) δ 10.39 (s, 1H), 7.72 (d, J = 8.7 Hz, 1H), 7.28 (d, J = 8.6 Hz, 1H) ppm.

^{13}C NMR (101 MHz, $CDCl_3$) δ 188.46, 137.30, 136.61, 135.24, 132.81, 130.45, 123.60 ppm.

HRMS (EI pos): *m/z*: calcd for $C_7H_2BrCl_2O^+$ [M–H]$^+$: 250.8661; found: 250.8657.

2,6-Dichloro-3-((trimethylsilyl)ethynyl)benzaldehyde (**S14**)

To a solution of $Pd(PPh_3)_2Cl_2$ (10.0 µmol, 7.00 mg, 2.5 mol%), CuI (20.0 µmol, 4.00 mg, 5.0 mol%), 3-bromo-2,6-dichlorobenzaldehyde (**S13**) (390 µmol, 100 mg, 1.0 eq) in DIPA (1.3 mL) was added TMS-acetylene (470 µmol, 70.0 µL, 1.2 eq) and the mixture was heated to 70 °C. After 18 h, the mixture was allowed to cool to rt and filtered through Celite. The organic phase was concentrated under reduced pressure and the crude compound was purified by automated liquid column chromatography (Interchim Puriflash, NP, hexane/EtOAc: 100/0 to 97/3) to yield 2,6-dichloro-3-((trimethylsilyl)ethynyl) benzaldehyde (**S14**) (317 µmol, 86.0 mg, 81%) as colorless viscous liquid.

***R*f** = 0.34 (hexane/EtOAc: 97/3, UV, $KMnO_4$)

^{1}H NMR (400 MHz, $CDCl_3$) δ 10.44 (s, 1H), 7.55 (d, J = 8.4 Hz, 1H), 7.32 (d, J = 8.4 Hz, 1H), 0.28 (s, 9H) ppm.

^{13}C NMR (101 MHz, $CDCl_3$) δ 188.69, 138.58, 136.87, 135.77, 131.42, 129.39, 124.25, 103.62, 99.29, –0.15 ppm.

HRMS (EI pos): *m/z*: calcd for $C_{11}H_9Cl_2OSi^+$ $[M–CH_3]^+$: 254.9794; found: 254.9794.

1-(2,6-Dichloro-3-((trimethylsilyl)ethynyl)phenyl)prop-2-en-1-ol (**S15**)

To a solution of 2,6-dichloro-3-((trimethylsilyl)ethynyl)benzaldehyde (**S14**) (1.26 mmol, 341 mg, 1.0 eq) in THF (12 mL) was added vinylMgBr (1.35 mmol, 1.40 mL, 1.1 eq, 1.0 M in THF) dropwise at 0 °C and the reaction was allowed to warm up to rt. After 2 h, aq. NH_4Cl (20 mL) was added, followed by extraction with EtOAc (3 × 20 mL), drying over $MgSO_4$ and filtration. The organic phase was concentrated under reduced pressure and the crude compound was purified by automated liquid column chromatography (Interchim Puriflash, NP, hexane/EtOAc: 97/3 to 95/5) to yield 1-(2,6-dichloro-3-((trimethylsilyl)ethynyl)phenyl)prop-2-en-1-ol (**S15**) (1.20 mmol, 359 mg, 95%) as colorless viscous liquid.

$\boldsymbol{R_f}$ = 0.30 (hexane/EtOAc: 95/5, UV, $KMnO_4$)

^{1}H NMR (400 MHz, $CDCl_3$) δ 7.37 (d, J = 8.3 Hz, 1H), 7.25 (d, J = 9.7 Hz, 1H), 6.18 (ddd, J = 17.2, 10.5, 4.8 Hz, 1H), 6.04 – 5.99 (m, 1H), 5.30 – 5.22 (m, 2H), 3.16 (d, J = 10.5 Hz, 1H), 0.27 (s, 9H) ppm.

^{13}C NMR (101 MHz, $CDCl_3$) δ 137.38, 136.93, 136.34, 134.50, 133.06, 128.91, 123.71, 116.41, 102.10, 100.54, 72.70, –0.09 ppm.

HRMS (EI pos): *m/z*: calcd for $C_{14}H_{16}Cl_2OSi^+$ $[M]^+$: 298.0342; found: 298.0338.

1-(2,6-Dichloro-3-((trimethylsilyl)ethynyl)phenyl)prop-2-en-1-one (**S16**)

To a solution of 1-(2,6-dichloro-3-((trimethylsilyl)ethynyl)phenyl)prop-2-en-1-ol (**S15**) (1.20 mmol, 359 mg, 1.0 eq) in DCM (12 mL) was added DMP (1.44 mmol, 611 mg, 1.2 eq) and $NaHCO_3$ (1.20 mmol, 101 mg, 1.0 eq) in sequence at 0 °C. After 4 h aq. $Na_2S_2O_3$ (20 mL) was added, followed by extraction with DCM (3 × 15 mL), drying over $MgSO_4$ and filtration. The organic phase was concentrated under reduced pressure and the crude compound was purified by automated liquid column chromatography (Interchim Puriflash, NP, hexane/EtOAc: 100/0 to 97/3) to yield 1-(2,6-dichloro-3-((trimethylsilyl)ethynyl)phenyl)prop-2-en-1-one (**S16**) (1.04 mmol, 309 mg, 87%) as colorless viscous liquid.

$\boldsymbol{R_f}$ = 0.28 (hexane/EtOAc: 98/2, UV, $KMnO_4$)

^{1}H NMR (400 MHz, $CDCl_3$) δ 7.48 (d, J = 8.4 Hz, 1H), 7.28 (d, J = 8.3 Hz, 1H), 6.57 (dd, J = 17.7, 10.5 Hz, 1H), 6.19 (d, J = 10.5 Hz, 1H), 5.96 (d, J = 17.7 Hz, 1H), 0.27 (s, 9H) ppm.

^{13}C NMR (101 MHz, $CDCl_3$) δ 192.76, 137.71, 136.28, 134.32, 133.71, 133.67, 131.40, 127.95, 122.94, 102.92, 99.60, –0.13 ppm.

HRMS (EI pos): *m/z*: calcd for $C_{14}H_{14}Cl_2OSi^+$ $[M]^+$: 296.0185; found: 296.0181.

1-(2,6-Dichloro-3-ethynylphenyl)prop-2-en-1-one (CAPA, **11**)

To a solution of 1-(2,6-dichloro-3-((trimethylsilyl)ethynyl)phenyl)prop-2-en-1-one (**S16**) (794 µmol, 236 mg, 1.0 eq) and KH_2PO_4 (1.11 mmol, 151 mg, 1.4 eq) in THF/H_2O (25 mL, 4/1, V/V) was added TBAF (1.11 mmol, 1.10 mL, 1.4 eq, 1.0 M in THF) dropwise. After 4 h, aq. NH_4Cl (20 mL) was added, followed by extraction with EtOAc (3 × 25 mL), drying over $MgSO_4$ and filtration. The organic phase was concentrated under reduced pressure and the crude compound was purified by automated liquid column chromatography (Interchim Puriflash, NP, hexane/EtOAc: 100/0 to 97/3) to yield 1-(2,6-dichloro-3-ethynylphenyl)prop-2-en-1-one (CAPA, **11**) (365 µmol, 82.0 mg, 46%) as yellowish viscous liquid.

$\boldsymbol{R_f}$ = 0.44 (hexane/EtOAc: 95/5, UV, $KMnO_4$)

^{1}H NMR (400 MHz, $CDCl_3$) δ 7.52 (d, J = 8.4 Hz, 1H), 7.31 (d, J = 8.4 Hz, 1H), 6.59 (dd, J = 17.7, 10.5 Hz, 1H), 6.21 (d, J = 10.5 Hz, 1H), 5.98 (d, J = 17.7 Hz, 1H), 3.45 (s, 1H) ppm.

^{13}C NMR (101 MHz, $CDCl_3$) δ 192.61, 137.87, 136.20, 134.73, 133.89, 133.83, 132.02, 128.12, 121.99, 84.52, 78.82 ppm.

HRMS: *not found*

IR (Diamond ATR, MeCN) $\tilde{\upsilon}_{max}$ (cm^{-1}): 3096 (vw), 2926 (vw), 2854 (vw), 1674 (vw), 1642 (w), 1574 (w), 1550 (w), 1404 (s), 1380 (m), 1252 (m), 1218 (w), 1152 (w), 1112 (m), 1038 (m), 976 (m), 822 (m), 764 (m), 660 (w).

Methyl *N*-((Benzyloxy)carbonyl)-*S*-(3-(2,6-dichloro-3-ethynylphenyl)-3-oxopropyl)-L-cysteinate (Cys-CAPA **14**)

To a solution of 1-(2,6-dichloro-3-ethynylphenyl)prop-2-en-1-one (CAPA, **11**) (44.4 µmol, 19.0 mg, 1.0 eq) in EtOH (1.0 mL) was added *N*-Cbz-L-Cys-OMe (66.6 µmol, 18.0 mg, 1.5 eq) and TEA (66.6 µmol, 10.0 µL, 1.5 eq) in sequence. After 2 h, aq. NH_4Cl (10 mL) was added, followed by extraction with DCM (3 × 10 mL), drying over $MgSO_4$ and filtration. The organic phase was concentrated under reduced pressure and the crude compound was purified by automated liquid column chromatography (Interchim Puriflash, NP, hexane/EtOAc: 9/1 to 8/2) to yield methyl *N*-((benzyloxy)carbonyl)-*S*-(3-(2,6-dichloro-3-ethynylphenyl)-3-oxopropyl)-L-cysteinate (Cys-CAPA, **14**) (36.4 µmol, 18.0 mg, 82%) as dark red viscous liquid.

***R*$_f$** = 0.48 (hexane/EtOAc: 8/2, UV)

^{1}H NMR (400 MHz, $CDCl_3$) δ 7.49 (d, J = 8.4 Hz, 1H), 7.38 – 7.30 (m, 5H), 7.28 (d, J = 8.4 Hz, 1H), 5.66 (d, J = 7.9 Hz, 1H), 5.12 (s, 2H), 4.65 (dt, J = 7.9, 4.9 Hz, 1H), 3.78 (s, 3H), 3.45 (s, 1H), 3.11 – 3.04 (m, 4H), 2.91 (t, J = 7.2 Hz, 2H) ppm.

^{13}C NMR (101 MHz, $CDCl_3$) δ 199.39, 171.20, 155.81, 139.77, 136.19, 134.74, 132.58, 130.61, 128.69, 128.39, 128.29, 128.19, 122.05, 84.76, 78.60, 67.31, 53.78, 52.95, 43.74, 35.05, 25.88 ppm.

HRMS (ESI pos): *m/z*: calcd for $C_{23}H_{21}Cl_2O_5NSNa^+$ $[M+Na]^+$: 516.0410; found: 516.0413.

2.5 Cys-CAPA-DTB

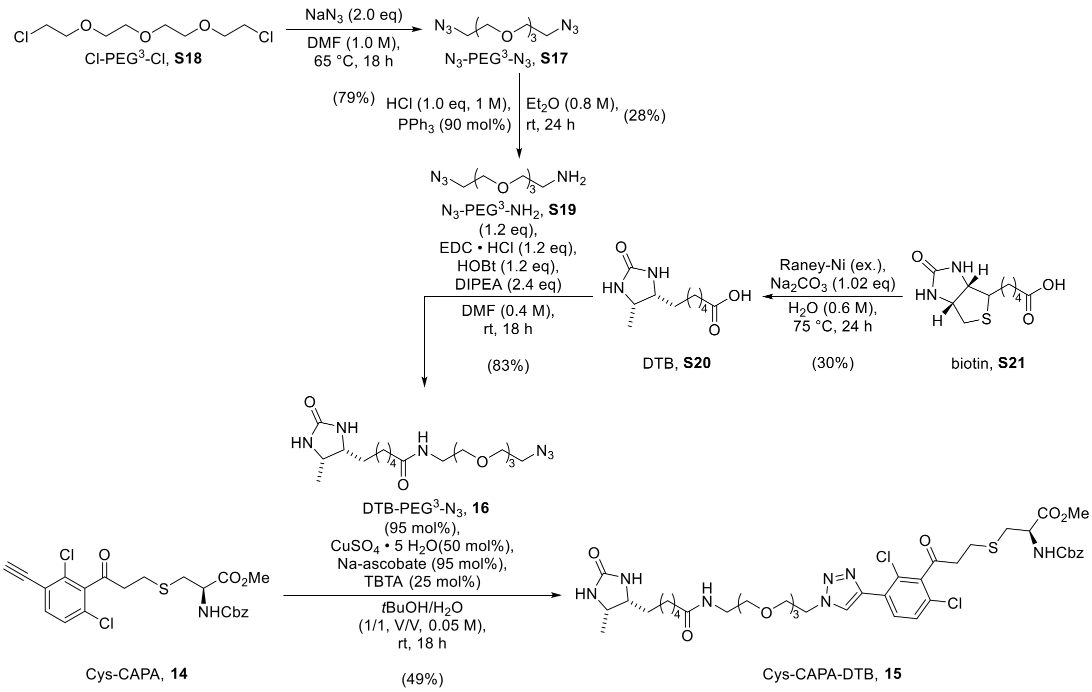

1-Azido-2-(2-(2-(2-azidoethoxy)ethoxy)ethoxy)ethane (N_3-PEG^3-N_3, **S17**)

To a solution of 1-chloro-2-(2-(2-(2-chloroethoxy)ethoxy)ethoxy)ethane (Cl-PEG^3-Cl, **S18**) (4.33 mmol, 850 µL, 1.0 eq) in DMF (5 mL) was added NaN_3 (8.66 mmol, 563 mg, 2.0 eq) and the reaction was heated to 65 °C. After 18 h, H_2O (10 mL) was added, followed by extraction with Et_2O (3 × 10 mL), drying over $MgSO_4$ and filtration. The organic phase was concentrated under reduced pressure and the crude compound was purified by automated liquid column chromatography (Interchim Puriflash, NP, hexane/EtOAc: 9/1 to 8/2) to yield 1-azido-2-(2-(2-(2-azidoethoxy)ethoxy)ethoxy)ethane (N_3-PEG^3-N_3, **S17**) (3.42 mmol, 838 mg, 79%) as colorless viscous liquid.

***R*f** = 0.12 (hexane/EtOAc: 9/1, $KMnO_4$)

^{1}H NMR (400 MHz, $CDCl_3$) δ 3.70 – 3.65 (m, 12H), 3.38 (t, J = 5.1 Hz, 4H) ppm.

^{13}C NMR (101 MHz, $CDCl_3$) δ 70.83, 70.16, 50.81 ppm.

The analytical data is in accordance to literature.[7]

2-(2-(2-(2-Azidoethoxy)ethoxy)ethoxy)ethan-1-amine (N_3-PEG^3-NH_2, **S19**)

To a solution of 1-azido-2-(2-(2-(2-azidoethoxy)ethoxy)ethoxy)ethane (N_3-PEG^3-N_3, **S17**) (3.40 mmol, 822 mg, 1.0 eq) in Et_2O (4.5 mL) was added aq. HCl (1 M, 3.40 mmol, 3.40 ML, 1.0 eq) dropwise and PPh_3 (3.03 mmol, 794 mg, 90 mol% in Et_2O (2.1 mL)) dropwise over 30 min in sequence. After 24 h, H_2O (10 mL) was added, followed by extraction with EtOAc (3 × 10 mL). The aq. phase was basified by addition of aq. NaOH (2 M) to adjust to pH9, followed by extraction with EtOAc (3 × 20 mL), drying over $MgSO_4$ and filtration. The organic phase was concentrated under reduced pressure and the crude compound was purified by automated liquid column chromatography (Interchim Puriflash, NP, DCM/MeOH/TEA: 89/10/1) to yield 2-(2-(2-(2-azidoethoxy)ethoxy)ethoxy)ethan-1-amine (N_3-PEG^3-NH_2, **S19**) (944 µmol, 203 mg, 28%) as yellowish viscous liquid.

***R*f** = 0.39 (DCM/MeOH/TEA: 89/10/1, ninhydrin)

^{1}H NMR (400 MHz, $CDCl_3$) δ 3.70 – 3.61 (m, 12H), 3.00 (t, J = 5.0 Hz, 2H), 1.69 (p, J = 7.3 Hz, 1H), 1.45 (h, J = 7.2 Hz, 1H), 1.36 (t, J = 7.3 Hz, 1H), 1.01 (t, J = 7.3 Hz, 1H) ppm.

^{13}C NMR (101 MHz, $CDCl_3$) δ 70.71, 70.64, 70.33, 70.10, 50.86, 45.95, 41.07, 24.31, 19.94, 13.84, 9.09 ppm.

The analytical data is in accordance to literature.[7]

Desthiobiotin (DTB, **S20**)

A solution of biotin (**S21**) (8.19 mmol, 2.00 g, 1.00 eq) and Na_2CO_3 (8.35 mmol, 885 mg, 1.02 eq) in H_2O (15 mL) was degassed by flushing N_2 through the solution. After 15 min, Raney-Ni (slurry in H_2O, 32 g, ex.) was added and the mixture was heated to 75 °C for 24 h. The solution was allowed to cool to rt and was filtered over Celite. The filtrate was removed, re-diluted in H_2O (100 mL) and acidified by adding aq. HCl (conc., 10 mL) until a participation occurred. The crude product was filtered off and recrystallized from H_2O (20 mL) whereby the residue was dissolved at 110 °C (oil bath, condenser) and the solution was allowed to slowly (oil bath) cool to rt, to yield desthiobiotin (DTB, **S20**) (2.46 mmol, 502 mg, 30%) as colorless solid.

***R*f** = 0.64 (DCM/MeOH: 9/1, $KMnO_4$)

^{1}H NMR (400 MHz, $(CD_3)_2SO$) δ 11.97 (s, 1H), 6.31 (s, 1H), 6.11 (s, 1H), 3.60 (p, *J* = 6.5 Hz, 1H), 3.52 – 3.42 (m, 1H), 2.19 (t, *J* = 7.4 Hz, 2H), 1.48 (p, *J* = 7.4 Hz, 2H), 1.41 – 1.11 (m, 6H), 0.95 (d, *J* = 6.3 Hz, 3H) ppm.

^{13}C NMR (101 MHz, $(CD_3)_2SO$) δ 174.48, 162.79, 54.94, 50.20, 33.55, 29.49, 28.59, 25.52, 24.38, 15.49 ppm.

The analytical data is in accordance to literature.[8]

N-(2-(2-(2-(2-Azidoethoxy)ethoxy)ethoxy)ethyl)-6-((4*R*,5*S*)-5-methyl-2-oxoimidazolidin-4-yl)hexan-amide (DTB-PEG^3-N_3, **16**)

To a solution of 2-(2-(2-(2-azidoethoxy)ethoxy)ethoxy)ethan-1-amine (N_3-PEG^3-NH_2, **S19**) (498 µmol, 109 mg, 1.2 eq) in DMF (1.1 mL) was added DTB, (**S20**) (415 µmol, 89.0 mg, 1.0 eq), EDC • HCl (498 µmol, 96.0 mg, 1.2 eq), HOBt (498 µmol, 76.0 mg, 1.2 eq) and DIPEA (997 µmol, 170 µL, 2.4 eq) in sequence. After 24 h, the organic phase was concentrated under reduced pressure and the crude compound was purified by automated liquid column chromatography (Interchim Puriflash, NP, DCM/MeOH: 1/0 to 85/15) to yield *N*-(2-(2-(2-(2-azidoethoxy)ethoxy)ethoxy)ethyl)-6-((4*R*,5*S*)-5-methyl-2-oxoimidazolidin-4-yl)hexan-amide (DTB-PEG^3-N_3, **16**) (3.44 µmol, 143 mg, 83%) as yellowish viscous liquid.

***R*f** = 0.47 (DCM/MeOH: 9/1, $KMnO_4$)

^{1}H NMR (400 MHz, $CDCl_3$) δ 6.42 (t, *J* = 5.6 Hz, 1H), 5.41 (s, 1H), 4.73 (s, 1H), 3.86 – 3.78 (m, 1H), 3.77 – 3.59 (m, 10H), 3.56 (dd, *J* = 5.6, 4.5 Hz, 2H), 3.44 (td, *J* = 5.6, 4.5 Hz, 2H), 3.39 (dd, *J* = 5.6, 4.5 Hz, 2H), 2.18 (t, *J* = 7.4 Hz, 2H), 1.90 (s, 1H), 1.66 (p, *J* = 7.1 Hz, 2H), 1.57 – 1.18 (m, 6H), 1.12 (d, *J* = 6.4 Hz, 3H) ppm.

^{13}C NMR (101 MHz, $CDCl_3$) δ 173.14, 163.67, 70.84, 70.69, 70.69, 70.32, 70.20, 70.12, 56.18, 51.54, 50.80, 39.28, 36.15, 29.60, 28.81, 26.08, 25.35, 15.92 ppm.

LRMS (ESI pos): *m/z*: calcd for $C_{18}H_{35}N_6O_5^+$ $[M+H]^+$: 415.3; found: 415.3.

The analytical data is in accordance to literature.[8]

Methyl *N*-((benzyloxy)carbonyl)-*S*-(3-(2,6-dichloro-3-(1-(18-((4*R*,5*S*)-5-methyl-2-oxoimidazolidin-4-yl)-13-oxo-3,6,9-trioxa-12-azaoctadecyl)-1*H*-1,2,3-triazol-4-yl)phenyl)-3-oxopropyl)-L-cysteinate (Cys-CAPA-DTB, **15**)

To a solution of methyl *N*-((benzyloxy)carbonyl)-*S*-(3-(2,6-dichloro-3-ethynylphenyl)-3-oxopropyl)-L-cysteinate (Cys-CAPA, **14**) (20 µmol, 10 mg, 1.0 eq), *N*-(2-(2-(2-(2-azidoethoxy)ethoxy)ethoxy)ethyl)-

6-((4*R*,5*S*)-5-methyl-2-oxoimidazolidin-4-yl)hexan-amide (DTB-PEG^3-N_3, **16**) (19 µmol, 8.0 mg, 95 mol%), $CuSO_4 \cdot 5\ H_2O$ (10 µmol, 3.0 mg, 50 mol%) and TBTA (5.1 µmol, 3.0 mg, 25 mol%) in *t*BuOH/H_2O (1/1, V/V, 400 µL) was added sodium ascorbate (19.2 mmol, 4 mg, 95 mol%). After 18 h, the mixture was purified by automated liquid column chromatography (Interchim Puriflash, RP, H_2O/MeCN: 1/0 to 0/1) to yield methyl *N*-((benzyloxy)carbonyl)-*S*-(3-(2,6-dichloro-3-(1-(18-((4*R*,5*S*)-5-methyl-2-oxoimidazolidin-4-yl)-13-oxo-3,6,9-trioxa-12-azaoctadecyl)-1*H*-1,2,3-triazol-4-yl)phenyl)-3-oxopropyl)-L-cysteinate (Cys-CAPA-DTB, **15**) (9.9 µmol, 9.0 mg, 49%) as colorless solid.

***R*f** = 0.51 (DCM/MeOH: 9/1, UV, $KMnO_4$)

^{1}H NMR (400 MHz, $CDCl_3$) δ 8.33 (s, 1H), 8.21 (d, *J* = 8.6 Hz, 1H), 7.43 (d, *J* = 8.6 Hz, 1H), 7.38 – 7.27 (m, 5H), 6.37 (s, 1H), 5.81 (d, *J* = 8.0 Hz, 1H), 5.23 (s, 1H), 5.12 (s, 2H), 4.69 – 4.59 (m, 4H), 3.94 (t, *J* = 5.0 Hz, 2H), 3.83 (q, *J* = 6.8 Hz, 1H), 3.78 (s, 3H), 3.70 – 3.52 (m, 7H), 3.51 (t, *J* = 5.1 Hz, 2H), 3.42 (tt, *J* = 7.1, 3.1 Hz, 2H), 3.18 – 3.00 (m, 4H), 2.94 (t, *J* = 7.1 Hz, 2H), 2.19 (t, *J* = 7.3 Hz, 2H), 1.72 (s, 3H), 1.62 (d, *J* = 7.5 Hz, 2H), 1.52 – 1.21 (m, 4H), 1.11 (d, *J* = 6.5 Hz, 3H) ppm.

^{13}C NMR (101 MHz, $CDCl_3$) δ 200.25, 173.38, 171.30, 163.59, 155.91, 142.44, 140.10, 136.27, 131.05, 129.60, 129.39, 128.74, 128.69, 128.37, 128.23, 127.15, 124.94, 70.73, 70.55, 70.49, 70.27, 70.13, 69.63, 67.29, 56.19, 53.90, 52.92, 51.61, 50.66, 43.91, 39.23, 36.19, 35.06, 29.48, 28.84, 26.10, 25.97, 25.28, 15.88 ppm.

NOTE: NMR signals in the ^{1}H and ^{13}C spectra are overlapping. See 2D NMR spectra below for detailed analysis.

HRMS (ESI pos): *m/z*: calcd for $C_{41}H_{56}Cl_2N_7O_{10}S^+$ $[M+H]^+$: 908.3181; found: 908.3182.

3. Kinetic Measurements

3.1 General Procedure for Kinetic Measurements of Sulfa-Michael Additions

To a solution of *N*-Cbz-L-amino acid methyl ester(s) (**S1**, **S2** and/or **S21**) (1.0 eq) and 4-nitrobenzonitrile (internal standard) in aq. PBS buffer pH7.4/MeCN (1/1, V/V) was added a cysteine-selective probe (**1**, **10** or **11**) (1.0 eq) at 0 °C (± 0.5, ice bath) open to air. Aliquots (100 µL) of the mixture (3.96 mL) were sampled at specific time intervals, diluted (9/1, V/V) with a mixture of H_2O/MeCN (1/1, V/V) containing TFA (0.1 V%). Analysis of the aliquots by reverse-phase HPLC (Thermo Fisher Dionex UltiMate 3000-Serie equipped with a DAD and Machery-Nagel EC 100/4 Nucleodur 100-3 C18 ec column) using H_2O/MeCN (A/B, V/V in %) containing TFA (0.1 V%) as eluent (gradient eluent A: linear 0 to 95 (–2 min), 95 (6 min), linear 95 to 70 (2 min), linear 70 to 0 (10 min), at 50 °C λ_{abs} = 254 nm).
Aliquots were taken at: 0, 0.2, 0.5, 1, 2, 4, 6, 8, 10, 15, 20, 25, 30, 40, 50, 60, 75, 90, 105, 120, 140, 160 and 180 min (unless otherwise stated).

NOTE: All measurements were performed in triplicates and were averaged. All compounds were handled as stock solutions (20.0 mM in MeCN). Quenching of aliquots with H_2O/MeCN (1/1, V/V) containing TFA (0.1 V%) was tested beforehand and led to a deviation of < 2% over 6 h. Aliquot at 0 min was prepared by direct addition of *N*-Cbz-L-amino acid methyl ester(s), internal standard and cysteine reactive probe in H_2O/MeCN (1/1, V/V) containing TFA (0.1 V%). Collected HPLC data (in mAU × min) was referenced to the internal standard and normalized. 4-Nitrobenzonitrile was used as internal standard.

3.2 General Procedure for Stability Experiments of Sulfa-Michael Adducts

Exact stochiometric amounts of *N*-Cbz-L-cysteine methyl ester–(click)probe adduct (**13**, **14** or **15**) in corresponding buffer solutions (pH4.0, 7.4 or 10.0) or PBS pH7.4/MeCN (505 mM) including GSH (1.0 eq) at 37 °C were measured by HPLC after 0, 3, 6 and 24 h. Analyzation of the aliquots (100 µL) by reverse-phase HPLC (Thermo Fisher Dionex UltiMate 3000-Serie equipped with a DAD and Machery-Nagel EC 100/4 Nucleodur 100-3 C18 ec column) using H_2O/MeCN (A/B, V/V in %) containing TFA (0.1 V%) as eluent (gradient eluent A: linear 0 to 95 (–2 min), 95 (6 min), linear 95 to 70 (2 min), linear 70 to 0 (10 min), at 50 °C λ_{abs} = 254 nm).

NOTE: All measurements were performed in triplicates and were averaged. All compounds were handled as stock solutions (20.0 mM in MeCN). Quenching of aliquots with H_2O/MeCN (1/1, V/V) containing TFA (0.1 V%) was tested beforehand and led to a deviation of < 2% over 6 h. Collected HPLC data (in mAU × min) was referenced to the internal standard and normalized.

3.3 General Procedure for Determining Second-Order Rate Constants

The second-order rate constants (k_2) were determined from the slope of the linear correlation obtained by plotting the inverse concentration of the cysteine-reactive probes IAA (**1**), APA (**10**) or CAPA (**11**) as a function of time. All measurements were conducted in triplicate and the reported second-order rate constants (k_2) are the average values of the three measurements. Data points corresponding to probe concentrations below 10% of the starting concentration were excluded from the kinetic analysis. The figures that present the reaction progression over time show averaged turnover–time plots (averaged out of three measurements) of the relative contributions for the sulfa-Michael additions of IAA (**1**), APA (**10**) or CAPA (**11**) in reactions with *N*-Cbz-L-Cys-OMe (**S1**), *N*-Cbz-L-Lys-OMe (**S3**) or *N*-Cbz-L-Ser-OMe (**S21**), which were determined via HPLC analysis.

3.4 Kinetic Measurements of Sulfa-Michael Additions, Plotted Data

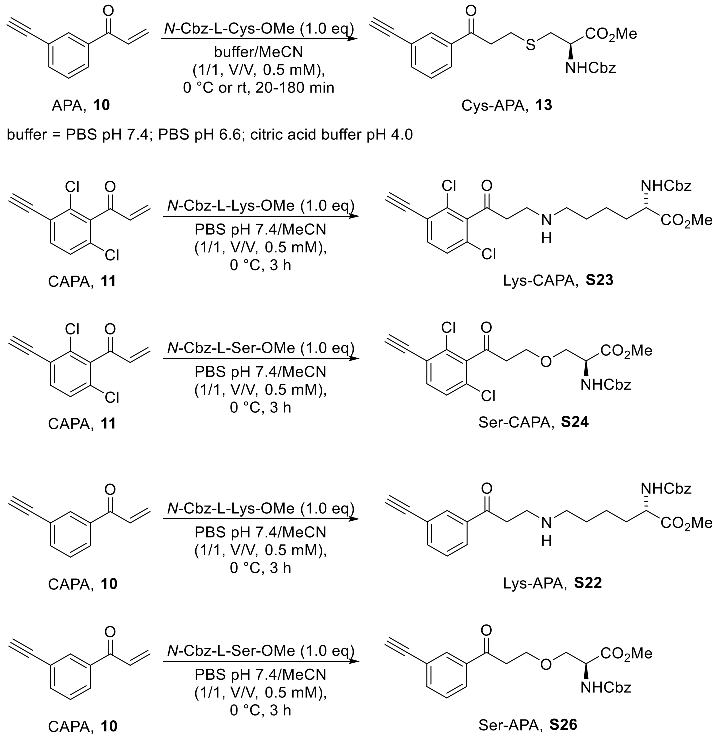

Scheme S1: Possible products of the reactions of APA (**10**) and CAPA (**11**) with *N*-Cbz-L-Cys-OMe (**S1**), *N*-Cbz-L-Lys-OMe (**S3**) or *N*-Cbz-L-Ser-OMe (**S21**) in under different conditions.

Table S1: Relative amounts of the kinetic measurements of sulfa-Michael additions of CAPA with *N*-Cbz-L-Cys-OMe at rt; pH 7.4.

	relative amount of [%]		
time [min]	Cys[1]	CAPA	Cys-CAPA
10	0.11	6.25	93.640
20	0.06	0.95	98.99

[1] Cys = *N*-Cbz-L-Cys-OMe

NOTE: Collected HPLC data (in mAU × min) was normalized.

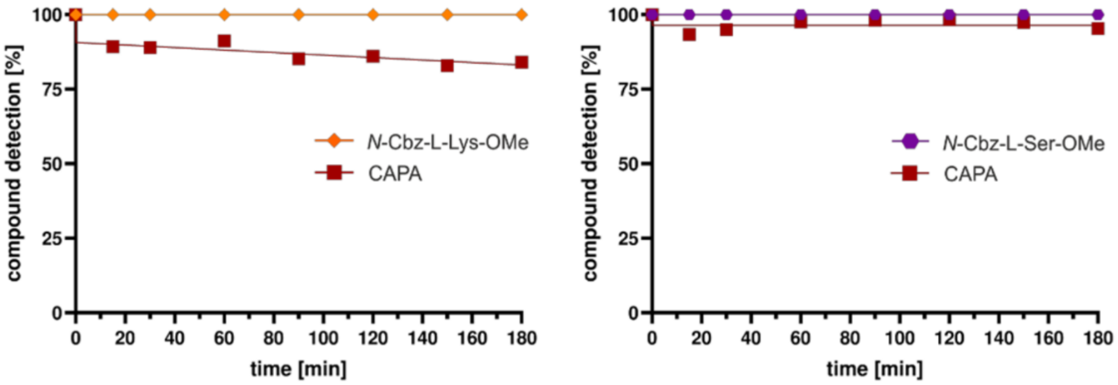

Figure S2 Progression of the reaction of CAPA (**11**) with *N*-Cbz-L-Lys-OMe (**S3**) or *N*-Cbz-L-Ser-OMe (**S21**)

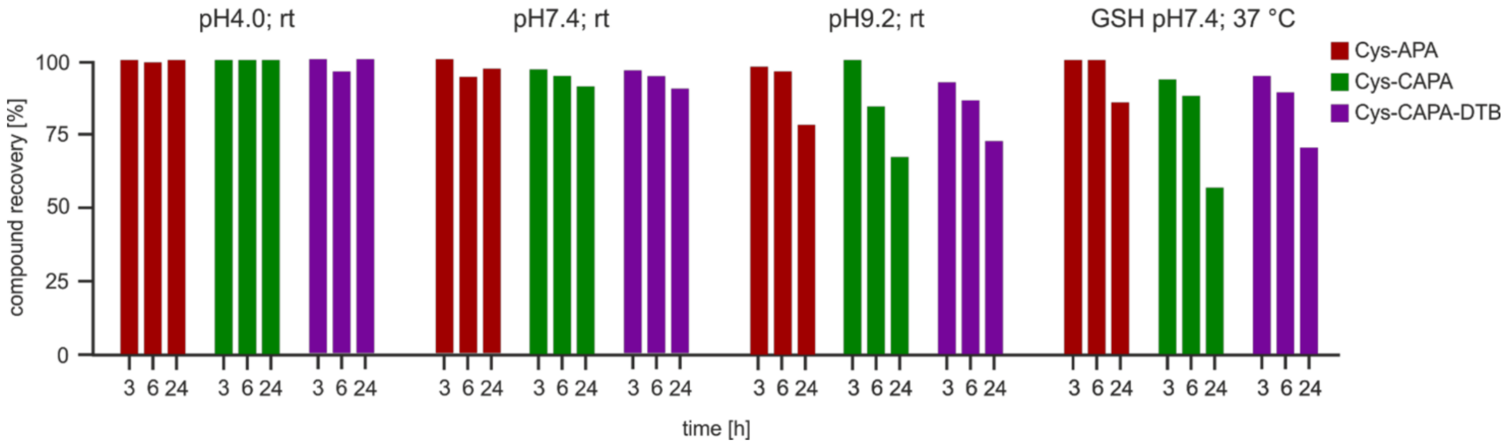

Figure S3: Stability experiments of Cys-APA (**13**), Cys-CAPA (**14**) and Cys-CAPA-DTB (**15**) at different pH levels over 24 h at rt and in the presence of GSH at 37 °C.

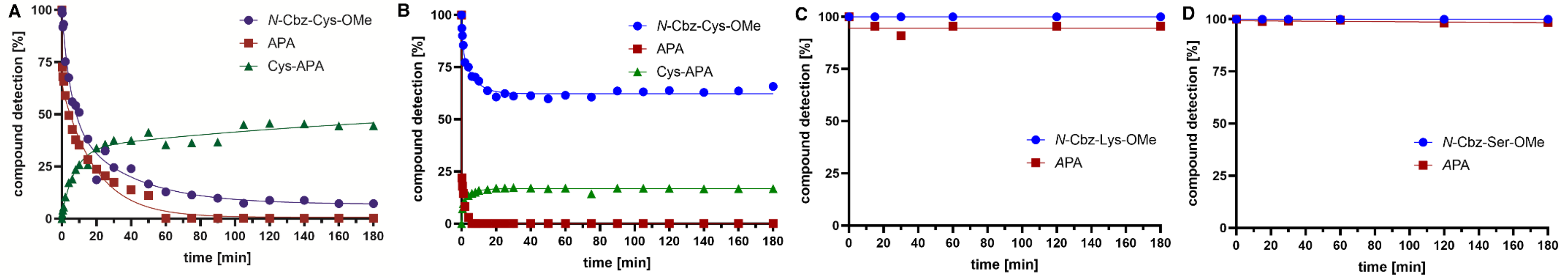

Figure S4A, B: Monitoring of the absolute amount of APA (**10**), N-Cbz-L-Cys-OMe (**S1**) and yield of the Michael adduct during the reaction at 0 °C in PBS at (A) pH6.6 and (B) pH8.2; C, D: Progression of the reaction of APA (**10**) with *N*-Cbz-L-Lys-OMe (**S3**) (C) or *N*-Cbz-L-Ser-OMe (**S21**) (D).

3.5 Kinetic Measurements of Sulfa-Michael Additions, Raw Data

Table S2: Relative amounts of the kinetic measurements of sulfa-Michael additions of IAA, APA and CAPA with *N*-Cbz-L-Cys-OMe at 0 °C; pH 7.4.

	relative amount of [%]								
time [min]	Cys[1]	IAA	Cys-IAA	Cys[1]	APA	Cys-APA	Cys[1]	CAPA	Cys-CAPA
0	100	100	0	100	100	0	100	100	0
0.2	97,7547334	95,9698894	0	90,4990632	84,9781509	7,64196781	80,5162456	89,4182778	11,0348772
0.5	92,2277061	96,2099517	0	68,5066102	73,0281413	17,9152124	61,2229062	77,446399	22,3941435
1	93,7973517	95,4124134	0	62,4283958	62,3372978	25,3576154	58,5848707	67,6273621	31,8932094
2	91,4956674	95,2940812	0,05954925	55,6710935	50,2388917	32,791391	39,8441711	50,1312929	44,6591035
4	90,8641747	94,9535122	0,36825948	44,7261468	37,8810324	41,508851	28,920185	34,8828	51,2862291
6	92,2199305	95,2049338	1,11034313	38,203601	29,79243	46,7604905	22,1514717	27,9848193	56,1403133
8	91,100746	93,923094	1,34277344	33,0983601	25,0354724	50,4605732	21,1222551	25,0157746	57,8300222
10	91,0348481	93,383123	1,78824124	32,6857826	21,2927085	53,8855646	20,3826298	23,8213518	61,7329975
15	89,4114259	92,3703854	2,68462305	25,5998407	14,4693325	59,461115	15,2678552	19,1736919	65,6564923
20	89,0737819	91,718876	3,52371422	25,9070922	10,7783837	61,7424924	15,9394627	16,9284701	65,3682853
25	88,4544777	91,0391619	4,34792169	21,0403918	7,36832324	63,2141535	13,5413074	15,0232251	66,8318726
30	87,5202166	89,7381609	4,94865882	20,6069643	5,65887896	64,9559972	14,1071088	13,9626684	68,3578124
40	85,6287128	87,839829	6,35861701	19,7116182	3,3097793	66,603715	12,7482505	12,3230534	68,8146027
50	82,3339146	86,04512	8,0826895	18,1487281	2,0385218	67,0715896	11,0173879	10,5073898	71,731631
60	82,4202917	84,6707143	9,75304925	17,6463618	1,29858598	69,4744387	9,38122023	8,66234037	77,9407495
75	80,0368985	82,1517826	11,7598167	17,8005936	0,66749289	68,8493253	9,90168871	7,99783285	76,3408833
90	78,2623514	80,401131	13,8228915	17,6016756	0,36038511	68,4440274	7,78669024	6,18234492	80,8803405
105	76,143349	78,1907616	16,1393408	17,06575	0,19984674	69,9729263	7,83188657	4,9072588	74,9063049
120	74,1297828	76,4151212	17,5459822	17,1905635	0,11083667	70,0781804	7,47505593	4,82049731	80,966184
140	72,7444782	74,1838992	20,3922663	16,7434789	0,05792053	69,2568602	7,52181862	4,6159008	84,9821098
160	69,1658182	71,8151541	23,2153987	16,6495162	0,04148863	69,0985209	6,56529279	3,66254279	79,9165263
180	67,6103715	69,6367727	25,053371	16,8383052	0,0287932	71,2672753	6,2127694	3,01721552	82,7166577

[1] Cys = *N*-Cbz-L-Cys-OMe

NOTE: All measurements were performed in triplicates and were averaged. Collected HPLC data (in mAU × min) was referenced to the internal standard and normalized. 4-Nitrobenzonitrile was used as internal standard.

Table S3: Relative amounts of the kinetic measurements of competitive sulfa-Michael additions of CAPA with *N*-Cbz-L-Cys-OMe at different pH's (6.6 and 8.2) at 0 °C.

	relative amount of [%]					
time [min]	Cys[1]	CAPA[2]	Cys-CAPA	Cys[1]	CAPA[3]	Cys-CAPA
0	100	100	0	100	100	0
0.2	87,0539206	92,2092263	2,83331942	56,8800523	63,0745353	34,4072274
0.5	81,0552423	87,8094723	7,60162829	39,0830551	44,3642233	46,7699748
1	78,7898397	83,7741129	10,1570097	29,9102841	33,6131906	55,3760448
2	67,6780912	76,7923283	14,7719508	25,9050823	23,2816277	61,6427909
4	64,7819851	65,3923874	24,2040633	17,834463	15,6535746	68,0309412
6	55,2630448	56,2838566	31,5931359	15,8671665	12,8554673	71,1496333
8	45,8199643	49,9959245	36,4728331	15,967172	10,7677733	71,8961748
10	46,7209444	43,9759517	40,2809238	15,6941334	9,76627926	72,6837615
15	34,224553	35,6039233	47,3367653	14,4614882	7,62697944	74,4943489
20	33,7402857	30,4887587	51,8071495	12,784158	6,42140105	75,2916971
25	30,8563799	25,3720636	54,3668367	12,6838804	5,60920753	75,9215996
30	28,5226058	22,6524509	57,2903896	11,2595729	4,8629862	75,9192532
40	26,487623	20,294624	59,4850339	10,8155451	3,90927218	77,0935526
50	23,7394566	18,1673056	62,2072013	10,4404829	2,97016082	77,8366868
60	23,2801159	16,1634892	63,2735808	9,51520927	2,60290841	79,8930008
75	20,9109363	14,3813557	66,4410057	8,83821693	1,86939859	79,6431438
90	19,6815599	12,6297703	67,2101839	8,74293535	1,45101188	81,6154964
105	17,2462696	11,6172433	69,6922631	7,9744778	1,19244904	81,8018416
120	17,4116715	10,4537087	70,8903736	7,8667248	0,88062288	80,6592691
140	16,0368827	9,61980048	73,1106929	7,73508024	0,64950345	84,080789
160	15,2071128	8,60113114	74,549425	7,54095407	0,5157197	85,7783041
180	13,1543795	7,95473936	75,5200639	7,62515222	0,38391972	90,8990271

[1] Cys = *N*-Cbz-L-Cys-OMe

[2] measurement at pH 6.6

[3] measurement at pH 8.2

NOTE: All measurements were performed in triplicates and were averaged. Collected HPLC data (in mAU × min) was referenced to the internal standard and normalized. 4-Nitrobenzonitrile was used as internal standard.

Table S4: Relative amounts of the kinetic measurements of competitive sulfa-Michael additions of APA with *N*-Cbz-L-Cys-OMe at different pH's (6.6 and 8.2) at 0 °C.

	relative amount of [%]					
time [min]	Cys[1]	APA[2]	Cys-APA	Cys[1]	APA[3]	Cys-CAPA
0	100	100	0	100	100	0
0.2	95,7441081	79,3009068	1,49642794	91,22268	21,2480894	0,0573639
0.5	88,2410323	72,241822	2,73050301	83,1928591	16,6892704	7,20044788
1	86,5889343	69,5384615	3,72860397	80,7510669	13,2905718	9,37745326
2	74,0302586	63,5169892	10,1409678	65,7292651	0,06775478	9,98109232
4	64,3822469	52,5729344	17,3150858	65,3611628	0,06737533	15,3711188
6	53,6508819	46,2923593	19,7775336	65,7292651	0,06775478	15,4576862
8	52,3074793	40,7505714	23,858765	67,0577823	0,08092593	16,2848962
10	46,7282043	37,4070709	26,0107352	61,9053158	0,06717157	16,9941886
15	37,3149442	30,9967272	27,4349115	48,6913773	0,01338449	17,1881096
20	22,6724754	27,0440043	34,0384998	45,3874269	0,01336745	17,7334819
25	30,3392411	22,7679937	35,6808391	48,573167	0,026704	17,7264712
30	24,2121998	19,8353483	37,0394943	48,539498	0,02668549	17,6737406
40	21,5466193	16,1645998	38,1790204	45,2095191	0,02663011	17,4218144
50	15,9452089	13,4267182	41,8963354	45,2721506	0,026667	17,621083
60	12,3569394	4,84709458	35,5261446	50,0072746	0,02660557	17,2310315
75	10,5035369	4,05780712	36,1328419	50,0360906	0,03993136	17,3351
90	8,29281043	0,18026653	37,857211	54,9479515	0,05330939	17,1820517
105	6,89873121	0,15215838	43,8036783	53,3256818	0,03997743	17,1666053
120	7,33140398	0,11343834	43,7901153	53,1967869	0,0398808	17,1251114
140	7,23464567	0,10795338	43,4244709	53,0563293	0,0397755	16,7717874
160	6,05851908	0,1802329	43,9966606	54,7332123	0,03982579	16,9405353
180	6,02228154	0,16106783	44,2312411	59,6998545	0,05322341	16,966125

[1] Cys = *N*-Cbz-L-Cys-OMe

[2] measurement at pH 6.6

[3] measurement at pH 8.2

NOTE: All measurements were performed in triplicates and were averaged. Collected HPLC data (in mAU × min) was referenced to the internal standard and normalized. 4-Nitrobenzonitrile was used as internal standard.

Table S5: Relative amounts of CAPA in a reaction with *N*-Cbz-L -Lys-OMe and CAPA in a reaction with *N*-Cbz-L-Ser-OMe at pH 7.4 and 0 °C.

time [min]	relative amount of [%]			
	Lys[1]	CAPA	Ser[1]	CAPA
0	100	100	100	100
15	>99.99	89,1627377	>99.99	93,3347705
30	>99.99	88,9896417	>99.99	94,9729916
60	>99.99	91,1622889	>99.99	97,5695703
90	>99.99	85,1788254	>99.99	98,0504699
120	>99.99	85,9737417	>99.99	98,3928755
150	>99.99	82,8630068	>99.99	97,3940495
180	>99.99	84,019523	>99.99	95,2703781

[1] Lys = *N*-Cbz-L-Lys-OMe; Ser = *N*-Cbz-L-Ser-OMe

NOTE: All measurements were performed in triplicates and averaged. Collected HPLC data (in mAU × min) was referenced to the internal standard and normalized. 4-Nitrobenzonitrile was used as internal standard. Apart from *N*-Cbz-L-Lys-OMe and *N*-Cbz-L-Ser-Ome, the relative integrals for the reaction components and products correlated to the amounts present in the sample. Even if it was not consumed, the integral values of *N*-Cbz-L-Lys-OMe and *N*-Cbz-L-Ser-OMe were subject to fluctuations between measurements. This was confirmed by repetitive measurements of pre-defined amounts of *N*-Cbz-L-Lys-OMe and *N*-Cbz-L-Ser-OMe. In case values were measured that exceeded the starting point, the values are represented as >99.99%.

Table S6: Relative amounts of APA in a reaction with *N*-Cbz-L -Lys-OMe and APA in a reaction with *N*-Cbz-L-Ser-OMe at pH 7.4 and 0 °C.

time [min]	relative amount of [%]			
	Lys[1]	APA	Ser[1]	APA
0	100	100	100	100
15	99,9825104	95,4689121	99,9711164	98,8911819
30	>99.99	90,8616705	99,9905716	99,0721444
60	99,981596	95,4959888	99,9905716	99,5329565
90	>99.99	95,4741845	99,9905452	98,1889738
120	99,9740949	95,5154028	99,9711164	98,4437062
150	>99.99	100	100	100
180	99,9825104	95,4689121	99,9711164	98,8911819

[1] Lys = *N*-Cbz-L-Lys-OMe; Ser = *N*-Cbz-L-Ser-OMe

NOTE: All measurements were performed in triplicates and averaged. Collected HPLC data (in mAU × min) was referenced to the internal standard and normalized. 4-Nitrobenzonitrile was used as internal standard. Apart from *N*-Cbz-L-Lys-OMe and *N*-Cbz-L-Ser-Ome, the relative integrals for the reaction components and products correlated to the amounts present in the sample. Even if it was not consumed, the integral values of *N*-Cbz-L-Lys-OMe and *N*-Cbz-L-Ser-OMe were subject to fluctuations between measurements. This was confirmed by repetitive measurements of pre-defined amounts of *N*-Cbz-L-Lys-OMe and *N*-Cbz-L-Ser-OMe. In case values were measured that exceeded the starting point, the values are represented as >99.99%.

Table S7: Stability measurements of the different adducts over 24 h at different pH's.

time [h]	relative amount of Cys-APA at [%]			relative amount of Cys-CAPA at [%]			relative amount of Cys-CAPA-DTB at [%]		
	pH4.0	pH7.4	pH9.2	pH4.0	pH7.4	pH9.2	pH4.0	pH7.4	pH9.2
3	>99.99	>99.99	97,89547631	>99.99	96,78735356	>99.99	>99.99	96,23299026	92,38176833
6	99,27266492	94,09207813	96,29161971	>99.99	94,63146781	84,10089071	95,78940932	94,138725	86,19743567
24	>99.99	96,72412806	78,08709799	>99.99	90,91610093	66,96342063	>99.99	89,93835239	72,31261786

NOTE: All measurements were performed in triplicates and averaged. Collected HPLC data (in mAU × min) was referenced to the internal standard and normalized. 4-Nitrobenzonitrile was used as internal standard.

Table S8: Stability measurements of the different adducts over 24 h in the presence of glutathione (GSH) at pH7.4.

time [h]	relative amount of adducts in the presence of GSH at pH 7.4 [%]		
	Cys-APA	Cys-CAPA	Cys-CAPA-DTB
3	>99.99	93,40012087	94,4538872
6	>99.99	87,67974978	88,98469814
24	85,52091606	56,62104825	70,0991314

NOTE: All measurements were performed in triplicates and averaged. Collected HPLC data (in mAU × min) was referenced to the internal standard and normalized. 4-Nitrobenzonitrile was used as internal standard.

4. Peptide Modifications and Digests

Nitric Oxide Synthase Blocking Peptide (599-613) CAPA Covalent Modification (**NOS BP-CAPA**, **18**)

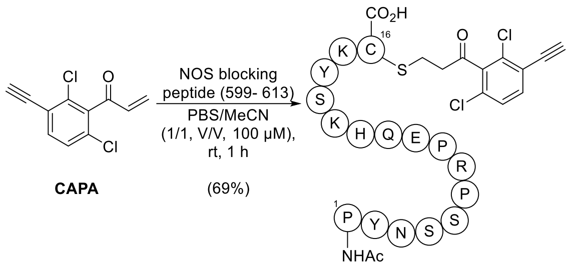

To a solution of NOS BP (599-613) (**X**) (9.08 µmol, 17.8 mg, 1.0 eq) in PBS pH7.4/MeCN (27 mL, 2/1, V/V) was added 1-(2,6-dichloro-3-ethynylphenyl)prop-2-en-1-one (**CAPA**) (9.08 µmol, 2.04 mg, 1.0 eq). After 1 h, the organic phase was concentrated under reduced pressure and the crude compound was purified by semi-preparative HPLC (Thermo Scientific Dionex UltiMate 3000-Serie equipped with DAD and Macherey-Nagel VP 250/10 Nucleodur 100-5 C18 ec column) using H_2O/MeCN (A/B, V/V in %) containing TFA (0.1 V%) as eluent (gradient eluent A: linear 0 to 95 (–2 min), 95 (12 min), linear 95 to 75 (2 min), linear 75 to 55 (16 min) linear 55 to 0 (1 min), at 25 °C λ_{abs} = 280 nm) to yield the modified peptide (**NOS BP-CAPA**) (6.27 µmol, 13.8 mg, 69%) as a dark red solid.

HRMS (ESI pos): *m/z*: calcd for $C_{96}H_{135}Cl_2N_{25}O_{28}S^+$ $[M+2H]^{2+}$: 1093.9498; found: 1093.9490.

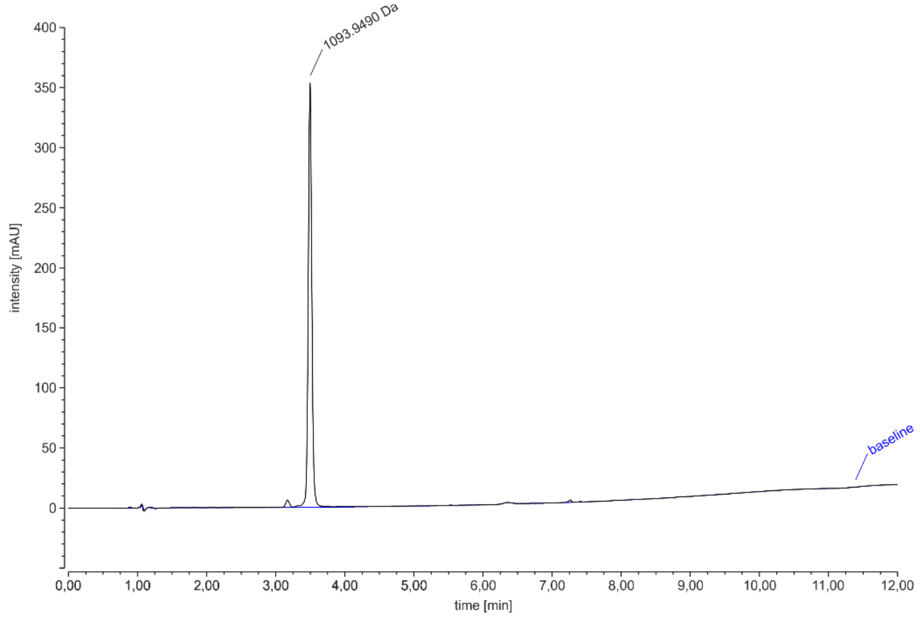

Figure S5: HPLC chromatogram of purified **NOS BP-CAPA** and baseline of blank measurement at 254 nm.

Trypsin Digest of NOS BP-CAPA to validate cysteine-selective binding by specifically cleaving the C-terminal lysine-cysteine bond to yield the CAPA-modified cysteine:

To the modified peptide **NOS BP-CAPA** (200 µM in PBS/MeCN; 1/1, V/V, 100 µL) was added trypsin (Trypsin-EDTA 10X purchased from Biowest, ID number MS01W91001, diluted 20/1 with PBS pH7.4, 200 µL, excess) and the solution was stirred at rt. After 1 h, the reaction was analyzed by MS.

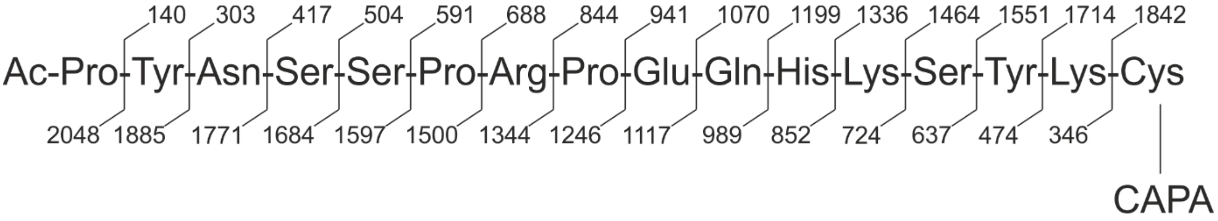

LC-HRMS (ESI pos): *m/z*: calcd for $C_{14}H_{14}Cl_2NO_3S^+$ $[M+H]^+$: 346.0066; found: 346.0030.

An MS/MS experiment with **NOS BP-CAPA** detected several amino acid/peptide fragments including the modified in high resolution (Figure S6).

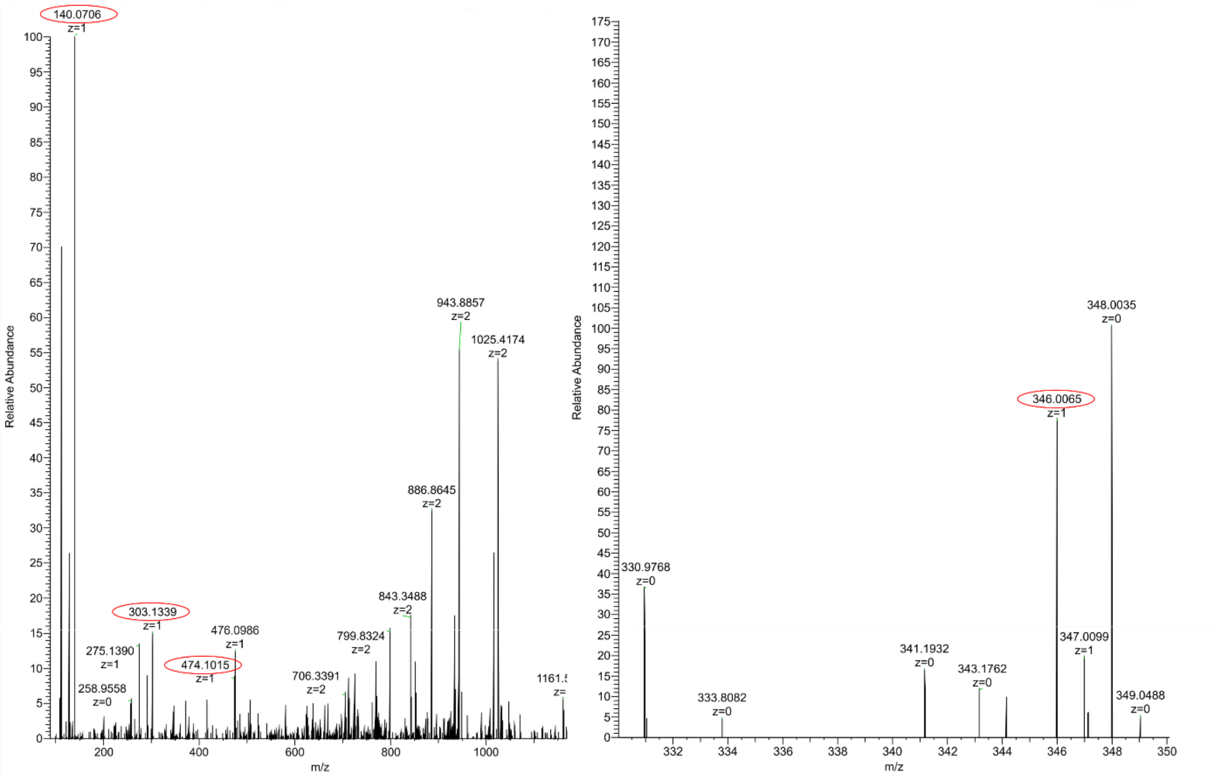

Figure S6: Full MS/MS spectrum of modified peptide **NOS BP-CAPA** (left) and zoomed-in section of the 330-350 m/z region (right).

Laminin (925-933) CAPA Covalent Modification (**Laminin-CAPA**, **17**)

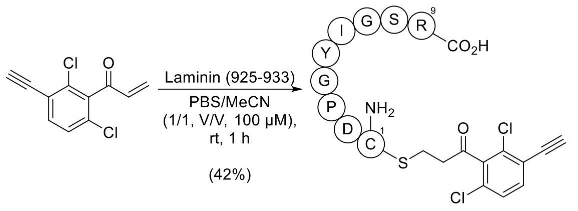

To a solution of Laminin (925-933) (**X**) (21.5 µmol, 20.8 mg, 1.0 eq) in PBS pH7.4/MeCN (20 mL, 1/1, V/V) was added 1-(2,6-dichloro-3-ethynylphenyl)prop-2-en-1-one (**CAPA**) (21.5 µmol, 1.08 mL, 20.0 mM in MeCN, 1.0 eq). After 1 h, the organic phase was concentrated under reduced pressure and the crude compound was purified by semi-preparative HPLC (Thermo Scientific Dionex UltiMate 3000-Serie equipped with DAD and Macherey-Nagel VP 250/10 Nucleodur 100-5 C18 ec column) using H_2O/MeCN (A/B, V/V in %) containing TFA (0.1 V%) as eluent (gradient eluent A: linear 0 to 95 (–2 min), 95 (12 min), linear 95 to 75 (2 min), linear 75 to 55 (16 min) linear 55 to 0 (1 min), at 25 °C λ_{abs} = 280 nm) to yield the modified peptide (**Laminin-CAPA**) (8.98 µmol, 10.7 mg, 42%) as a dark red solid.

LC-HRMS (ESI pos): *m/z*: calcd for $C_{51}H_{69}Cl_2N_{12}O_{15}S^+$ [M+H]$^+$: 1191.4098; found: 1191.4175.

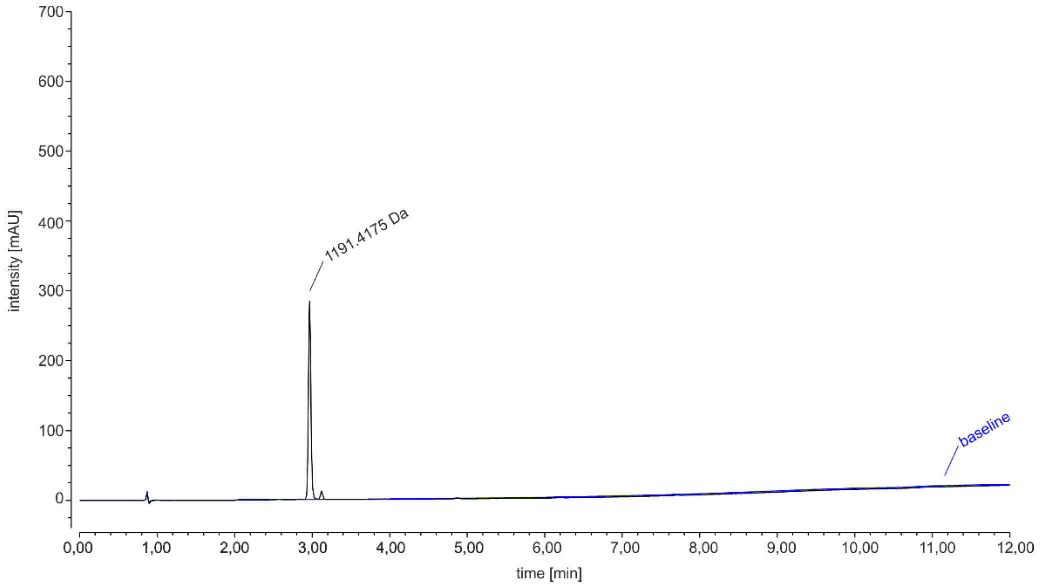

Figure S7: HPLC chromatogram of purified **Laminin-CAPA** and baseline of blank measurement at 254 nm.

Endoproteinase Asp-N digest of **Laminin-CAPA** to validate cysteine-selective binding by specifically cleaving the N-terminal cysteine-aspartate bond to yield the CAPA-modified cysteine.

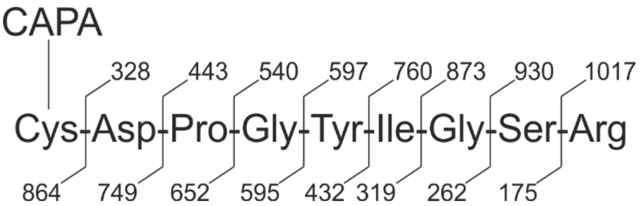

To the modified peptide **Laminin-CAPA** (200 µM in PBS/MeCN; 1/1, V/V, 50 µL) was added endoproteinase Asp-N (endoproteinase Asp-N, sequencing grad from Roche purchased from Sigma-Aldrich, product number 11420488001, 2 µg diluted in 50 µL H_2O, 10 µL, excess) and the solution was stirred at rt. After 5 h, the reaction was analyzed by MS.

LC-HRMS (ESI pos): *m/z*: calcd for $C_{14}H_{14}Cl_2NO_3S^+$ [M+H]$^+$: 346.0066; found: 346.0567.

5. Protein Modification

TrxA C33S Covalent Modification (**TrxA C33S-CAPA**) with CAPA (1.1 eq)

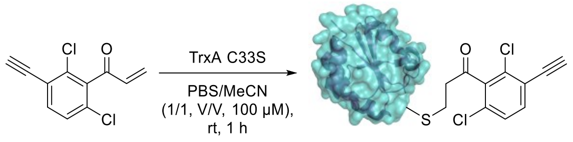

Note: TrxA C33S Figure, PDB: 6H1Y

To a solution of TrxA C33S (59.6 µL, 100 µM in PBS pH7.4/MeCN (2/1, V/V), 1.0 eq) was added 1-(2,6-dichloro-3-ethynylphenyl)prop-2-en-1-one (**CAPA**) (0.40 µL, 20 mM in MeCN, 1.1 eq). After 1 h, the reaction was quenched with H_2O/MeCN (1/1, V/V) containing TFA (0.1 v%) (60 µL) and analyzed by direct injection MS (Figure S8).

HRMS (HESI pos): *m/z*: calcd for $C_{596}H_{924}Cl_2N_{155}O_{171}S_4^+$ $[M+H]^+$: 13186.6632; found: 13186.6544.

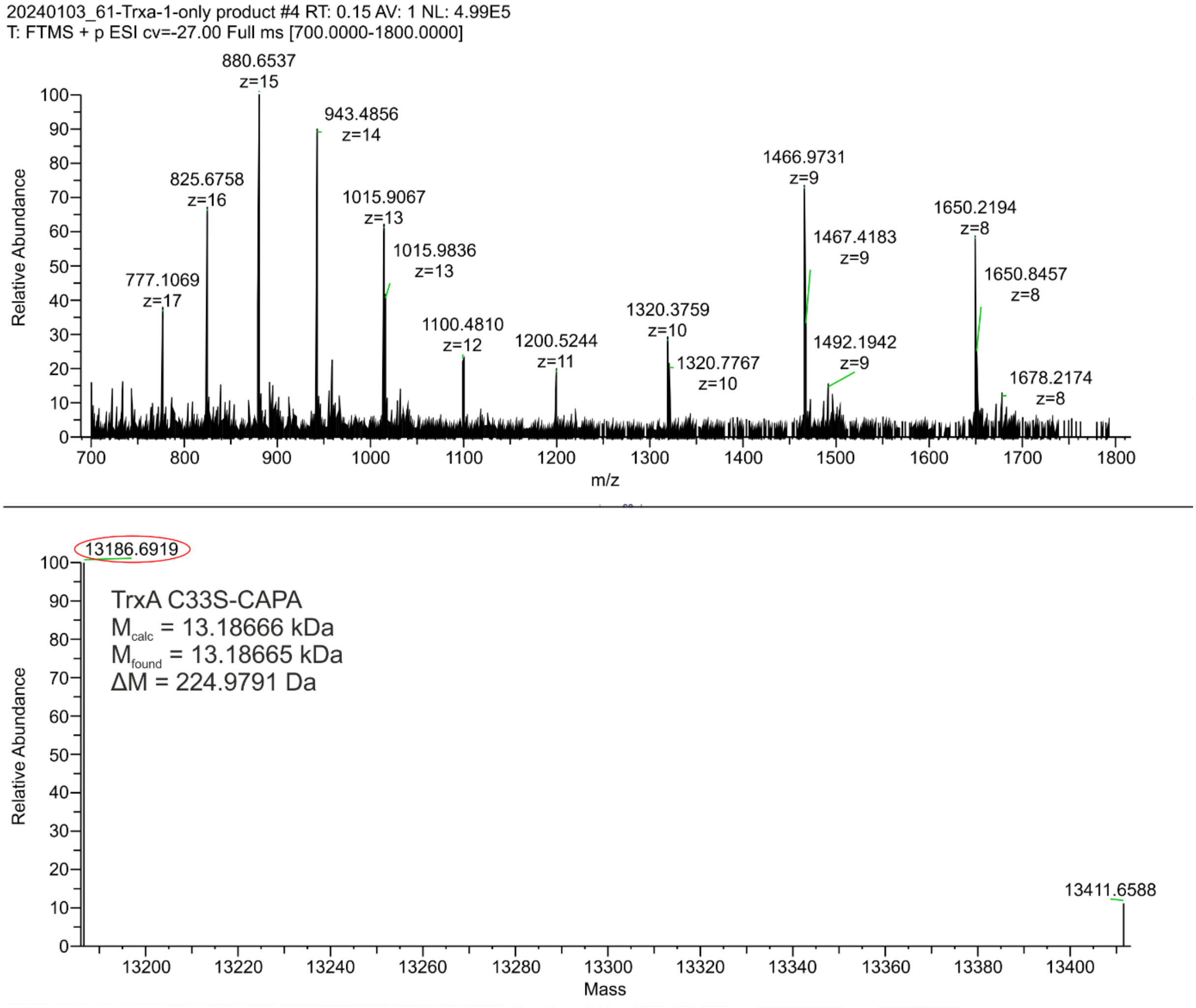

Figure S8: MS spectrum of modified protein **TrxA C33S-CAPA** (reaction with TrxA C33S (1.0 eq) and CAPA (1.1 eq). Measured by direct injection on a Thermo Scientific Oribtrap Eclipse Tribrid via Thermo Scientific HESI-Spray and Thermo Scientific FAIMS interface.

TrxA C33S Covalent Modification (**TrxA C33S-CAPA**) with CAPA (0.5 eq)

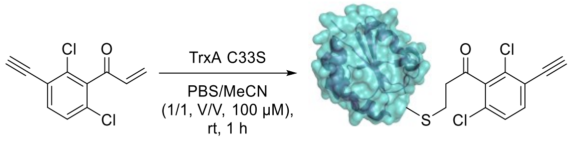

Note: TrxA C33S Figure, PDB: 6H1Y

To a solution of TrxA C33S (59.6 µL, 100 µM in PBS pH7.4/MeCN (2/1, V/V), 1.0 eq) was added 1-(2,6-dichloro-3-ethynylphenyl)prop-2-en-1-one (**CAPA**) (0.18 µL, 20 mM in MeCN, 0.5 eq). After 1 h, the reaction was quenched with H_2O/MeCN (1/1, V/V) containing TFA (0.1 v%) (60 µL) and analyzed by direct injection MS (Figure S9) and LCMS (Figure S10).

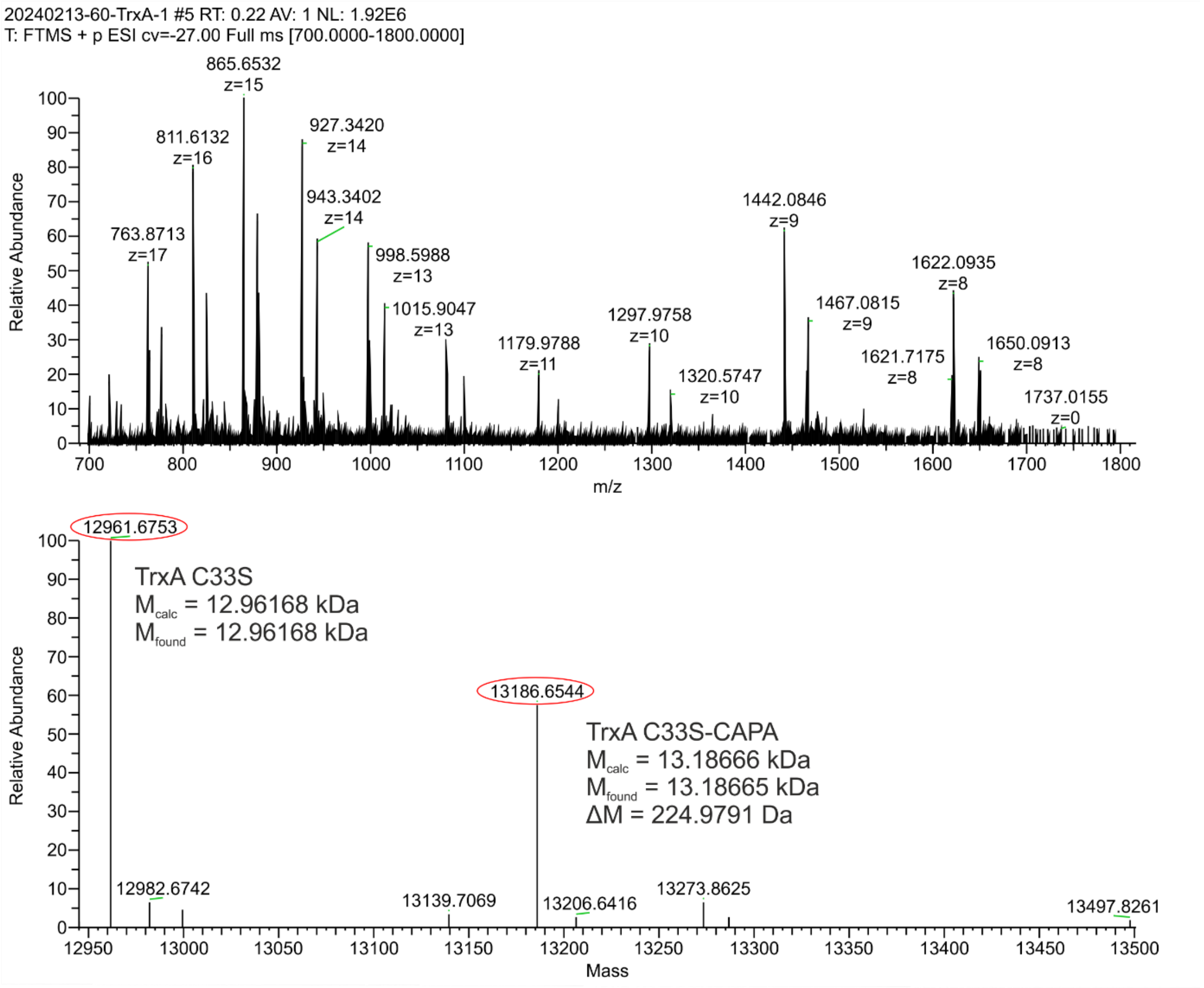

Figure S9: MS spectra of modified protein **TrxA C33S-CAPA** (reaction with TrxA C33S (1 eq) and CAPA (0.5 eq). Measured by direct injection on a Thermo Scientific Oribtrap Eclipse Tribrid via Thermo Scientific HESI-Spray and Thermo Scientific FAIMS interface.

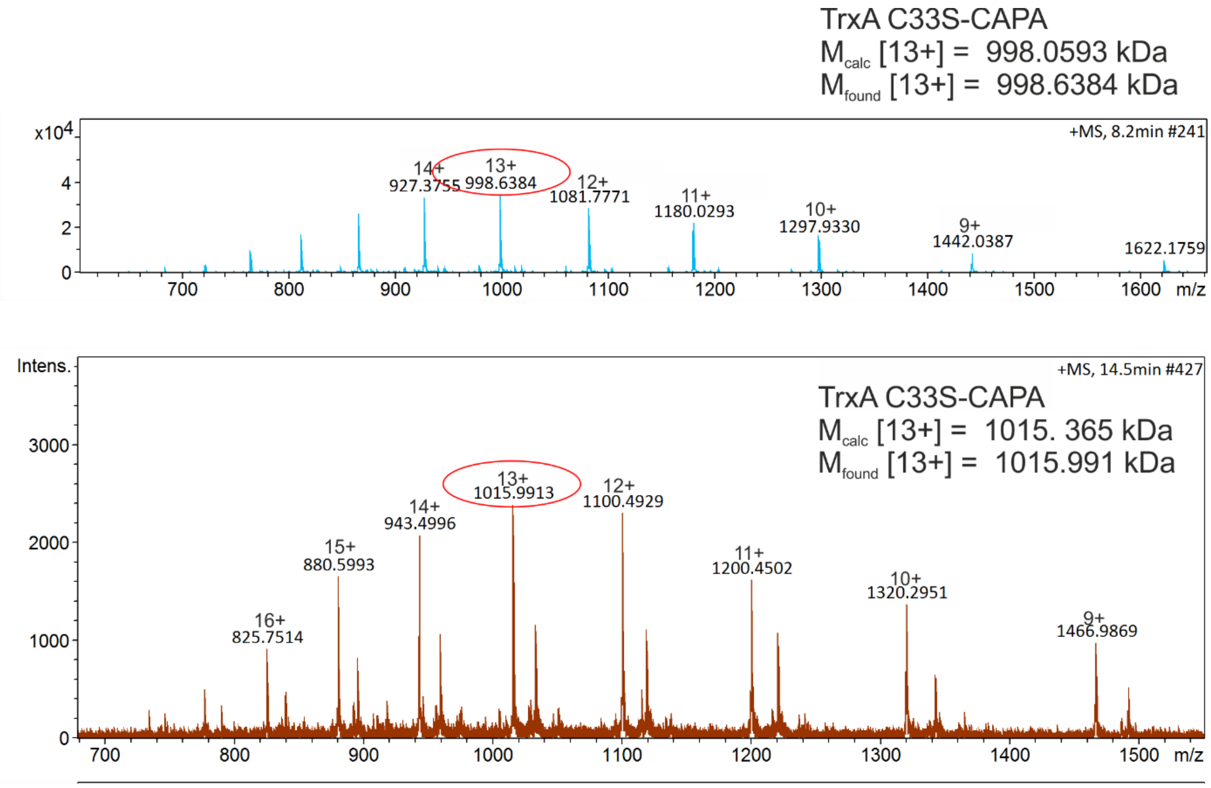

Figure S10: MS spectra of modified protein **TrxA C33S-CAPA** (reaction with TrxA C33S (1 eq) and CAPA (0.5 eq). Measured by LCMS on a rapid separation (RS) Thermo Scientific dionex ultimate 3000 series equipped with a Bruker microTOF II. NOTE: LCMS was not calibrated beforehand.

Bovine Serum Albumin (BSA) Covalent Modification (**BSA-CAPA**) with CAPA (1.0 eq)

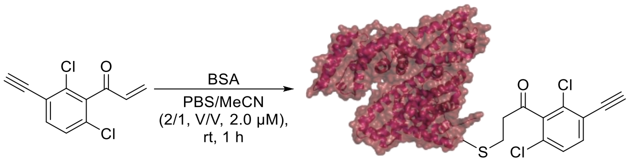

Note: BSA Figure, PDB: 4F5S

To a solution of BSA (25 µL, 2.0 µM in PBS7.4/MeCN; 2/1, V/V, 1.0 eq) was added 1-(2,6-dichloro-3-ethynylphenyl)prop-2-en-1-one (**CAPA**) (2.5 µL, 20 mM in MeCN, 1.0 eq). After 1 h, the reaction was quenched with H_2O containing TFA (0.1 v%) (30 µL) and analyzed by LC-HRMS (Figure S11).

NOTE: While TrxA C33S and its modified version could be detected via intact-protein analysis on the two high-resolution instruments, namely a Thermo Scientific Oribtrap Eclipse Tribrid and a Thermo Scientific dionex ultimate 3000 series equipped with a Bruker microTOF II, the BSA analysis was restricted to the latter instrument.

MS data of CAPA-modified BSA:

LC-HRMS (ESI pos): *m/z*: calcd for $C_{2943}H_{4662}N_{780}O_{899}S_{39}Cl_2^{42+}$ $[M+42H]^{42+}$: 1588.089; found: 1588.106.

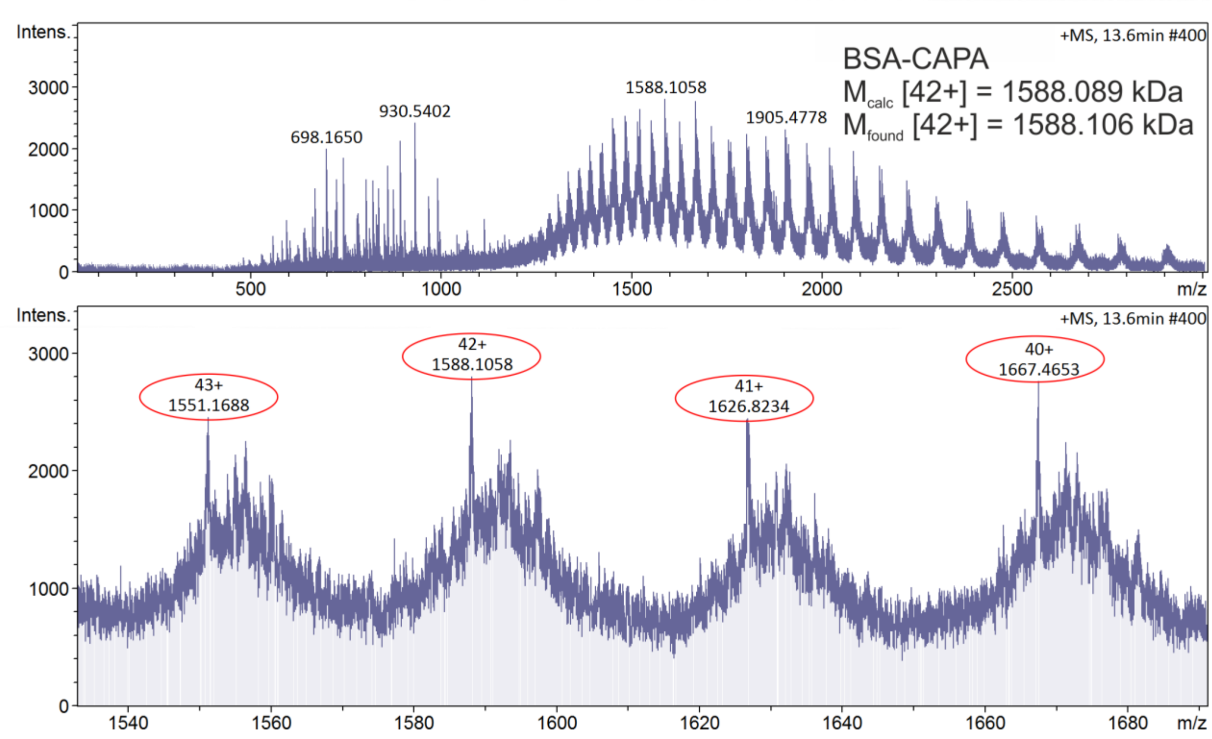

Figure S11: MS spectra of modified protein BSA-CAPA. (reaction with BSA and CAPA: 1/1). Measured by LCMS on a rapid separation (RS) Thermo Scientific dionex ultimate 3000 series equipped with a Bruker microTOF II. NOTE: LCMS was calibrated beforehand.

MS data of unmodified BSA:

LC-HRMS (ESI pos): *m/z*: calcd for $C_{2932}H_{4656}N_{780}O_{898}S_{39}^{42+}$ $[M+42H]^{42+}$: 1582.731; found: 1582.745.

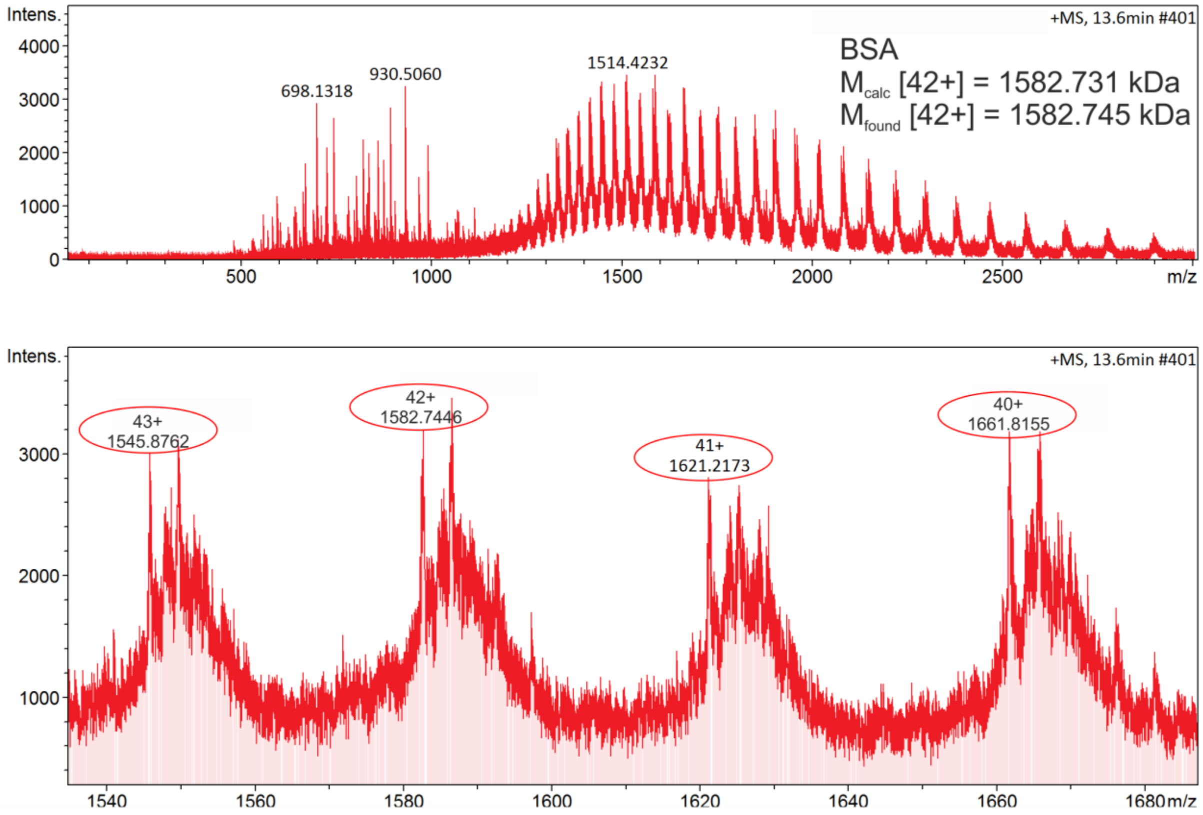

Figure S12: MS spectra of unmodified protein BSA. Measured by LCMS on a rapid separation (RS) Thermo Scientific dionex ultimate 3000 series equipped with a Bruker microTOF II. NOTE: LCMS was calibrated beforehand.

6. Protein expression of ThioredoxinA (TrxA) C33S and purification from E. *coli* cells[9]

Materials

Chemicals for protein expressions and purification were purchased from Merck (Sigma-Aldrich) and Carl Roth, oligonucleotides from Biomers (Germany) and restriction enzymes, Miniprep kit, gel extraction kit, DNA purification kits and T4 DNA ligase from New England Biolabs (Ipswitch, MA).

Methods

TrxA-C33A was cloned, expressed and purified as previously reported.[10]

In brief, the C33A mutant was introduced in the expression plasmid pET28a_His6_TrxA, encoding the His6-tagged wild-type Escherichia coli TrxA (Uniprot No. P0AA25; TrxA C33A – Fwd: GGTCCGGTCCGTGC; TrxA C33A – Rev: ACTCTGCCCAGAAATCGAC)

Protein sequence of the His-tagged E. *coli* TrxA (13 kDa). (red: A33 and C36)

His6-TrxA C33A sequence:

MKHHHHHHPMSDKIIHLTDDSFDTDVLKADGAILVDFWAEWAGPCKMIAPILDEIADEYQGK
LTVAKLNIDQNPGTAPKYGIRGIPTLLLFKNGEVAATKVGALSKGQLKEFLDANLA*

For protein expression, the plasmids encoding for His 6-TrxA C33A was transformed into electrocompetent *E. coli Bl21* (DE3) (Novagen). An overnight culture grown in LB medium supplemented 50 µg/mL kanamycin (Kan), was diluted 1:100 in LB-Kan and incubated at 37 °C, 180 rpm until an OD600 of 0.6 was reached. After induction of protein expression by addition of 0.5 mM isopropyl-β-D-1-thiogalactopyranoside (IPTG), the temperature was reduced to 16 °C and protein expression continued over night. Cells were harvested by centrifugation (6000 g, 20 min) and the cell pellet flash frozen and stored at –80 °C.

Cells from 0.5 L expression culture were resuspended in 40 mL lysis buffer (PBS, Sigma-Aldrich), supplemented with DNase I (AppliChem) and complete mini EDTA-free protease inhibitors (Roche) and lysed by homogenization using an EmulsiFlexC5 (Avestin Inc.). The resulting lysate was cleared by centrifugation (24,446 g, 30 min, 4 °C), subsequently filtered (Whatman TM folded filter, Cytiva) and loaded to nickel-nitrilotriacetic acid (NTA) agarose resin (Qiagen) equlibrated with lysis buffer. The resin was washed with PBS, 20 mM imidazole and the protein was eluted with PBS, 500 mM imidazole. The imidazole concentration was reduced by dilution, the protein concentrated using centrifugal filter devices (Amicon Ultracell Centrifugal filter unit MWCO 3 kDa, Merck Millipore) and loaded onto a Superdex 200 increase (10/300) GL column (Cytiva), equilibrated with PBS. Fractions containing the pure protein were concentrated, aliquoted, flash-frozen in liquid nitrogen and stored at −80 °C.

7. Chemoproteomics Experiments

7.1 Cell culture

Cells were grown in RPMI 1640 medium (Gibco) with 10% FBS (Biochrome, Cat.no., S0115, Lot No., 0868B and 50 U/mL penicillin-streptomycin antibiotic (Biowest) and maintained at 37 °C under 5% CO_2 atmosphere.

7.2 Proteome Preparation

Protein lysates were prepared from 90% confluent 15 cm dishes that were put on ice, washed twice with ice-cold PBS (Sigma-Aldrich) and collected by scraping in 1 mL of ice-cold PBS. Cells were pelleted by centrifugation (5 min at 4 °C, 400 g), snap-frozen on dry ice and keep at –80 °C until further processing. To lyse the cells, the pellet was diluted in 300 µL ice-cold PBS with 1x protease inhibitor (Roche) and sonicated with a rod sonicator 4 × 10 pulses (1 s on/off) at 20% amplitude and 2 × 10 pulses at 40% amplitude (Fisher Scientific, model: Fisherbrand™ Q125 Sonicator with microprobe) until the lysate was homogenous. The lysate was then separated into soluble and particulate fraction through centrifugation for 30 min at 21,000 g, 4 °C. Protein concentration was determined with the DC assay (Bio-Rad).

7.3 In-gel ABPP Experiments

The experiment was performed in 96-well plates by adding 100 µL of cell lysate (1 mg/mL) followed by the addition of the chemical probe-of-interest at the desired concentration. The samples were incubated for 1 h at rt in the dark. Next, a "click" master mix for all samples (6 µL 1.7 mM TBTA in *t*BuOH/DMSO (4/1, v/v), 2 µL 50 mM $CuSO_4$ in H_2O, 2 µL 1.25 mM TAMRA-azide (**S25**) (Sigma-Aldrich) in DMSO and 2 µL freshly prepared 50 mM TCEP in H_2O per sample) was prepared and subsequently added to each sample well followed by a 1 h incubation at rt in the dark under shaking. The reaction was quenched with 34 µL of 5x laemmli buffer (0.25 M Tris pH 6.8, 25% glycerol, 10% sodium dodecyl sulfate, 0.5% Bromophenol blue, 0.1 M dithiothreitol added fresh). Samples were frozen at –20 °C until further processing. Proteins were separated on 12.5% SDS-PAGE gels according to a standard protocol and fluorescence was detected using the ChemiDoc MP (Bio-Rad). To visualize total protein load, the gels were stained with Coomassie brilliant blue R 250 (Fisher Bioreagents).

7.4 ABPP Coupled to LC-MS/MS

Treatment of live cells:

Live cell probe incubation and cell lysis

Cells were grown until 90% confluence on 10 cm dishes and treated with 200 µM CAPA in serum-free medium (10 µL CAPA from a 100 mM DMSO stock diluted with 5 mL serum-free medium) for 1 h at 37 °C per plate. The plates were put on ice, the media was removed, washed 2 × with ice-cold PBS (Sigma-Aldrich) and harvested by scraping in 1 mL of ice-cold PBS. The cells were pelleted by centrifugation for 5 min at 500 g at 4 °C. To lyse the cells, the pellet was diluted in 200 µL complete PBS (ice-cold PBS with 1x cOmplete™, Mini, EDTA-free protease inhibitor (Roche)) and sonicated with a rod sonicator 8 × 10 pulses (1 s on/off) at 40% amplitude (Fisher Scientific, model: Fisherbrand™ Q125 Sonicator with microprobe) until the lysate was homogenous. The lysate was centrifuged 30 min at 21,000 g, 4 °C and the supernatant was collected into a protein low binding microcentrifuge tube.. Protein concentration was determined with the BCA assay (Pierce) and normalized to 1.86 mg/mL with complete PBS. The protocol was continued with the reduction step (see Reduction (only for CAPA bound lysate))

Treatment of cell lysates:

Cell lysis

Cells were grown until 90% confluence, washed 2 × with ice-cold PBS, harvested by scraping and centrifuged 5 min at 500 g at 4 °C. The supernatant was removed, and the cell pellet was quick-frozen for 15 min on dry ice until further processed. The cell pellet from a 1 × 15 cm dish was resuspended in 500 µL complete PBS (PBS with 1x cOmplete™, Mini, EDTA-free Protease Inhibitor Cocktail (Roche)) and sonicated with a rod sonicator (Fisher Scientific, model: Fisherbrand™ Q125 Sonicator with microprobe) 8 cycles (10 s on/off) on 40% amplitude. The cell lysate was centrifuged 30 min at 21,000 g, 4 °C and the supernatant was collected into a protein low binding microcentrifuge tube. Concentration was measured with the BCA assay (Pierce) and normalized to 2 mg/mL with complete PBS.

Probe incubation

1 µL of 20 mM probe (CAPA (**11**) or IAA (**1**)) stock solution in DMSO was added to 100 µL cell lysate (200 µg) per heavy and light probe and incubated for 1 h at 37 °C while shaking (1000 rpm) in the dark.

Reduction (only for CAPA bound lysate)

The pH of the sample was adjusted by adding 1.6 µL aq. NaOH (0.1 M) followed by a reduction step where 20 µL of aq. $NaBH_4$ (2 M) was added to the sample. The sample was incubated 30 min on ice and 15 min at 25 °C while shaking (1000 rpm). The reaction was quenched with 3 µL acidic acid and the pH was neutralized by adding 28 µL of aq. NaOH (0.1 M).

Note: As part of the optimization of the protocol, the pH was tested using pH strips (Machery-Nagel, cat.no., 92122) and the final pH of the sample prior to the SP3 clean-up ranged between 7.5–8.0.

Coupling to isoDTB (**19**) by copper-catalyzed azide-alkyne cycloaddition (CuAAC)

A master "click" mix was prepared by mixing 6 µL of 1.7 mM TBTA in *t*BuOH/DMSO (4/1, V/V, final concentration: 100 µM), 2 µL of 50 mM $CuSO_4$ (final concentration: 1 mM), 2 µL of 150 mM TCEP (final concentration: 3 mM), 4 µL of 5 mM light or heavy isoDTB (**19**) (final concentration: 200 µM) and 2 µL 10% SDS per sample. From the master mix, 16 µL was added to each sample and incubated for 1 h at 37 °C while shaking (1000 rpm). After incubation, 0.1 µL of universal nuclease for cell lysis (Pierce) was added to each sample and incubated for 30 min in 37 °C. After this step heavy and light samples was combined into one sample.

SP3 clean-up

Carboxylate-modified E7 and E3 magnetic SpeedBeads solution (Cytiva) was mixed 1/1 in a total of 40 µL for each sample (one sample is now heavy and light combined). The magnetic beads were washed 3 × 1 mL with LC-MS grade water (Carl Roth) by resuspending the beads in water and using a magnetic rack to remove the water without disrupting the beads. For each sample, 40 µL beads were added to 1 mL absolut ethanol followed by the addition of the protein sample. The samples were incubated for 5 min at rt while shaking (1000 rpm). The tubes were put on the magnetic rack and the supernatant was removed. The beads were washed 3 × 400 µL 80% ethanol and resuspended in 200 µL pre-trypsin digestion buffer (prepared fresh: 2 M urea in PBS with 0.5% SDS). DTT was added to a final conc. of 10 mM by adding 10 µL of a 200 mM stock and the sample was incubated for 15 min at 65 °C. Iodoacetamide was added to a final conc. of 20 mM by adding 10 µL of a 400 mM stock and the sample was incubated for 30 min at 37 °C. 800 µL of absolute ethanol was added and the sample was incubated for 5 min, rt, at 1000 rpm. The supernatant was discarded. The beads were washed 3 × 400 µL with 80% ethanol and resuspended in trypsin digestion buffer (prepared fresh: 2 M urea in PBS)). 2 µL of 0.5 mg/mL trypsin (Pierce) was added and the samples were incubated at 37 °C overnight while shaking (200 rpm).

SP3 elution

The supernatant of the digest was collected, and the beads were resuspended with 50 µL SP3 elution buffer (2% DMSO in H_2O) and incubated at 37 °C for 30 min while shaking (1000 rpm). The supernatant was collected into the same vial and the elution was repeated a second time.

Streptavidin enrichment
For each sample, 50 µL of high-capacity streptavidin bead slurry was washed 3 × 1 mL PBS by centrifugation for 1 min at 1400 g. Each 50 µL bead slurry was resuspended in 300 µL PBS, added to the SP3-eluted sample and rotated for 2 h at rt. The beads were centrifuged for 2 min at 1400 g and the supernatant was discarded. The beads were transferred to bio-spin columns (Pierce) and washed 3 × 600 µL PBS followed by 3 × 600 µL H_2O and 3 × 600 µL 50% MeCN. The column was transferred to a fresh microcentrifuge tube and the peptides were eluted by adding 200 µL of 0.1% TFA in 50% MeCN and let it slowly drip through. Two more rounds of elution were performed by adding 70 µL of the same organic phase. The peptides were eluted completely by centrifugation at 2500 g for 3 min. The sample was dried via speed vac and the peptide pellet was resuspended in 30 µL 0.1% TFA in water followed by 3 min in a sonication bath. A 0.22 µm centrifugal filter (Merck) was washed with 300 µL 0.1% TFA in water and centrifuged at 17,000 g. The filter was placed in the supplied tube and the sample was added to the filter and centrifuged for 2 min at 17,000 g. The filtered sample was transferred to MS sample vial. 5 µL of sample was injected into the LC-MS.

7.5 LC-MS/MS Measurement and Data Processing

LC-MS/MS method
MS measurements were performed on an Orbitrap Eclipse Tribrid Mass Spectrometer (Thermo Fisher Scientific) coupled to an UltiMate 3000 Nano-HPLC (Thermo Fisher Scientific) via a Nanospray Flex (Thermo Fisher Scientific) and FAIMS interface (Thermo Fisher Scientific). First, peptides were loaded on an Acclaim PepMap 100 µ-precolumn cartridge (5 µm, 100 Å; 300 µM ID x 5 mm, Thermo Fisher Scientific). Then, peptides were separated at 40 °C on a PicoTip emitter (noncoated, 15 cm, 75 µm ID, 8 µm tip, New Objective) that was packed in house with ReprosilPur 120 C18-AQ material (1.9 µm, 150 Å, Dr. A. Maisch GmbH). The LC buffers consisted of MS-grade water (A) and acetonitrile (B) both supplemented with 0.1% formic acid. The gradient was run from 4.00-35.2% B during a 150 min method (0-5 min 4%, 5-6 min to 7%, 7-105 min to 24.8%, 105-126 min to 35.2%, 126-140 min 80%, 140-150 min 4%) at a flow rate of 300 nL/min.

Data-dependent acquisition
FAIMS was performed with two alternating compensation voltages (CVs) including –50 V and –70 V. The mass spectrometer was operated in dd-MS2 mode with following settings: Polarity: positive; MS1 Orbitrap resolution: 240k; MS1 AGC target: standard; MS1 maximum injection tme: 50 ms; MS1 scan range: m/z 375-1500; RF Lens: 30%; MS2 Orbitrap resolution: 15k; MS2 AGC target: standard; MS2 maximum injection time: 35; HCD collision energy: 30%; RF Lens: 30%; MS2 cycle time: 1.7 s; intensity threshold: 1.0e4 counts; included charge states: 2-6; dynamic exclusion: 60 s.

Data analyzation of acquired mass spectra

Prior to fragpipe analysis *.raw files were converted to *.mzML format with "MSConvert" from the "ProteoWizard" software package. The additional filters "peakPicking" and "zeroSamples" were added with their default settings. Peptides were searched in Fragpipe version 22.0 with MSFragger 4.1. IonQuant 1.10.27, diaTracer 1.1.5, DIA-NN 1.8.2 and Python 3.9.10 including EasyPQP 0.1.52. A FASTA database was downloaded from www.uniport.org using a search for "UP000005640" in Proteomes on 28.02.2025 which was supplemented with a list of universal contaminants.[11] The FASTA file was loaded into Fragpipe 22.0 and reverse sequences was added by "Add decoys". Offset search: All samples were run with the "Experiment" column left empty. To analyse the amino acid selectivity, an Offset Search was performed in MSFragger 4.1. For this purpose, the following settings were used in MSFragger: Precursor mass tolerance −20 to 20 ppm, fragment mass tolerance 20 ppm, Calibration and Optimization "Mass calibration, parameter optimization", Isotope Error "0/1/2", enzyme name "trypsin", cut after "KR", but not before "P", cleavage "enzymatic", missed cleavages "2", Clip N-term M enabled, peptide length 6 to 50, peptide mass range 500 to 5000 Da, variable modification of 57.02146

Da on C with max. 3 occurrences, no fixed modifications, mass offsets set to 0.0/707.2713/713.2788, all other options were left at the standard settings. Crystal-C and run MSBooster were disabled. PeptideProphet was run with the following settings: "--nonparam --expectscore --decoyprobs --masswidth 1000.0 -- clevel -2". PTMProphet was disabled. ProteinProphet was run with the following settings: "--maxppmdiff 2000000". Generate report was enabled with the following settings: "--sequential --mapmods --prot 0.01", generate protein-level summary was enabled and all the other options were disabled. All other tools were disabled.

For downstream data analysis, the "psm.tsv" file, which contains the PSMs filtered by 1% PSM- and protein-level FDR, was used. For each sample, only the spectra that belong to that experiment were isolated. The column "MSFragger Localization" indicates the possible residues modified by the mass offset. The residues with the highest hyperscore are indicated by lower-case letters. Only entries were retained that were localized to a unique residue as seen by containing one lower-case letter Next, the entries were filtered for a delta score > 1, where delta score is the between "Score Best Position" and "Score Second Best Position". For each entry, the UniProt Code was isolated from the column "Protein" and the full protein sequence was linked into the table from the FASTA database. The residue number of the modified residue was determined. Next, the amino acid at the modified residue was determined. If the *N*-terminus (modification at amino acid 1 or amino acid 2 if amino acid 1 is not present in the peptide (clipping of *N*-terminal methionine)) or the *C*-terminus (last amino acid of the protein) were modified, this was only counted and labelled as modification of the terminus and not of the respective amino acid at that position. For each entry, an identifier was generated in the format "UniProtCode"_X_"residue number", where X is the one letter code of the modified amino acid or "*N*-terminal" or "*C*-terminal" for terminal modifications. Duplicates of entries with the same identifier were retained only once. The data of all experiments was then combined and only residues were counted in the final analysis that were present in 2 out of 3 replicates. The fraction of all sites that was modified at each amino acid and the termini was reported.

Closed Search
All samples were run with information added to "Experiment". "Bioreplicate" column was left empty. To quantify specific amino acids, a Closed Search was performed in MSFragger. For this purpose, the following settings were used in MSFragger: Precursor mass tolerance −20 to 20 ppm, fragment mass tolerance 20 ppm, Calibration and Optimization "Mass calibration, parameter optimization", Isotope Error "0/1/2", enzyme name "trypsin", cut after "KR", but not before "P", cleavage "enzymatic", missed cleavages "2", Clip N-term M enabled, peptide length 6 to 50, peptide mass range 500 to 5000 Da, no mass offsets, in advanced options under spectral processing the " require precursor" was disabled. All other options were left at the standard settings. Max variable mods on a peptide were set to 3 with max combinations of 5000. "Use all mods in first search" was enabled. Variable modifications were set on C to 707.2713 and 713.2788 for CAPA, and 576.3133as well as 582.3208 for IAA with max. 1 occurrence. Further variable modifications with oxidation of methionine (15.9949) with maximum 2 occurrences, acetylation of the N-terminus (42.0106) with maximum 1 occurrence and carbamidomethylation of C (57.0215) with maximum 3 occurrences.. No fixed modifications were set. Crystal-C was disabled. Run MSBooster was enabled and Predict RT as well as Predict spectra was enabled with DIA-NN as model. Run PSM Validation was enabled with the following settings " --decoyprobs --ppm --accmass --nonparam –expectscore". PeptideProphet was disabled. Percolator was run with the following settings --only-psms --no-terminate --post-processing-tdc with the Min probability set to 0.5. PTMProphet was disabled. ProteinProphet was run with the following settings: "--maxppmdiff 2000000". Generate report was enabled with the following settings: "--sequential --prot 0.01". Run MS1 quant was enabled with the following settings: IonQuant was enabled LFQ was disabled and Add MaxLFQ enabled with MaxLFQ set to 1. Labelling based quant with the masses for the respective labeling indicated above was added to the heavy and light labeling indicated with a *., re-quantify enabled, match between runs were enabled and normalize intensity across runs was disabled., MBR ion FDR was set to 0.01, peptide-protein uniqueness was set to "unique+razor", min scans was set to 1, Min isotopes was set to 2, m/z tolerance was set to 5 ppm, RT tolerance was set to 0.1 minutes

and IM tolerance was set to 0.01 1/k0. MBR RT tolerance was set to 1 minute, MBR IM tolerance (0.1 1/k0) was set to 0.05 and MBR top runs was set to 10. MBR peptide and protein FDR was set to 1. Top N ions and min freq was set to “0”. Min site localization probability was set to 0.75 and “keep index on disk” was disabled. RT window was set to 0.4 min and m/z window to 10 ppm. All other tools were disabled.

For downstream data analysis, the “ion_label_quant.tsv” files of the three experiments were analysed separately. For each entry, the “Modified peptide” was generated as either the “Light Modified Peptide” or the “Heavy Modified Peptide” based on the entry with the higher “Intensity”. The masses of probe modification in the “Modified Peptide” were replaced by an “*” and the masses of carbamidomethylation, oxidation and acetylation in this entry were deleted, if present. The full protein sequence was linked into the table from the FASTA database. Based on this information, for all peptide sequences that do not occur exactly once in the protein, the first occurrence was reported while a label was added for subsequent occurances and the residue number of the modified residue was determined. The “Identifier” was generated in the format “UniProtCode”_X_”residue number”, where X is the one letter code of the modified amino acid or “N-term” or “C-term” for terminal modifications. For each “Identifier”, the median “Log2 ratio HL” was determined as median of the “Log2 ratio HL” of all corresponding ions. If several different “Modified peptides” were detected for the same “Identifier”, the “Modified Peptide” and “Peptide Sequence” with the shortest sequences were kept. For all identifiers, the data for both replicates was now combined. If different “Modified peptides” were detected for the same “Identifier” in the different replicates, the “Modified Peptide” and “Peptide Sequence” with the shortest sequence were kept. The average of the “Log2 Ratio HL” values was calculated and named “Log2 ratio HL”. The value was disregarded, if the identifier was quantified in less than 2 of the replicates or if the standard deviation was >1. Number and overlap of quantified residues and individual proteins were compared between IAA and CAPA and are displayed as a Venn diagram.

8. NMR Spectra

^{1}H NMR (400 MHz, $CDCl_3$) and **^{13}C NMR** (101 MHz, $CDCl_3$)

N-Cbz-L-Cys-OMe (**S1**)

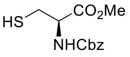

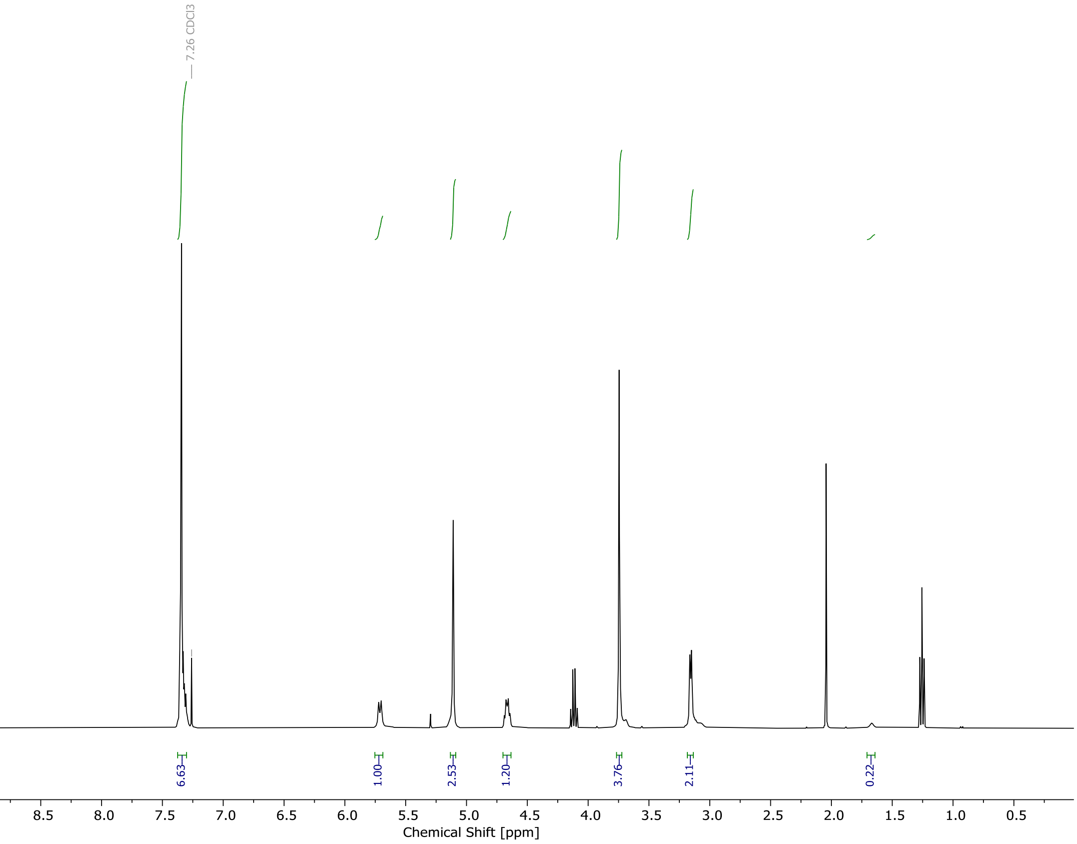

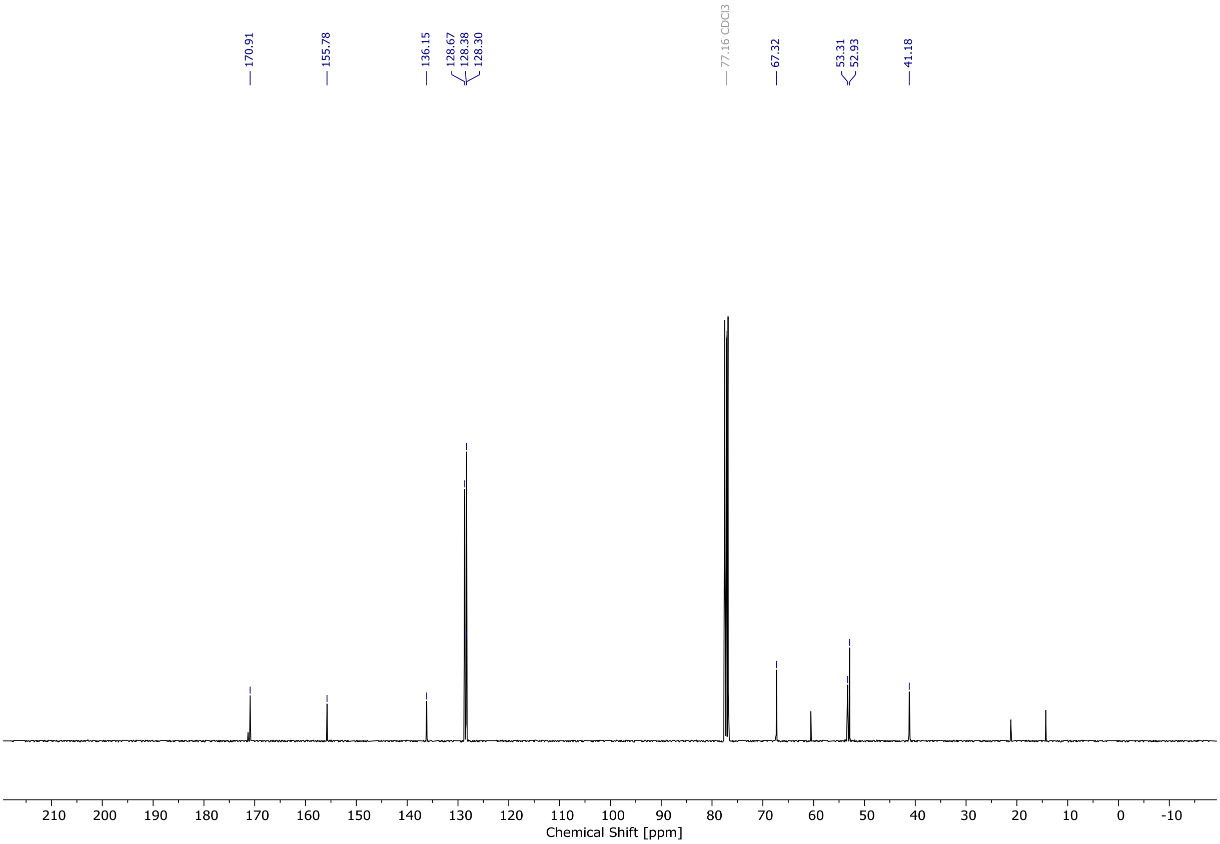

¹H NMR (400 MHz, CD_3OD)

N-Cbz-L-Cys-OMe (**S3**)

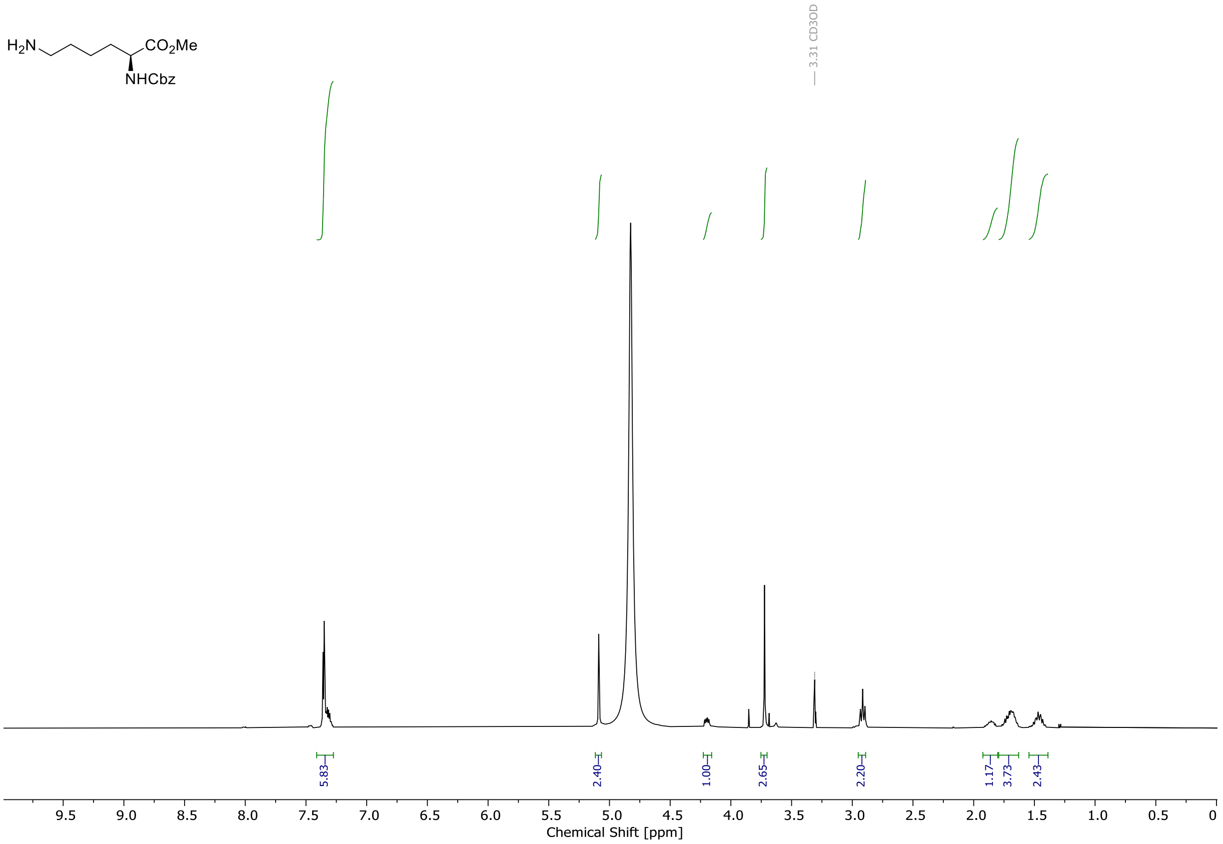

^{1}H NMR (300 MHz, $CDCl_3$) and **^{13}C NMR** (100 MHz, D_2O)

Hex-5-yn-1-amine (**S5**)

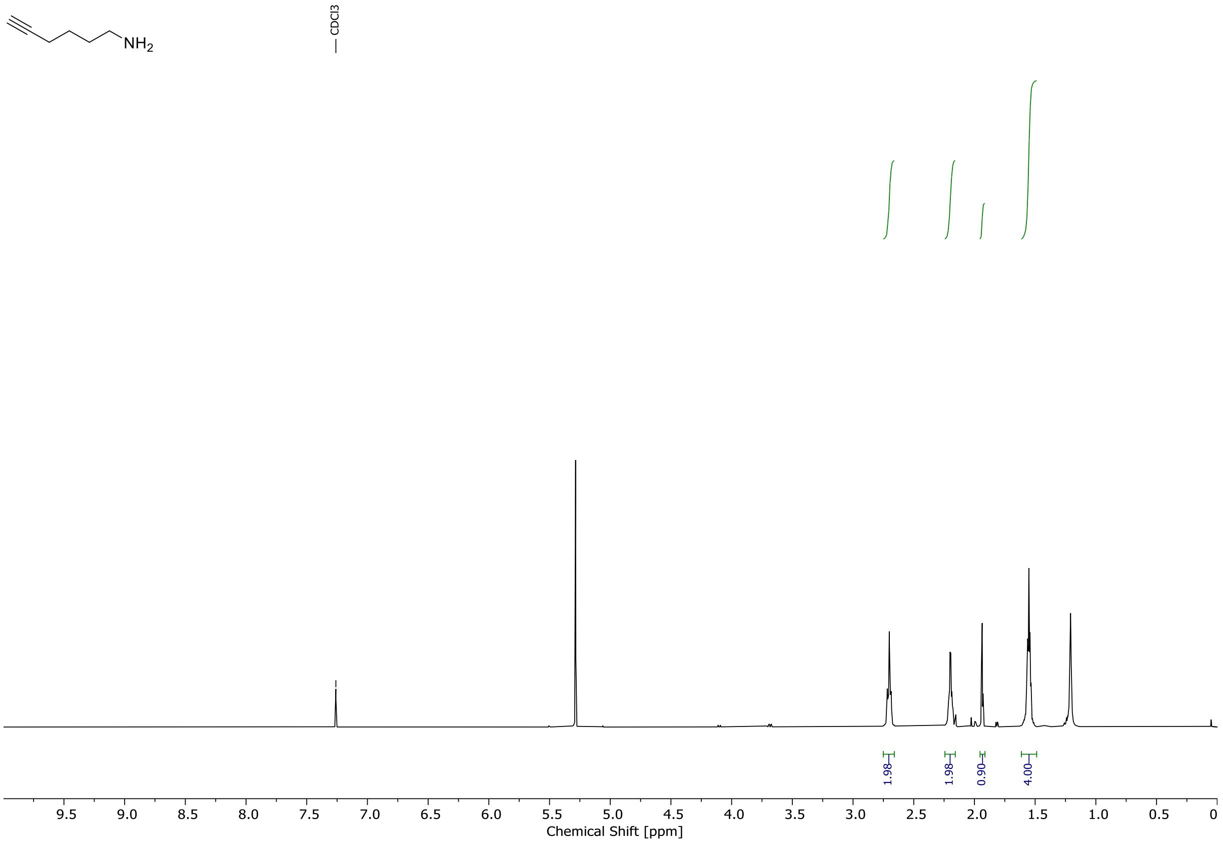

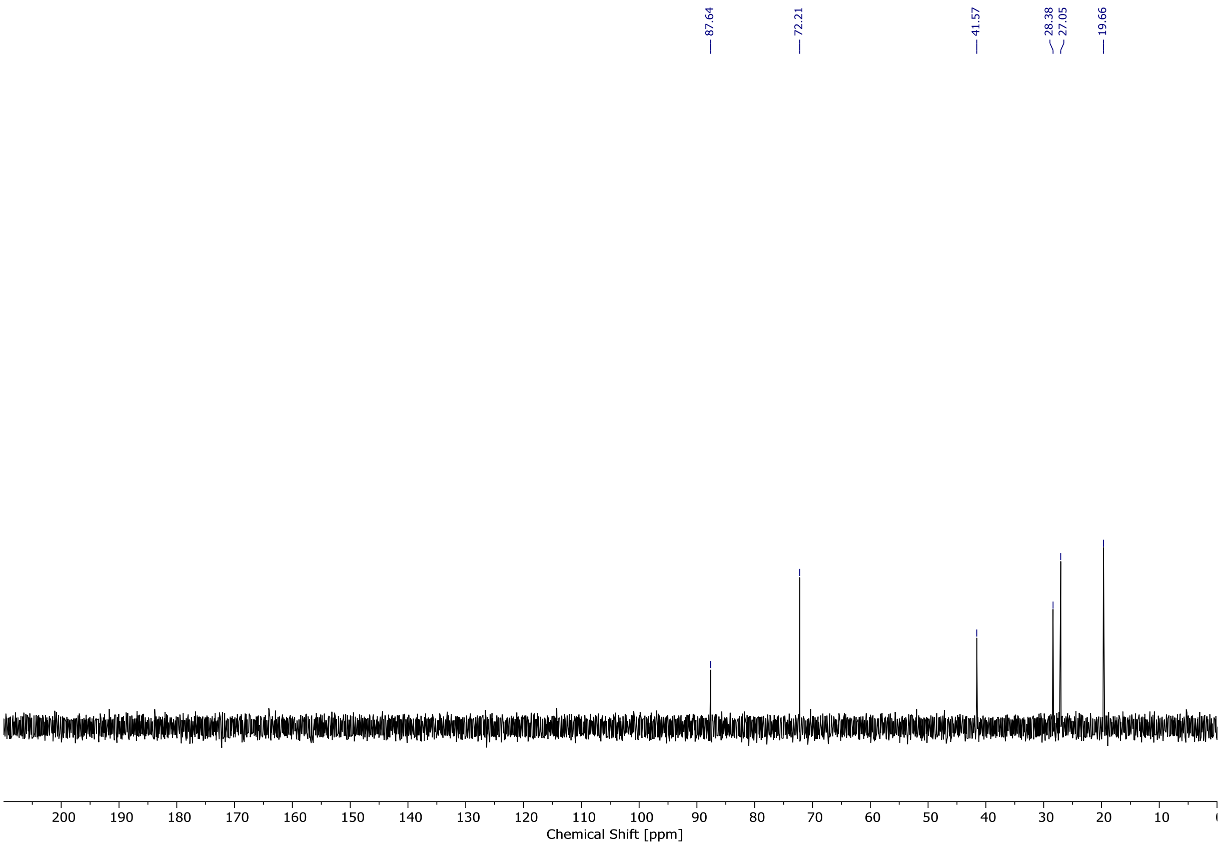

^{1}H NMR (300 MHz, $CDCl_3$) and **^{13}C NMR** (100 MHz, $CDCl_3$)

2-Bromo-*N*-(hex-5-yn-1-yl)acetamide (**S7**)

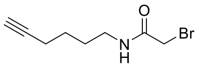

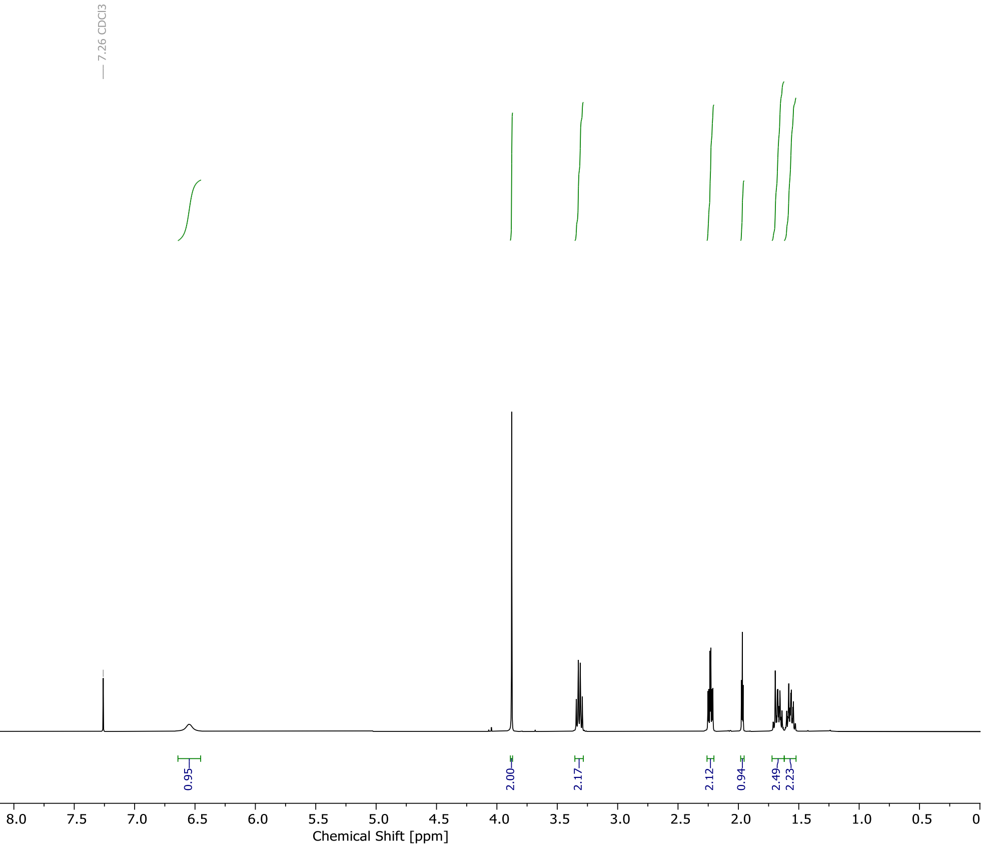

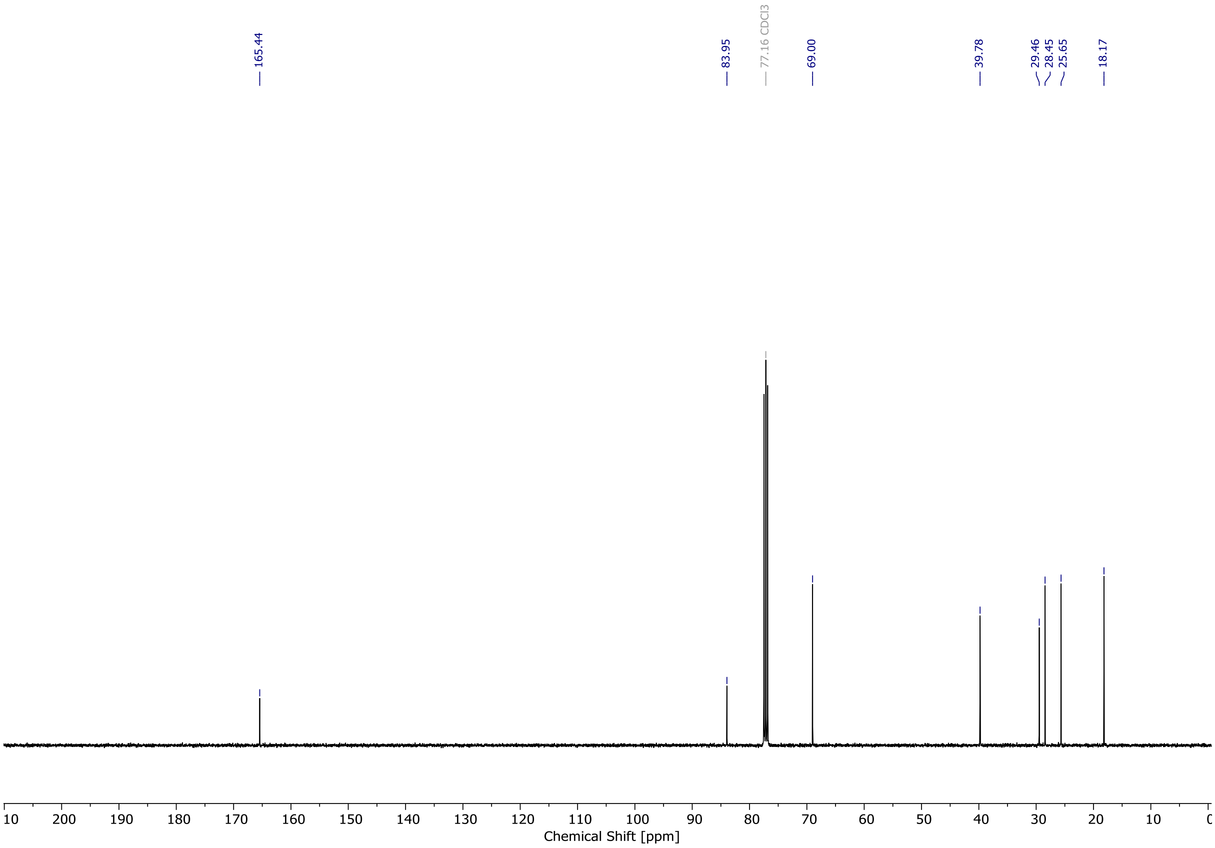

^{1}H NMR (400 MHz, $CDCl_3$) and **^{13}C NMR** (101 MHz, $CDCl_3$)

2-Iodo-*N*-(hex-5-yn-1-yl)acetamide (IAA, **1**)

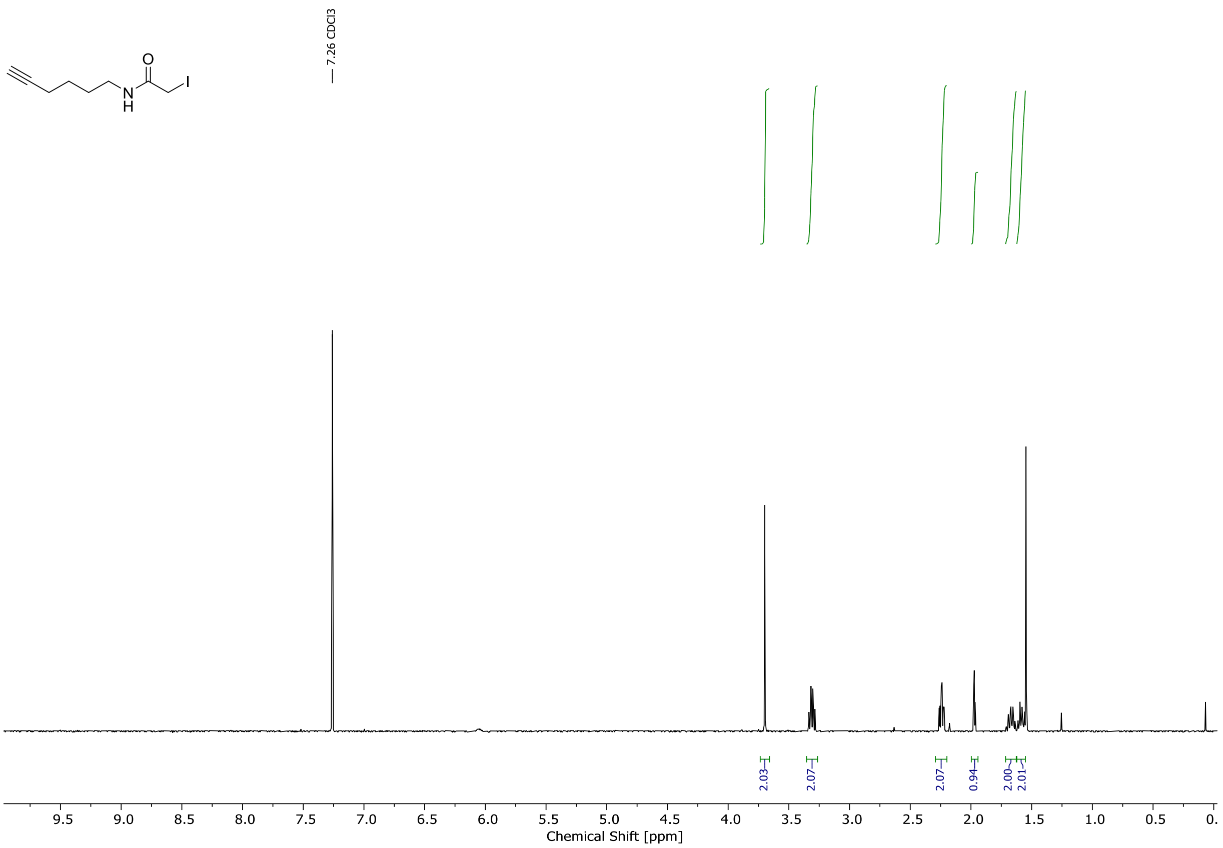

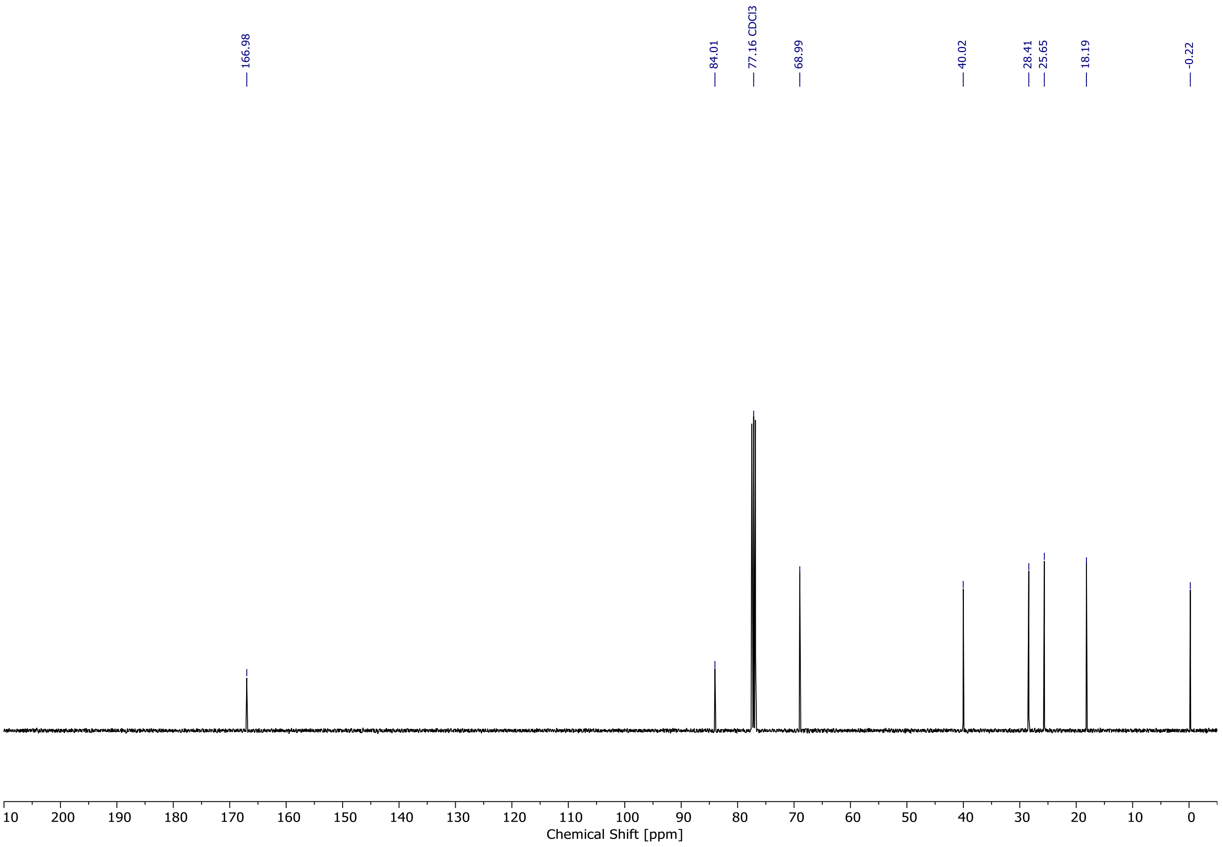

^{1}H NMR (400 MHz, $CDCl_3$) and **^{13}C NMR** (101 MHz, $CDCl_3$)

Methyl *N*-((Benzyloxy)carbonyl)-*S*-(2-(hex-5-yn-1-ylamino)-2-oxoethyl)-L-cysteinate (Cys-IAA, **12**)

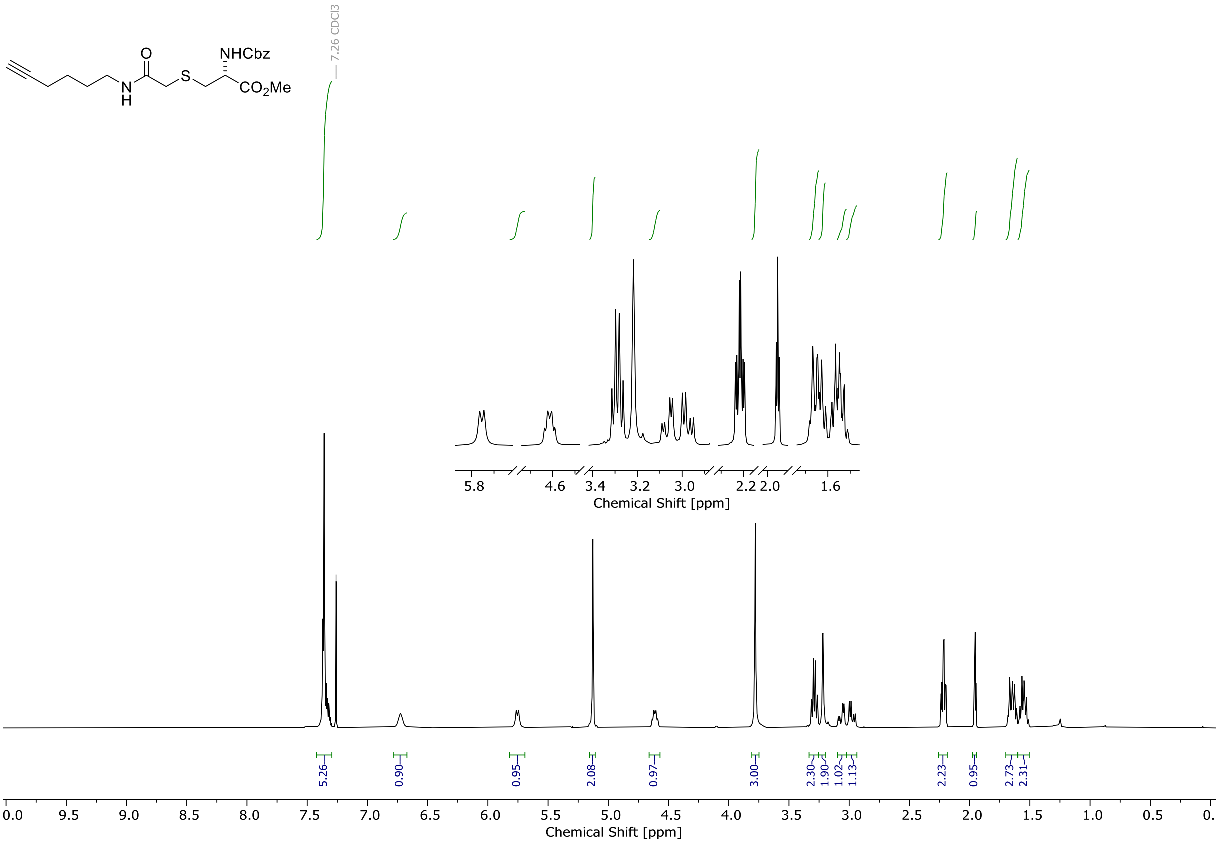

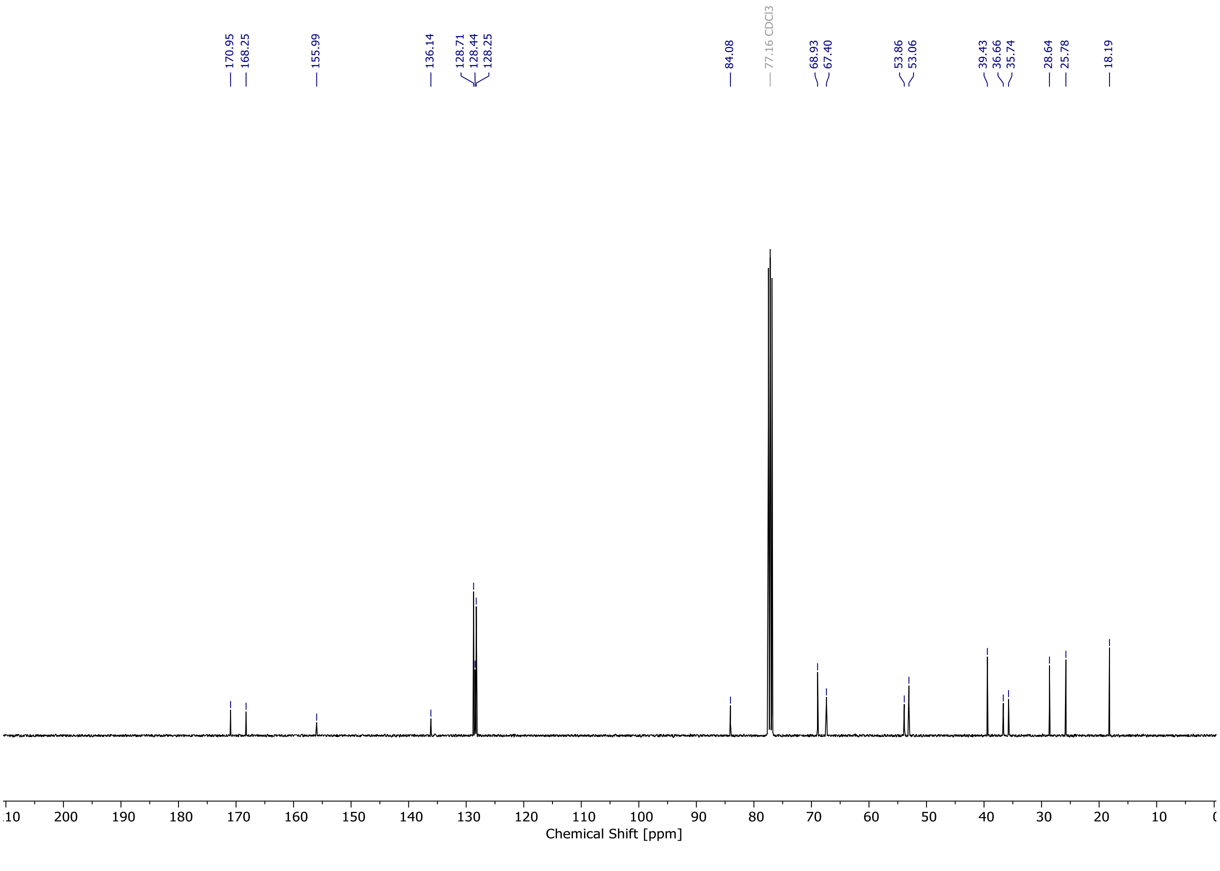

^{1}H NMR (400 MHz, $CDCl_3$) and **^{13}C NMR** (101 MHz, $CDCl_3$)

3-((Trimethylsilyl)ethynyl)benzaldehyde (**S8**)

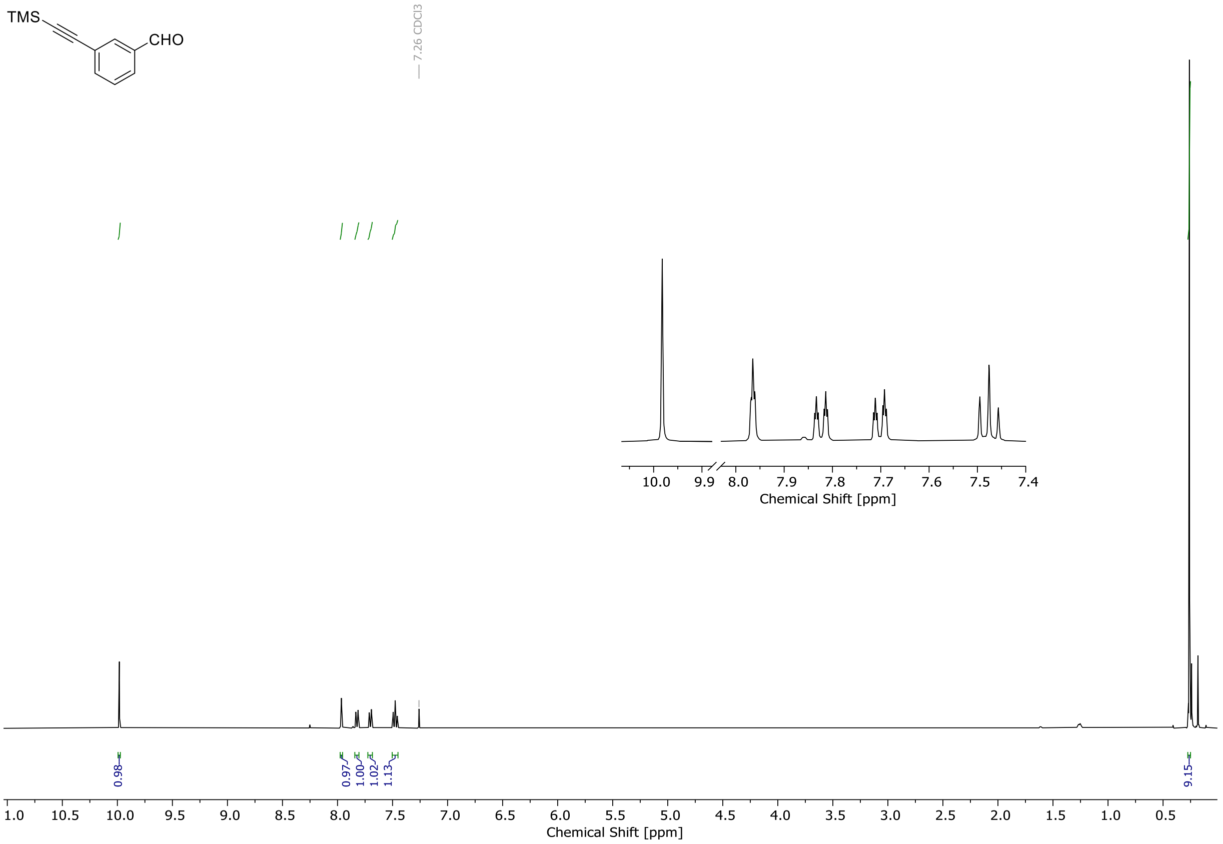

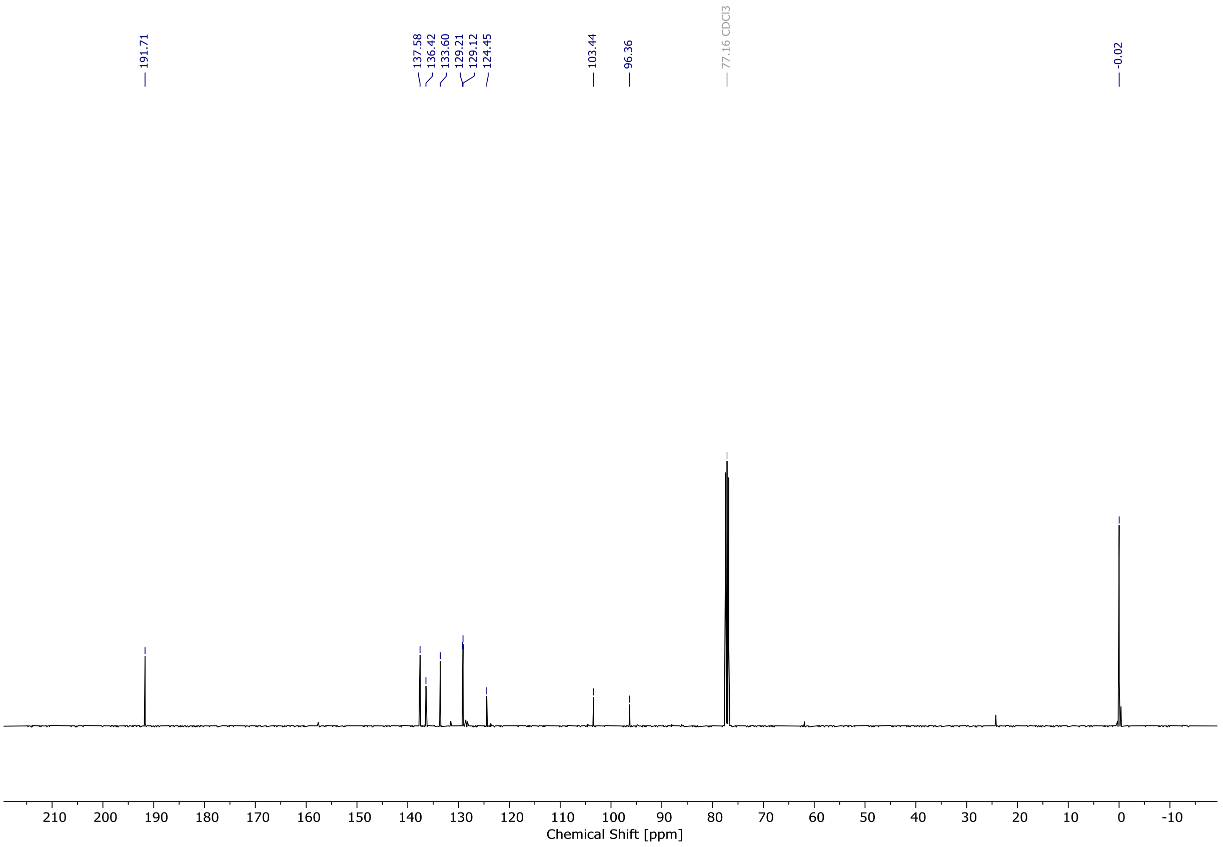

^{1}H NMR (400 MHz, $CDCl_3$) and **^{13}C NMR** (101 MHz, $CDCl_3$)

1-(3-((Trimethylsilyl)ethynyl)phenyl)prop-2-en-1-ol (**S10**)

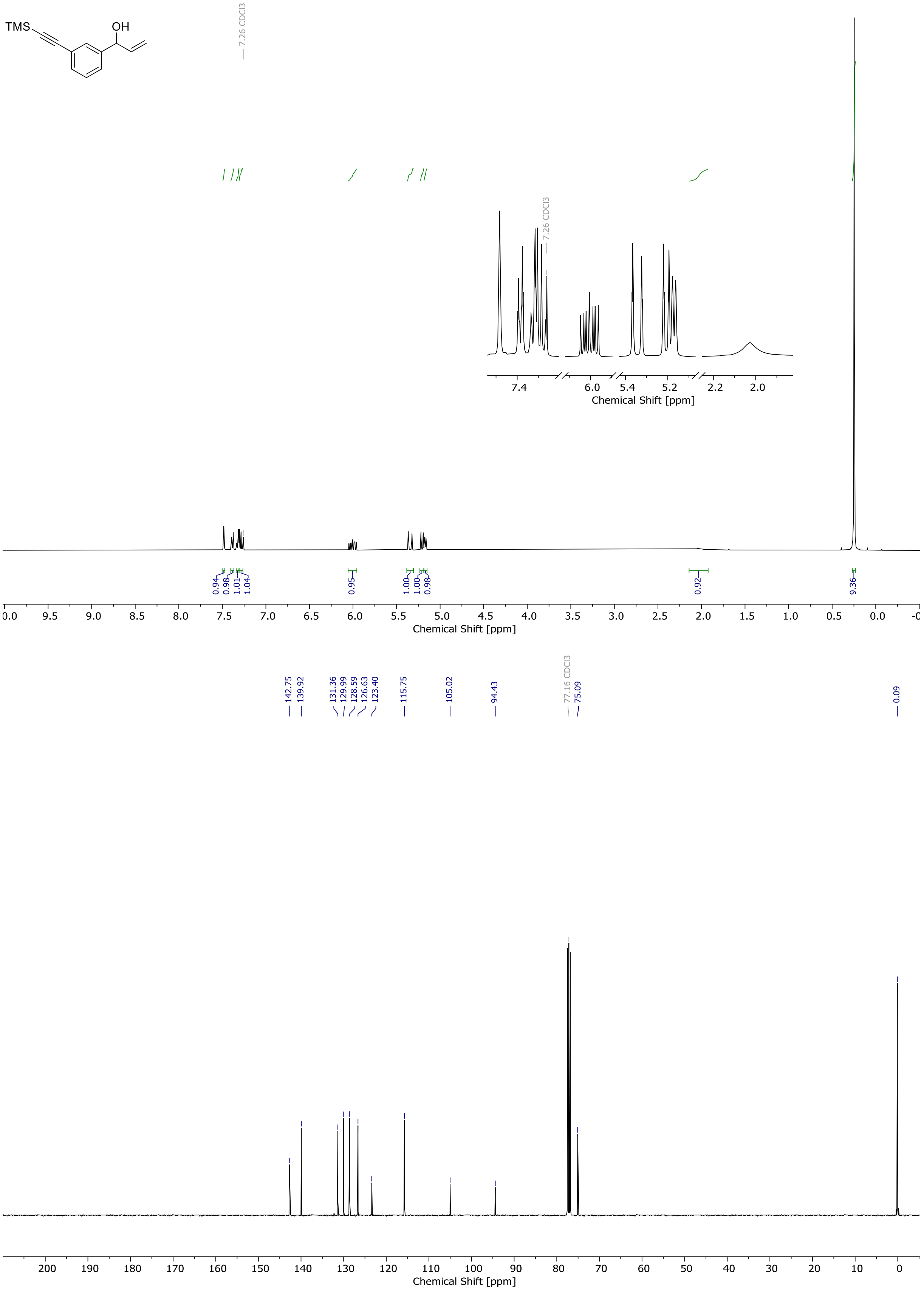

^{1}H NMR (400 MHz, $CDCl_3$) and **^{13}C NMR** (101 MHz, $CDCl_3$)

1-(3-((Trimethylsilyl)ethynyl)phenyl)prop-2-en-1-one (**S11**)

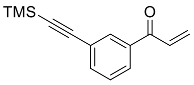

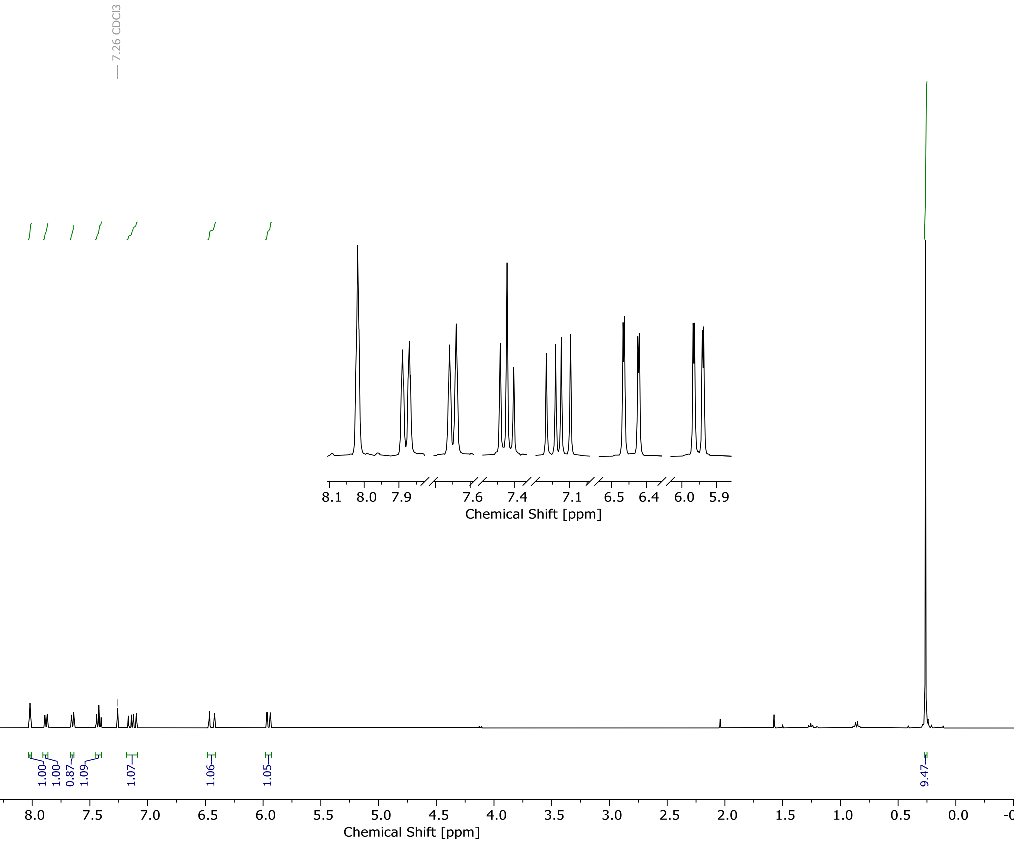

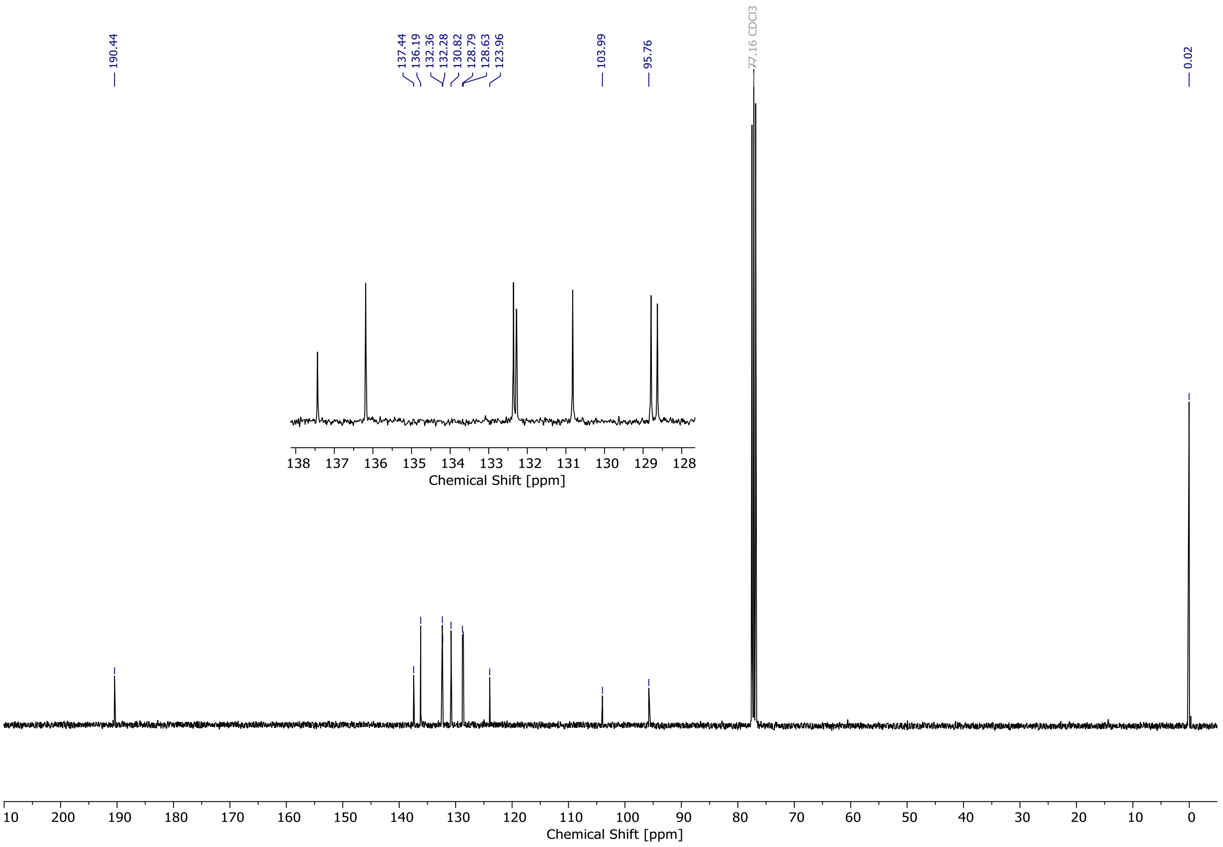

^{1}H NMR (400 MHz, $CDCl_3$) and **^{13}C NMR** (101 MHz, $CDCl_3$)

1-(3-Ethynylphenyl)prop-2-en-1-one (APA, **10**)

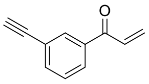

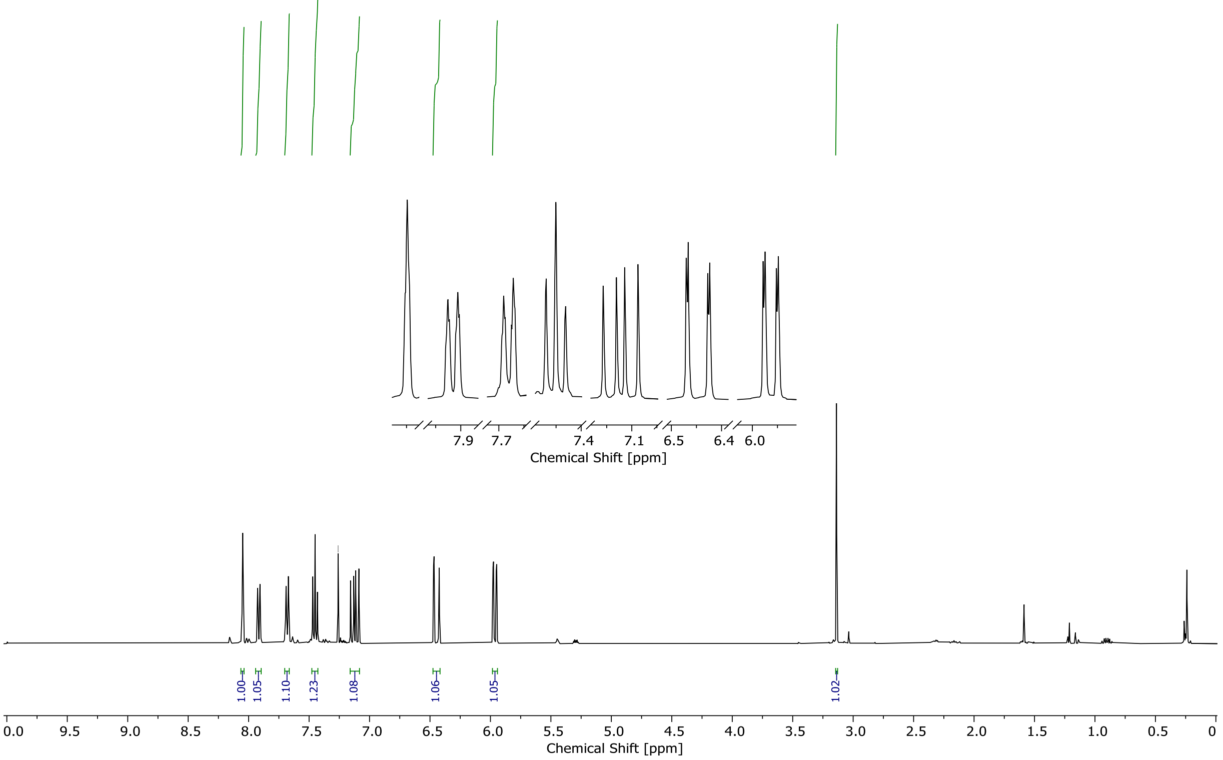

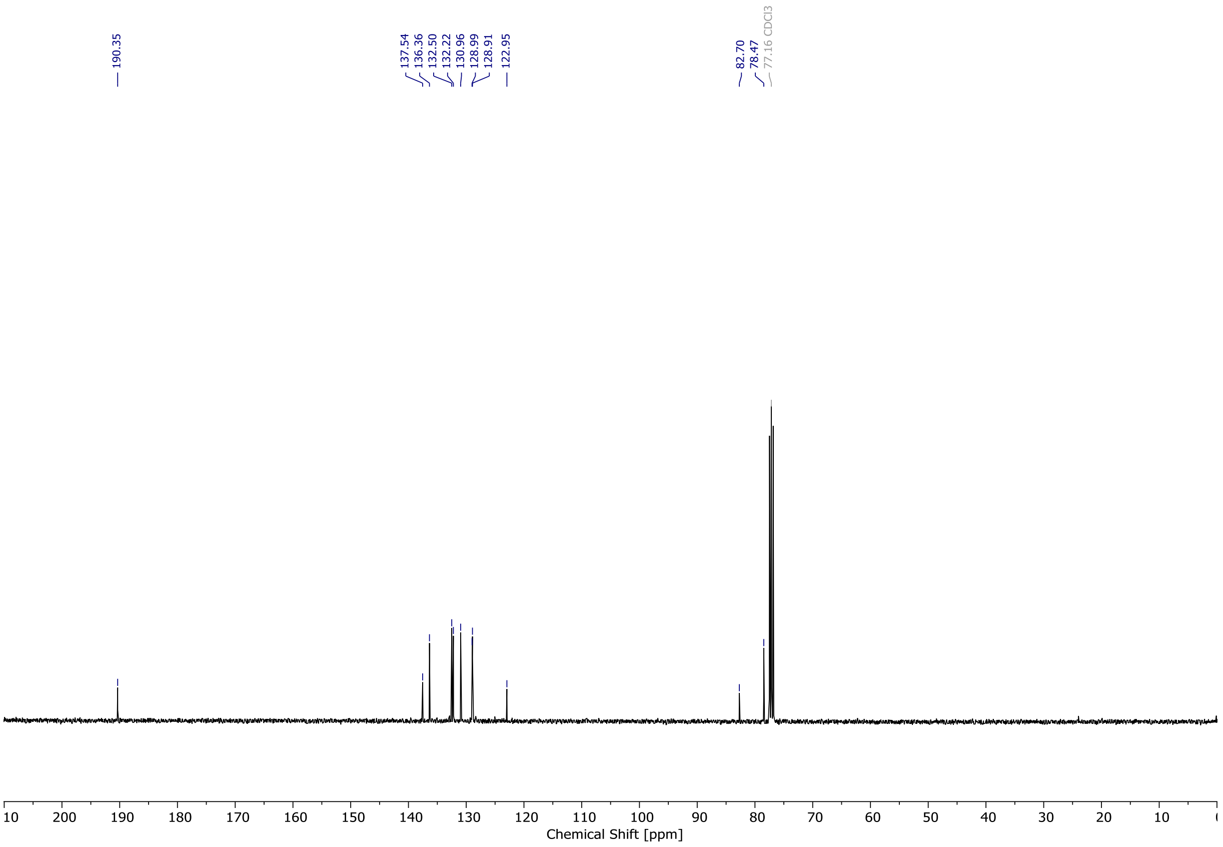

^{1}H NMR (400 MHz, $CDCl_3$) and **^{13}C NMR** (101 MHz, $CDCl_3$)

Methyl *N*-((Benzyloxy)carbonyl)-*S*-(3-(3-ethynylphenyl)-3-oxopropyl)-L-cysteinate (Cys-APA, **13**)

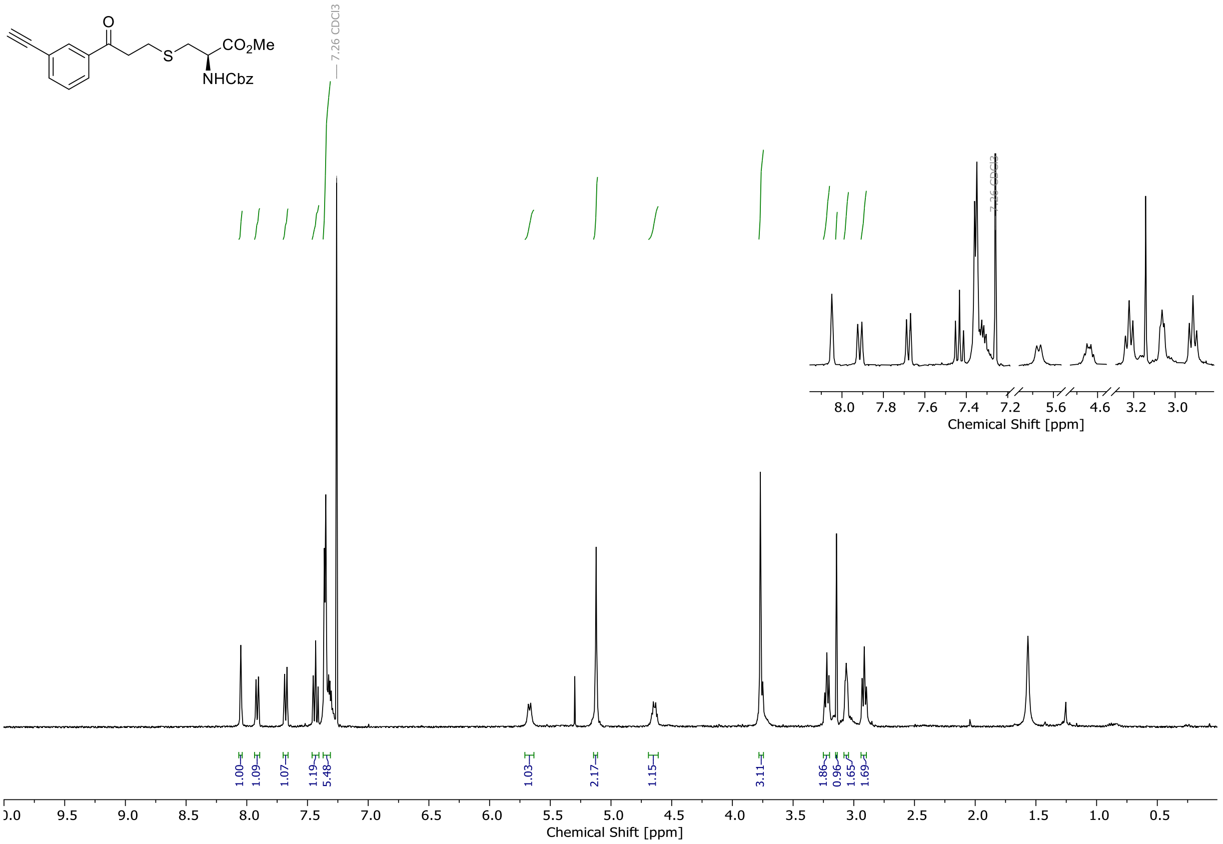

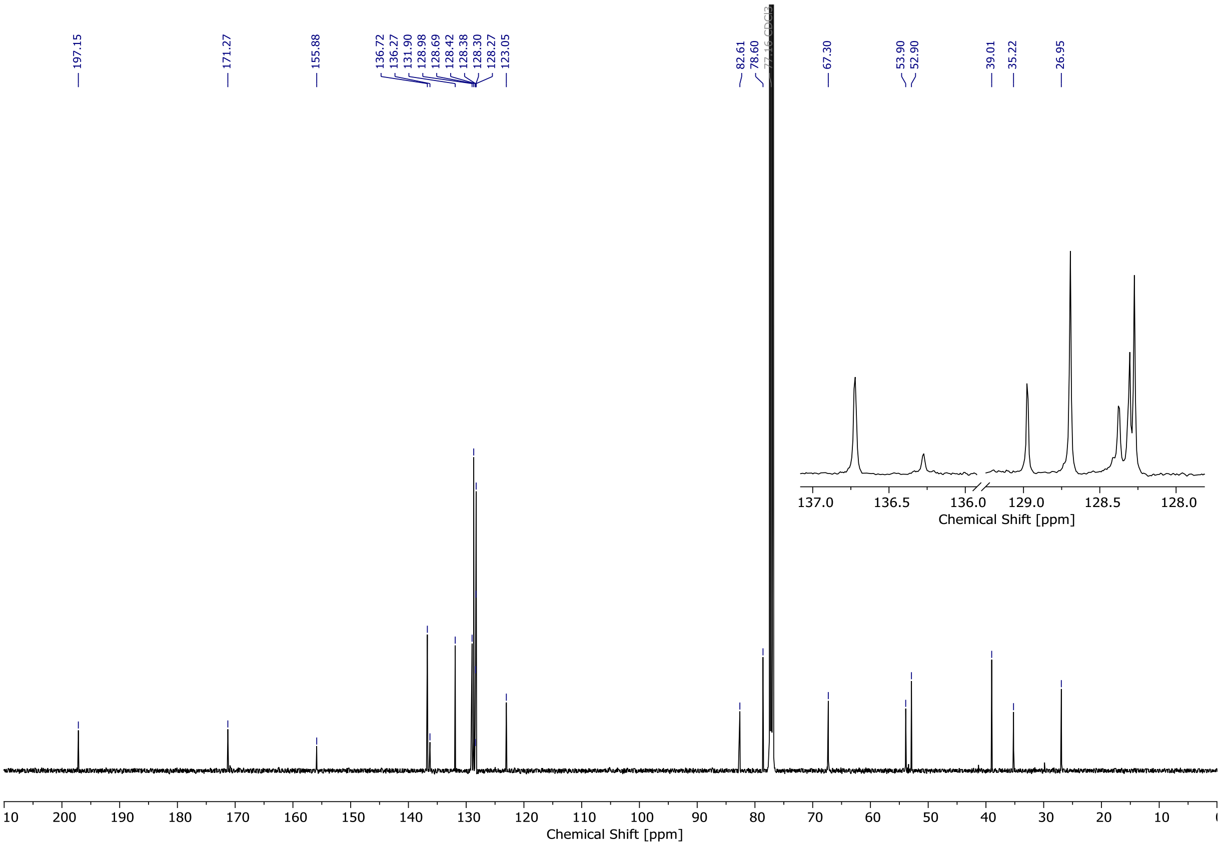

^{1}H NMR (400 MHz, $CDCl_3$) and **^{13}C NMR** (101 MHz, $CDCl_3$)

3-Bromo-2,6-dichlorobenzaldehyde (**S12**)

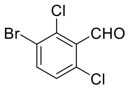

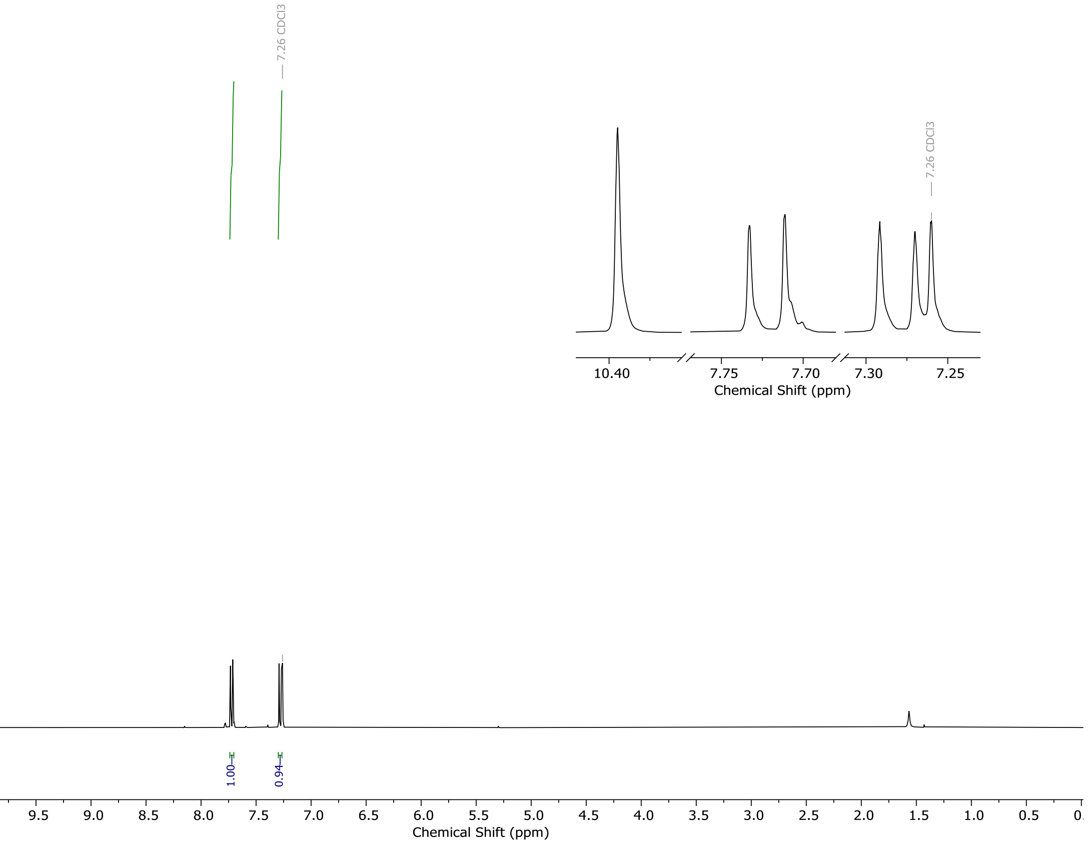

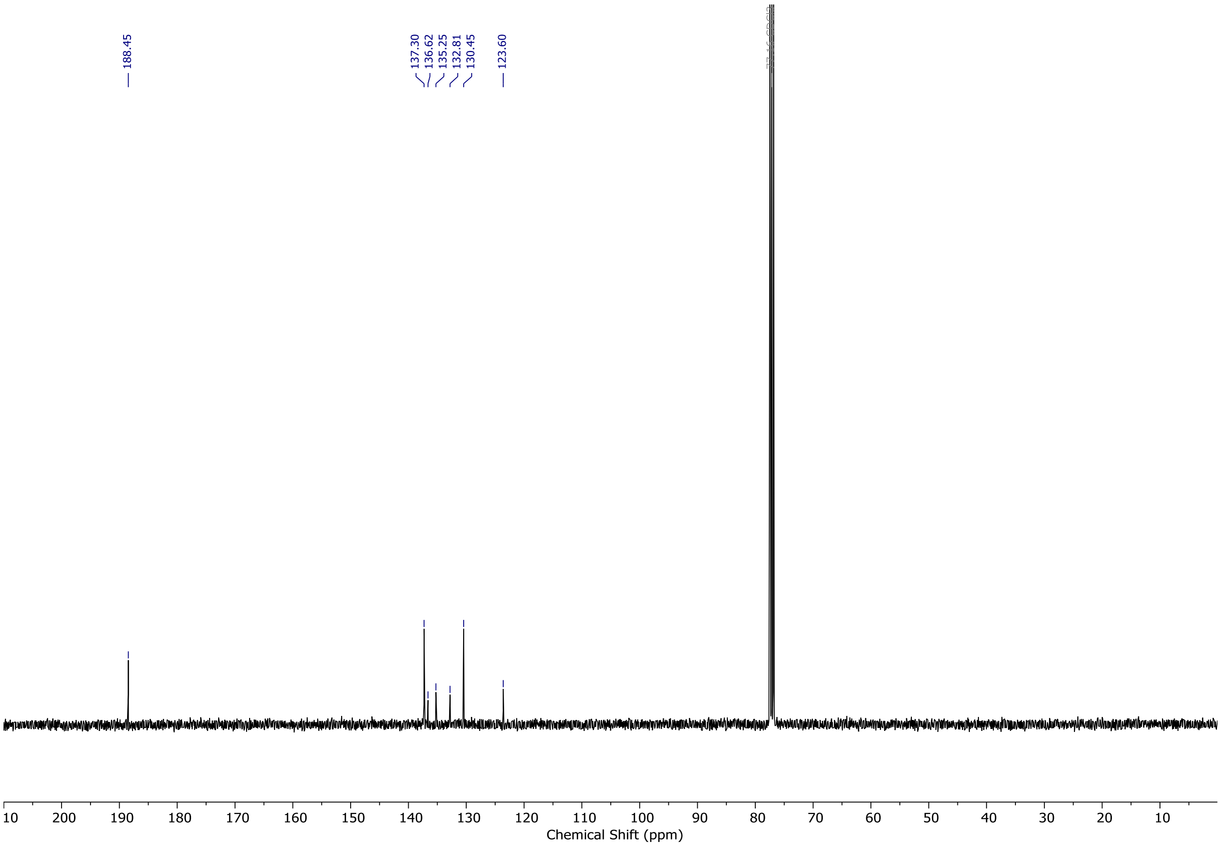

¹H NMR (400 MHz, $CDCl_3$) and **¹³C NMR** (101 MHz, $CDCl_3$)

2,6-Dichloro-3-((trimethylsilyl)ethynyl)benzaldehyde (**S14**)

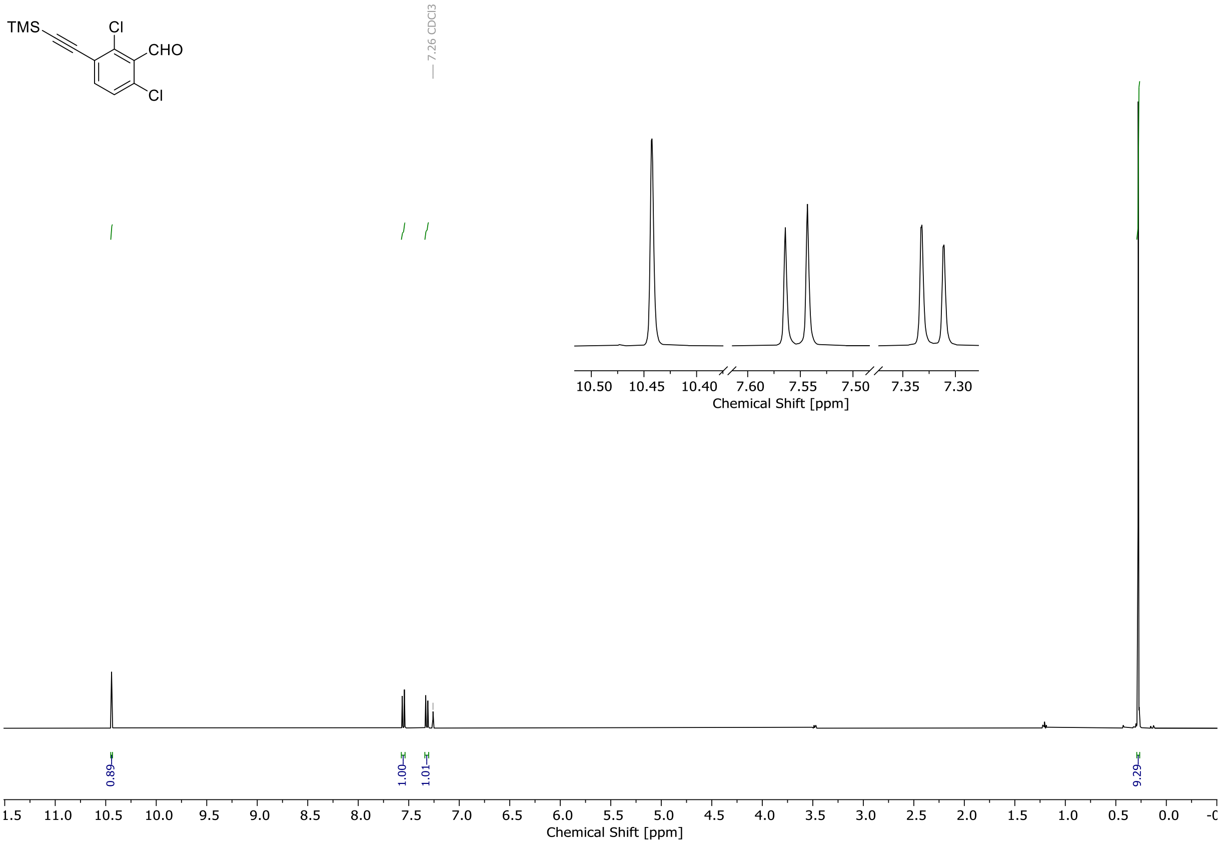

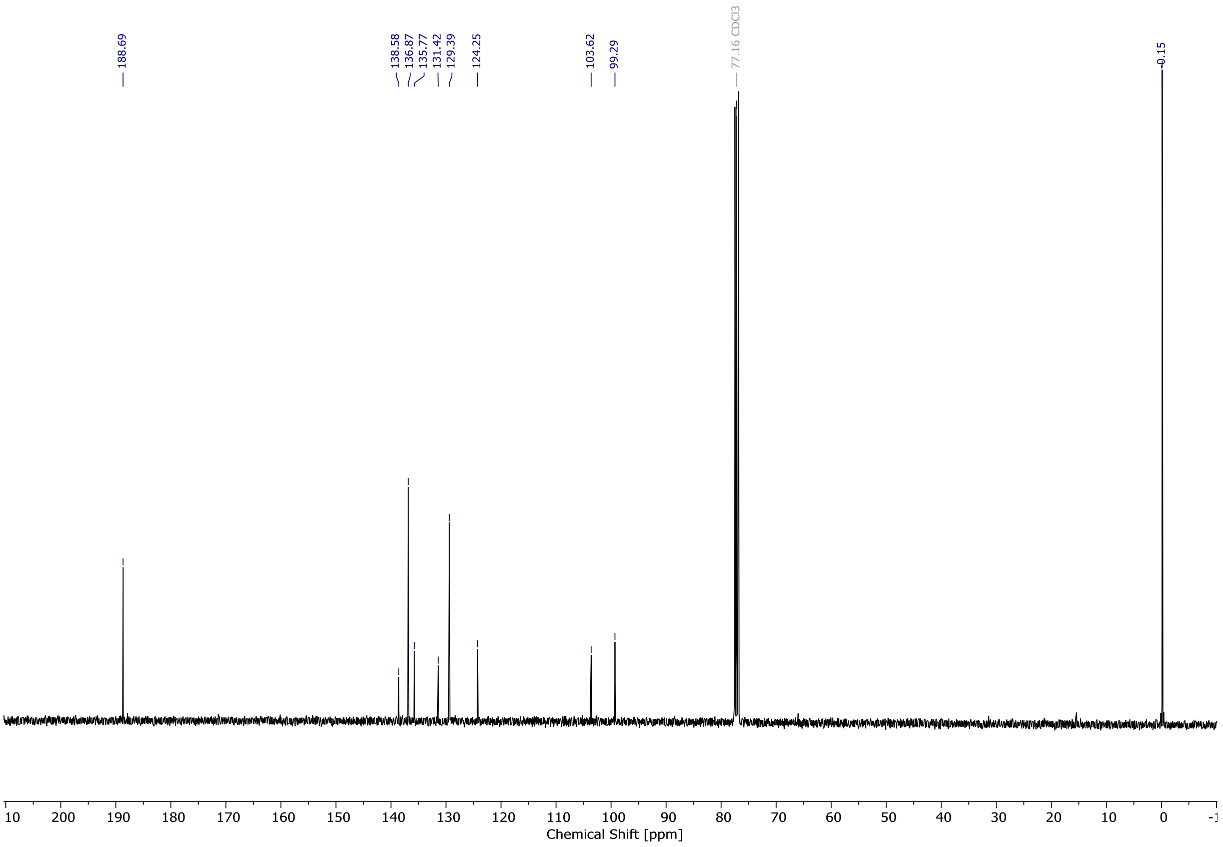

¹H NMR (400 MHz, $CDCl_3$) and **¹³C NMR** (101 MHz, $CDCl_3$)

1-(2,6-Dichloro-3-((trimethylsilyl)ethynyl)phenyl)prop-2-en-1-ol (**S15**)

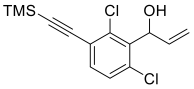

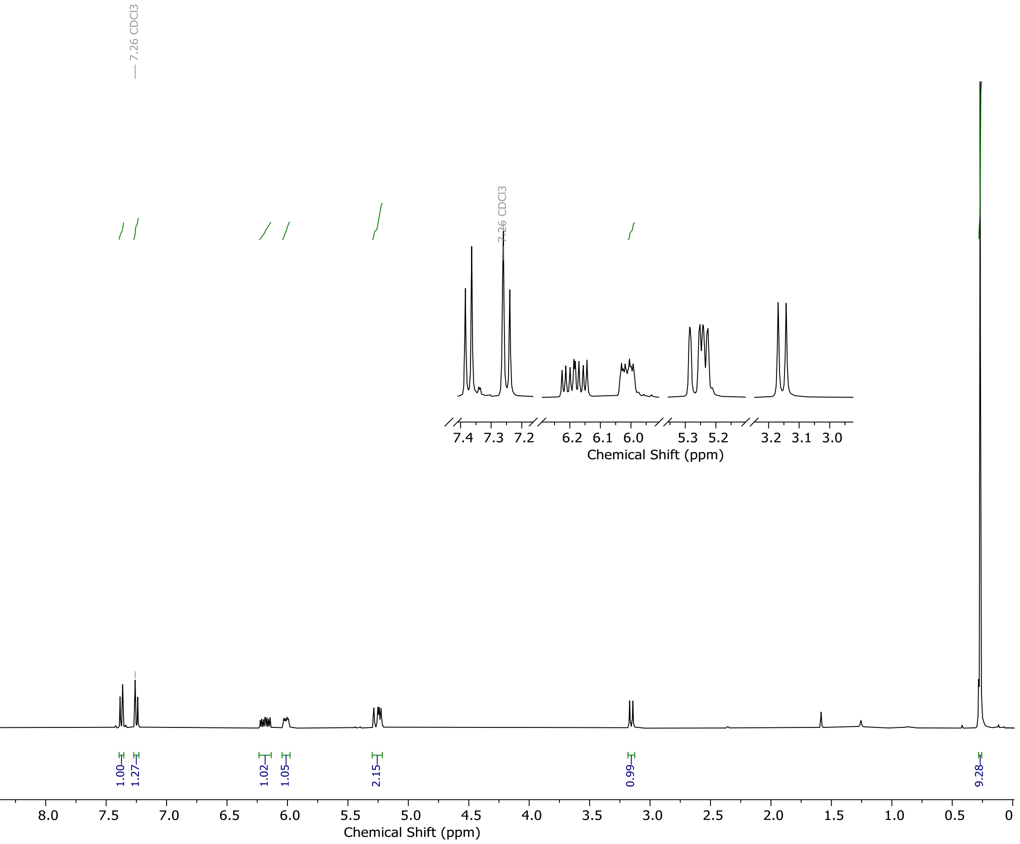

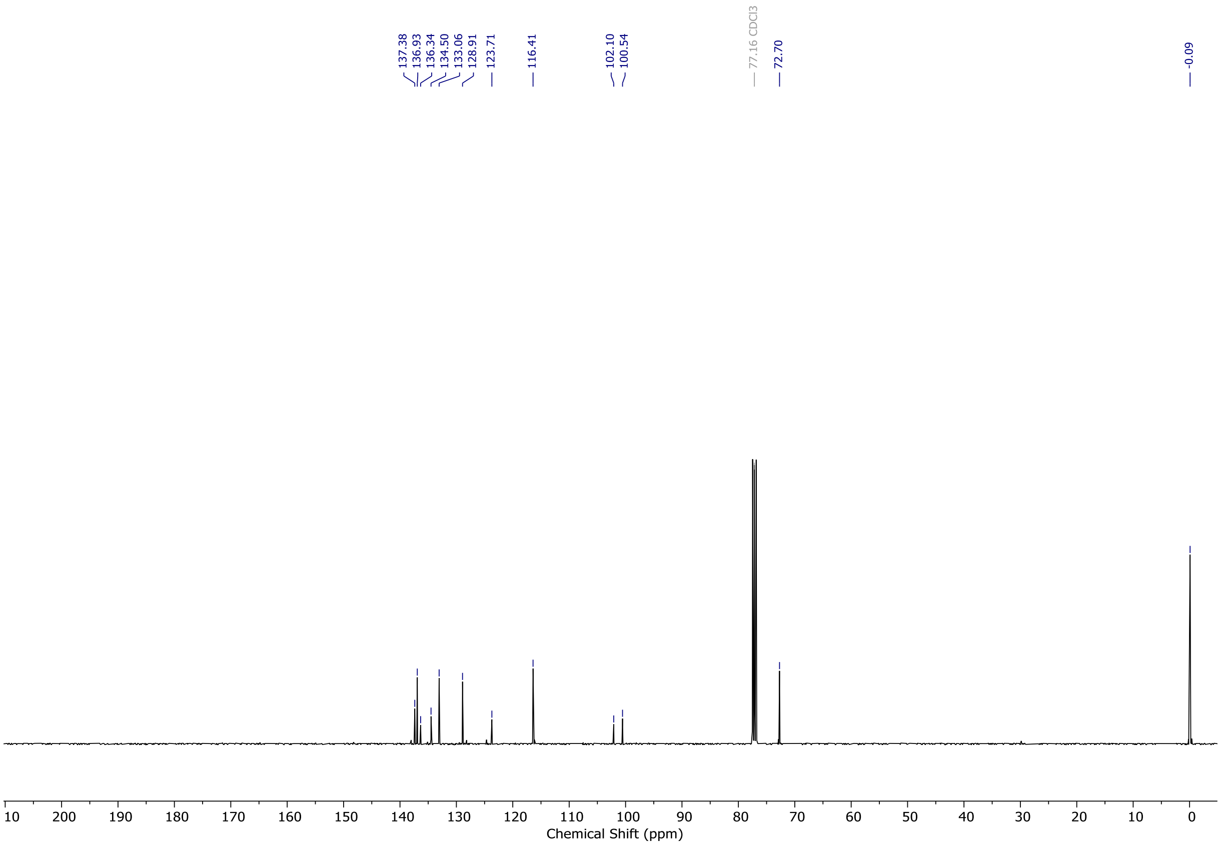

^{1}**H NMR** (400 MHz, $CDCl_3$) and ^{13}**C NMR** (101 MHz, $CDCl_3$)

1-(2,6-Dichloro-3-((trimethylsilyl)ethynyl)phenyl)prop-2-en-1-one (**S16**)

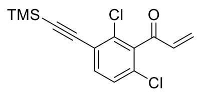

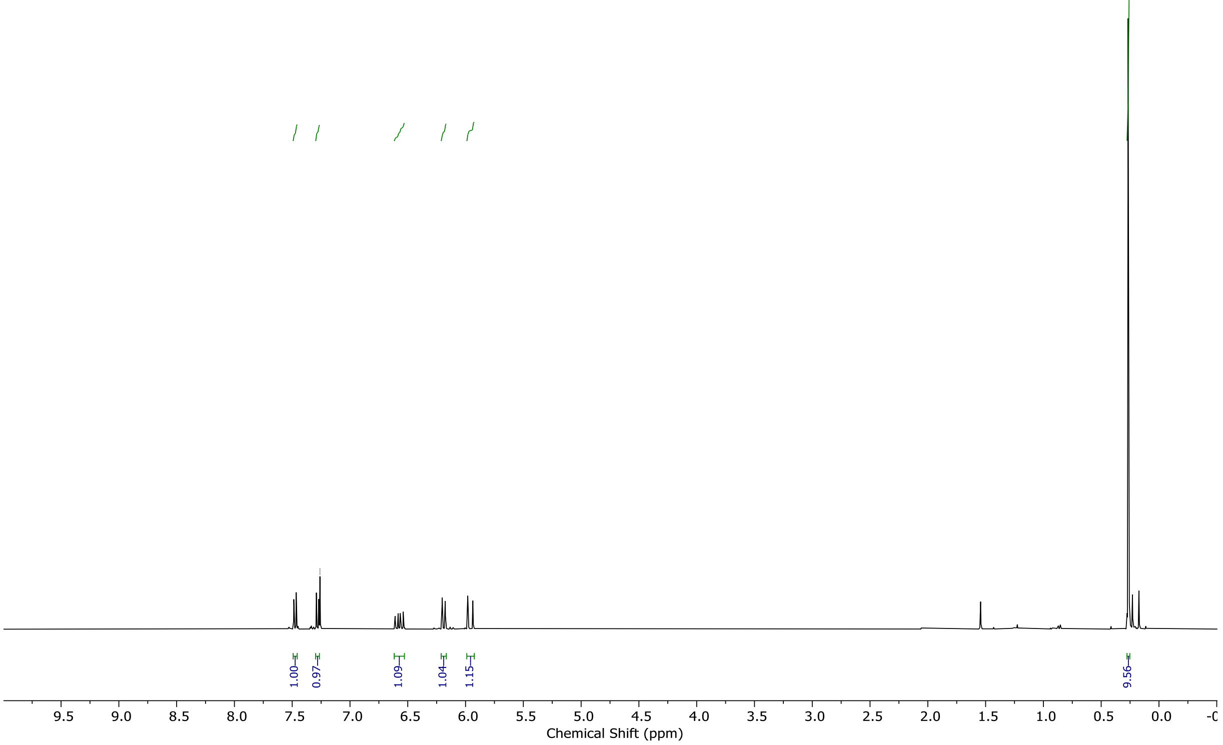

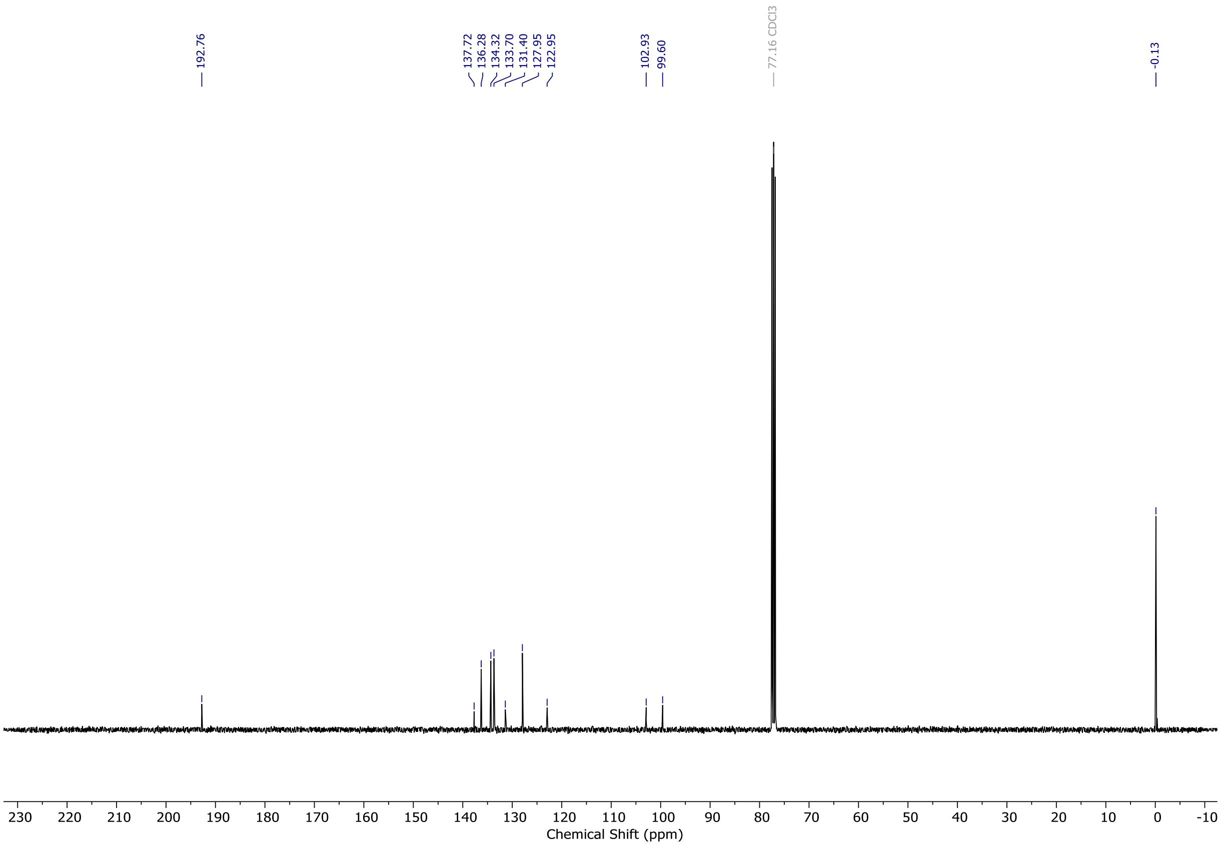

^{1}H NMR (400 MHz, $CDCl_3$) and **^{13}C NMR** (101 MHz, $CDCl_3$)

1-(2,6-Dichloro-3-ethynylphenyl)prop-2-en-1-one (CAPA, **11**)

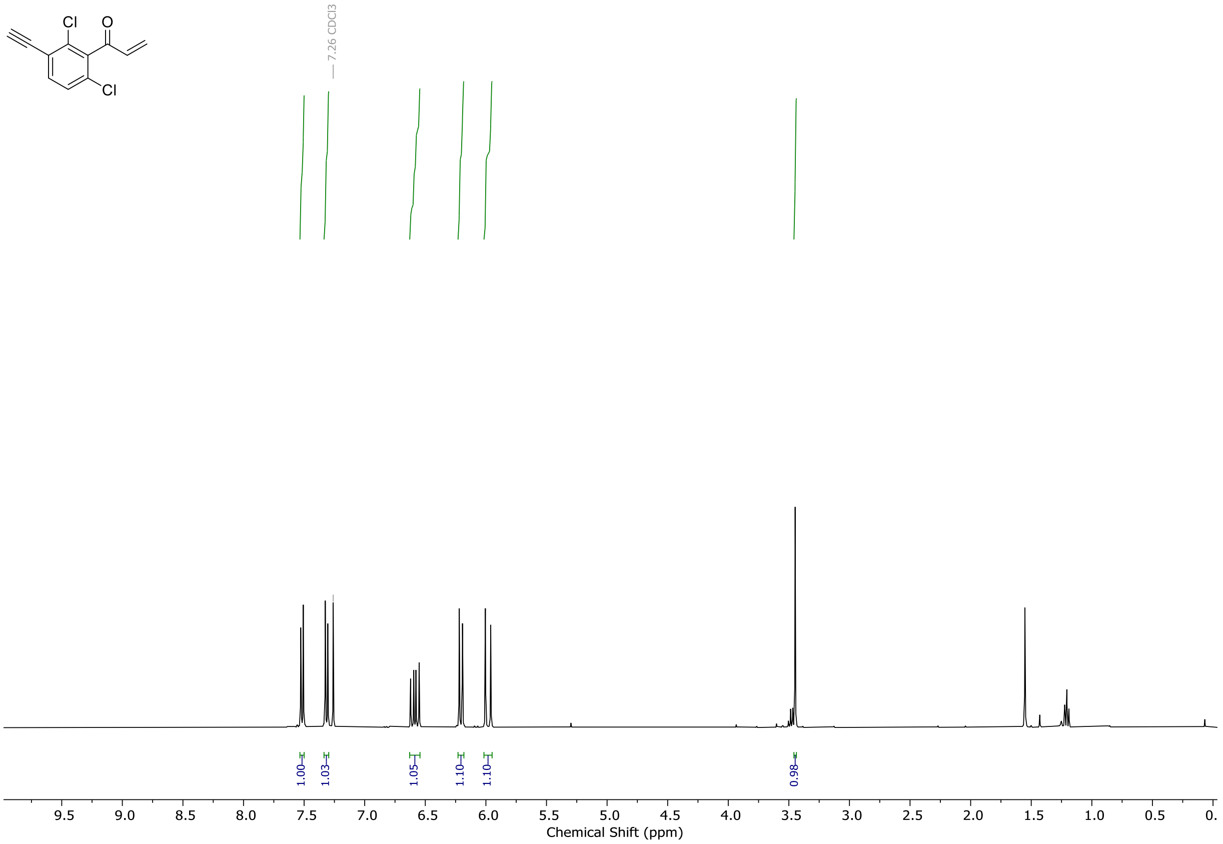

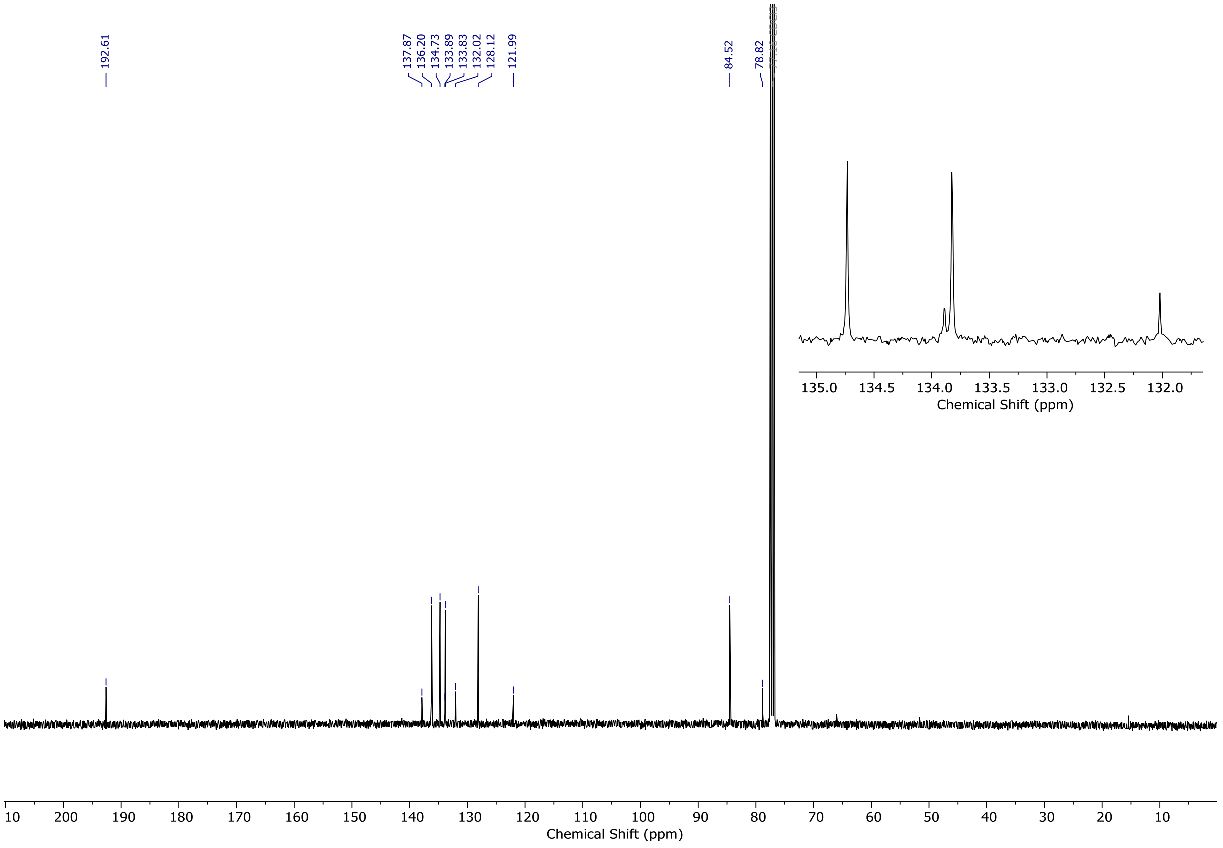

¹H NMR (400 MHz, $CDCl_3$) and **¹³C NMR** (101 MHz, $CDCl_3$)

Methyl *N*-((Benzyloxy)carbonyl)-*S*-(3-(2,6-dichloro-3-ethynylphenyl)-3-oxopropyl)-L-cysteinate (Cys-CAPA, **14**)

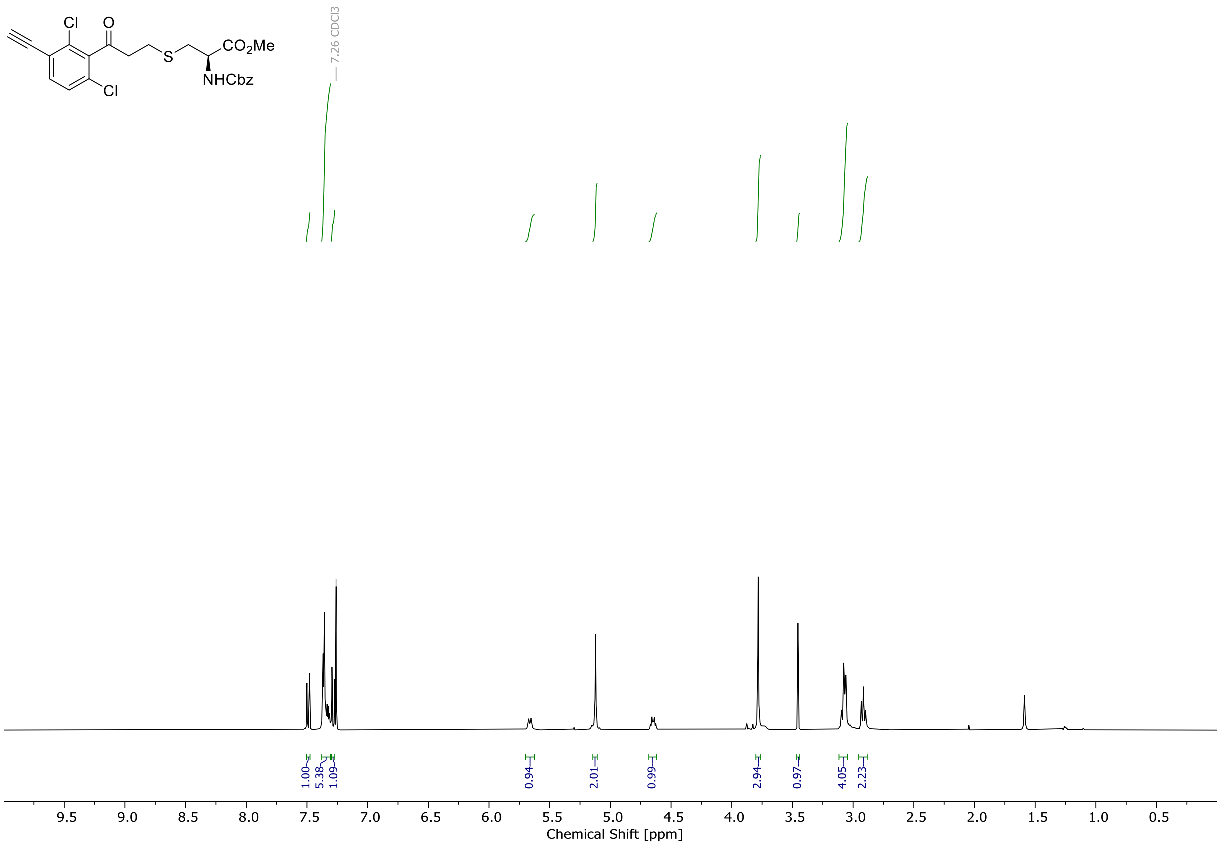

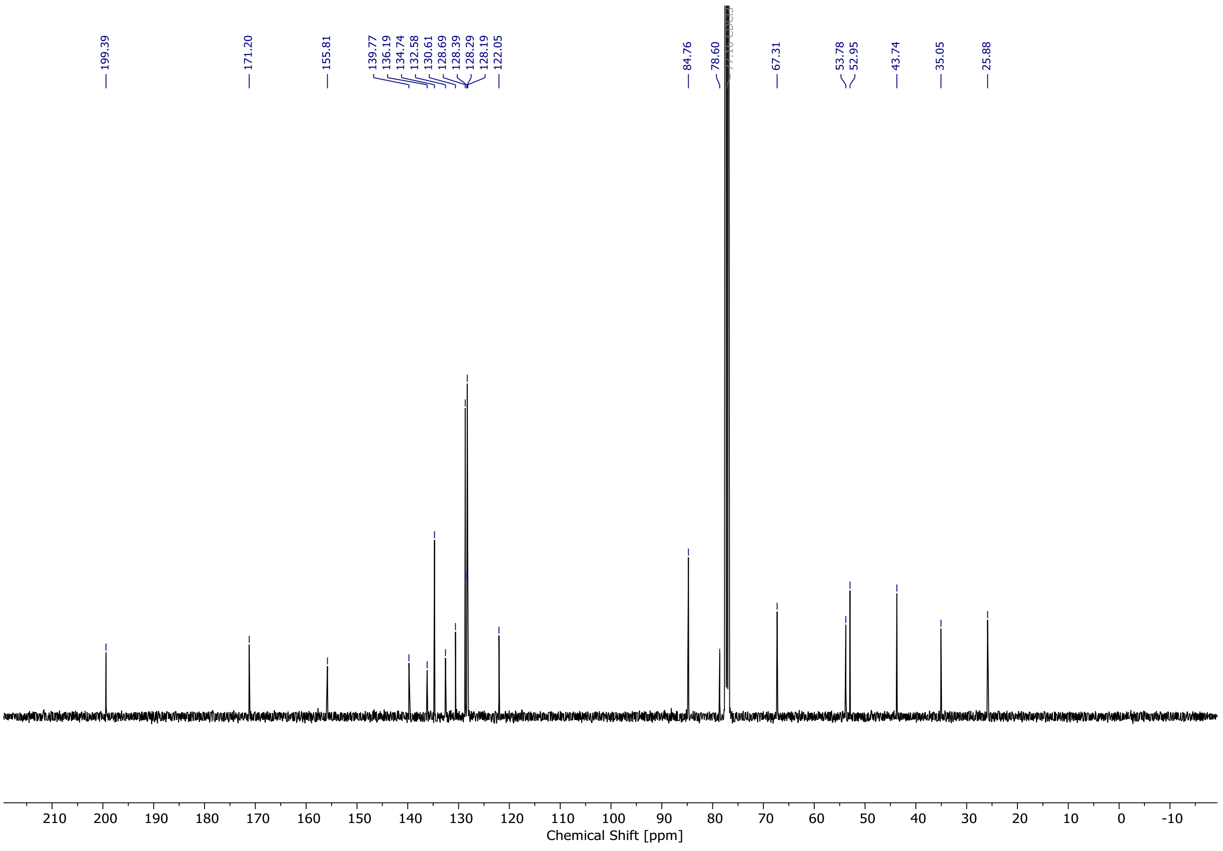

^{1}H NMR (400 MHz, $CDCl_3$) and **^{13}C NMR** (101 MHz, $CDCl_3$)

1-azido-2-(2-(2-(2-azidoethoxy)ethoxy)ethoxy)ethane (N_3-PEG^3-N_3, **S17**)

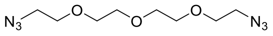

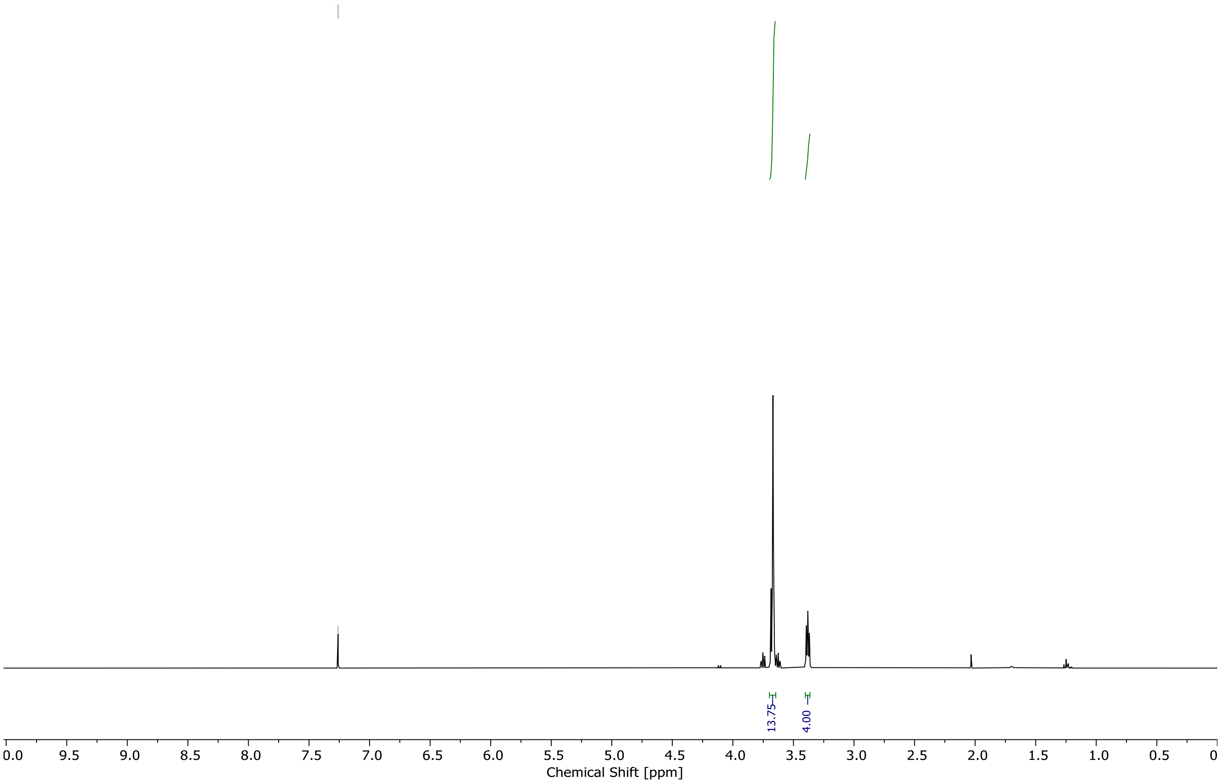

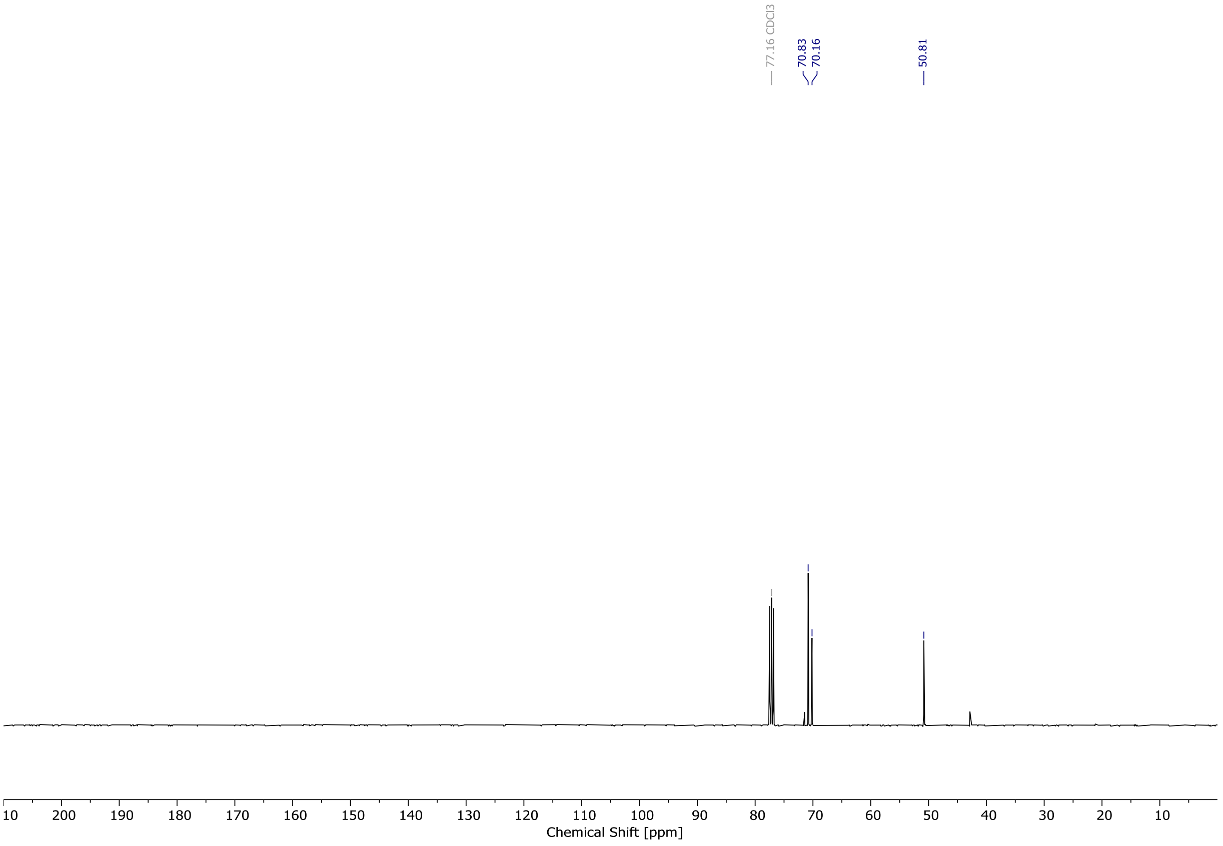

^{1}H NMR (400 MHz, $CDCl_3$) and **^{13}C NMR** (101 MHz, $CDCl_3$)

2-(2-(2-(2-azidoethoxy)ethoxy)ethoxy)ethan-1-amine (N_3-PEG^{3}-NH_2, **S19**)

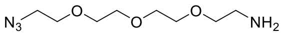

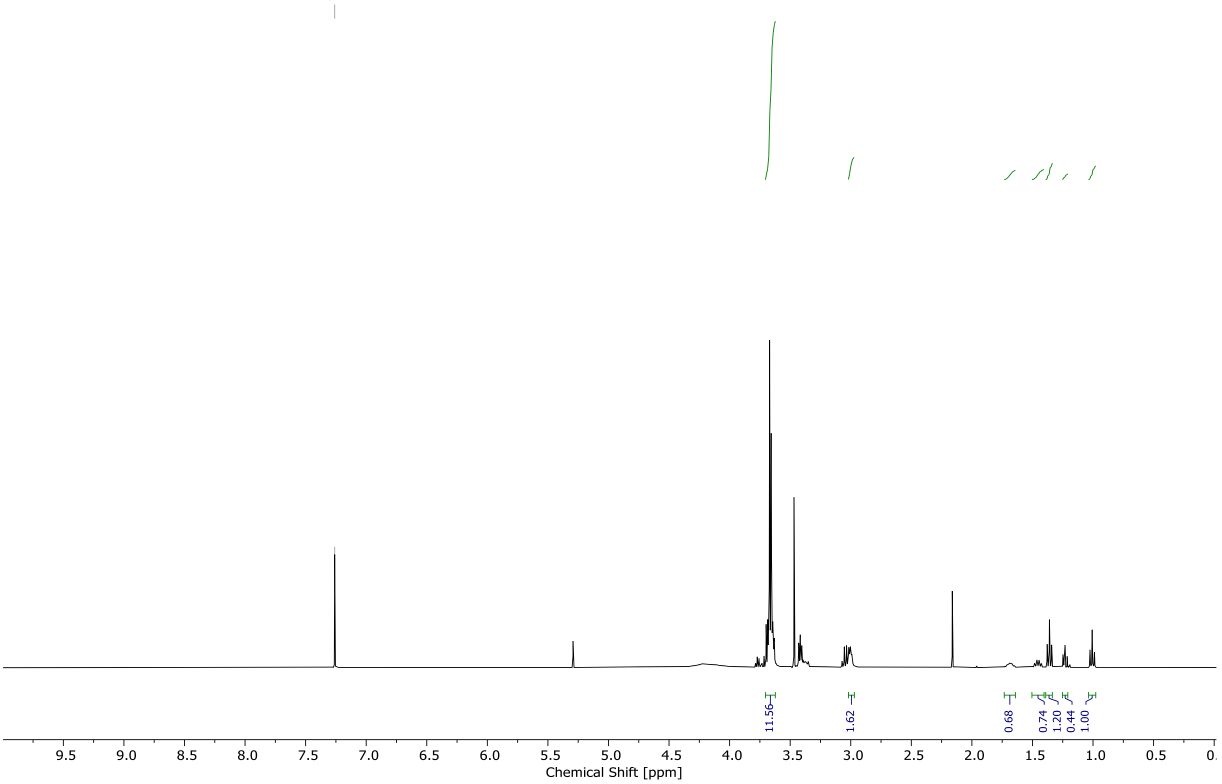

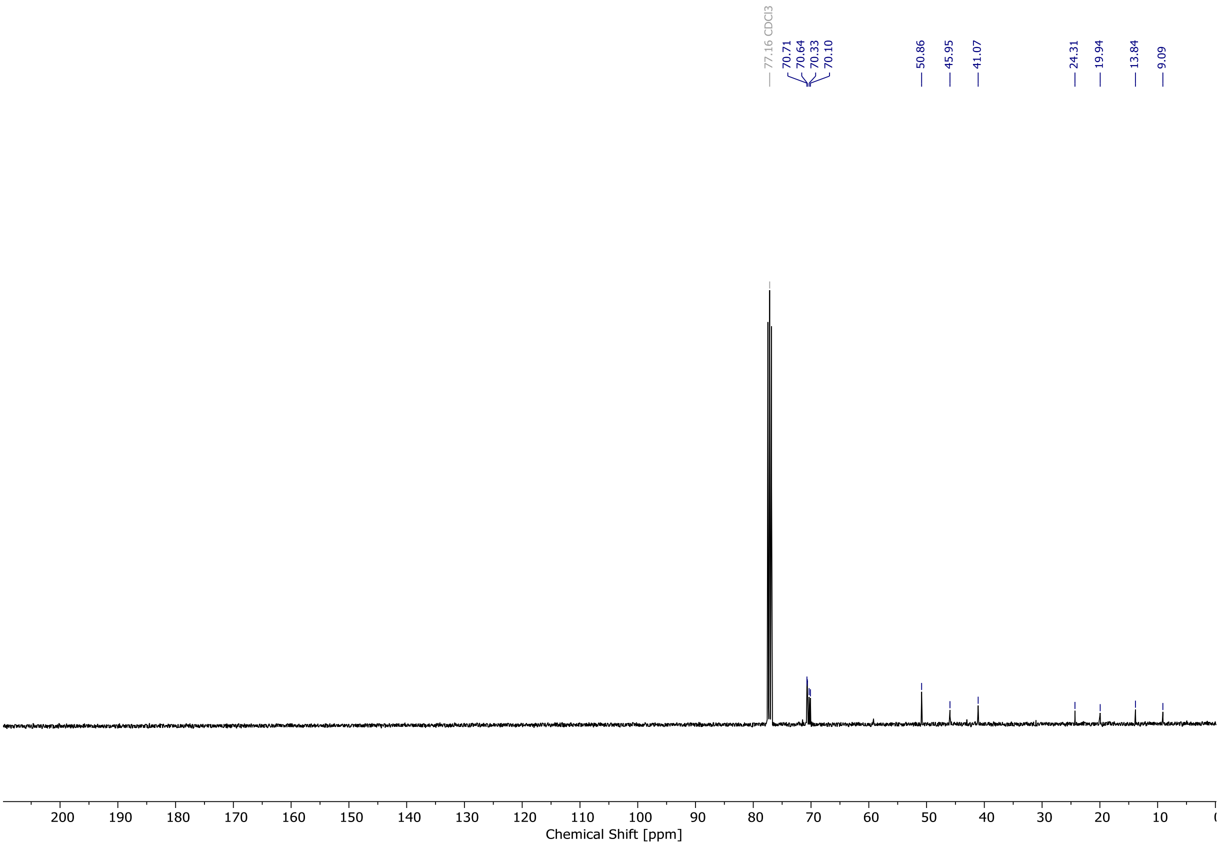

¹H NMR (400 MHz, $(CD_3)_2SO$) and **¹³C NMR** (101 MHz, $(CD_3)_2SO$)

Desthiobiotin (DTB, **S20**)

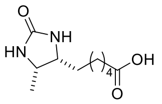

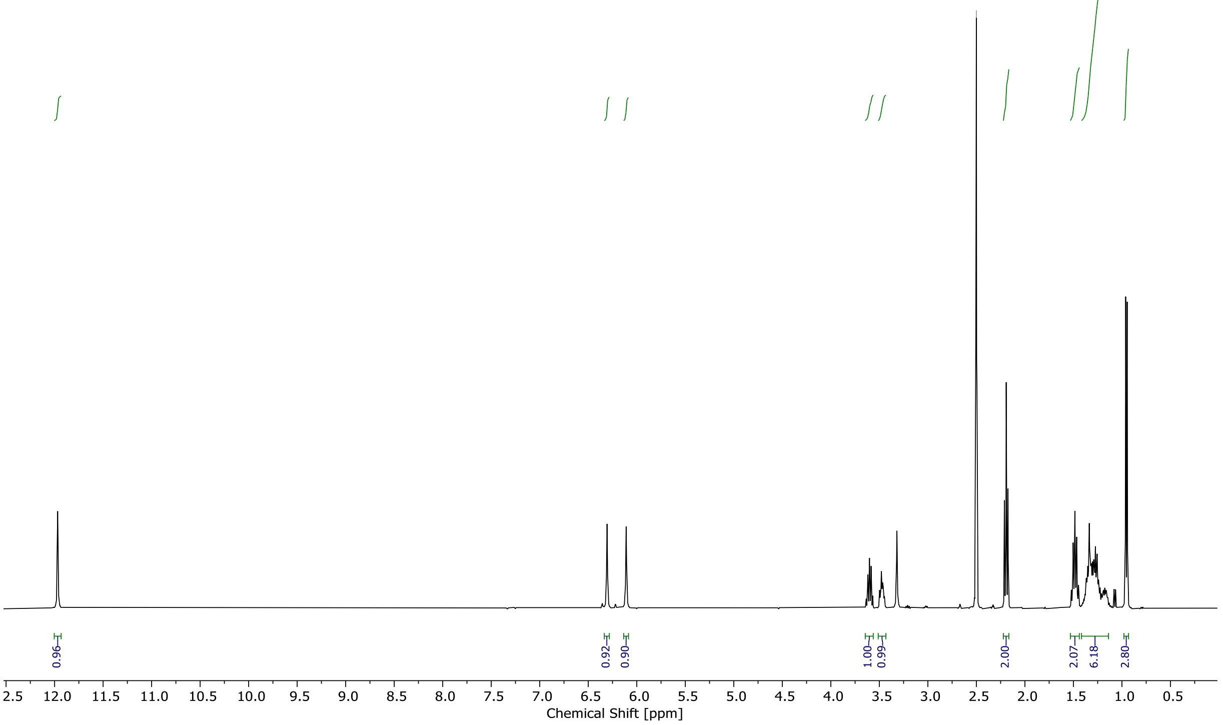

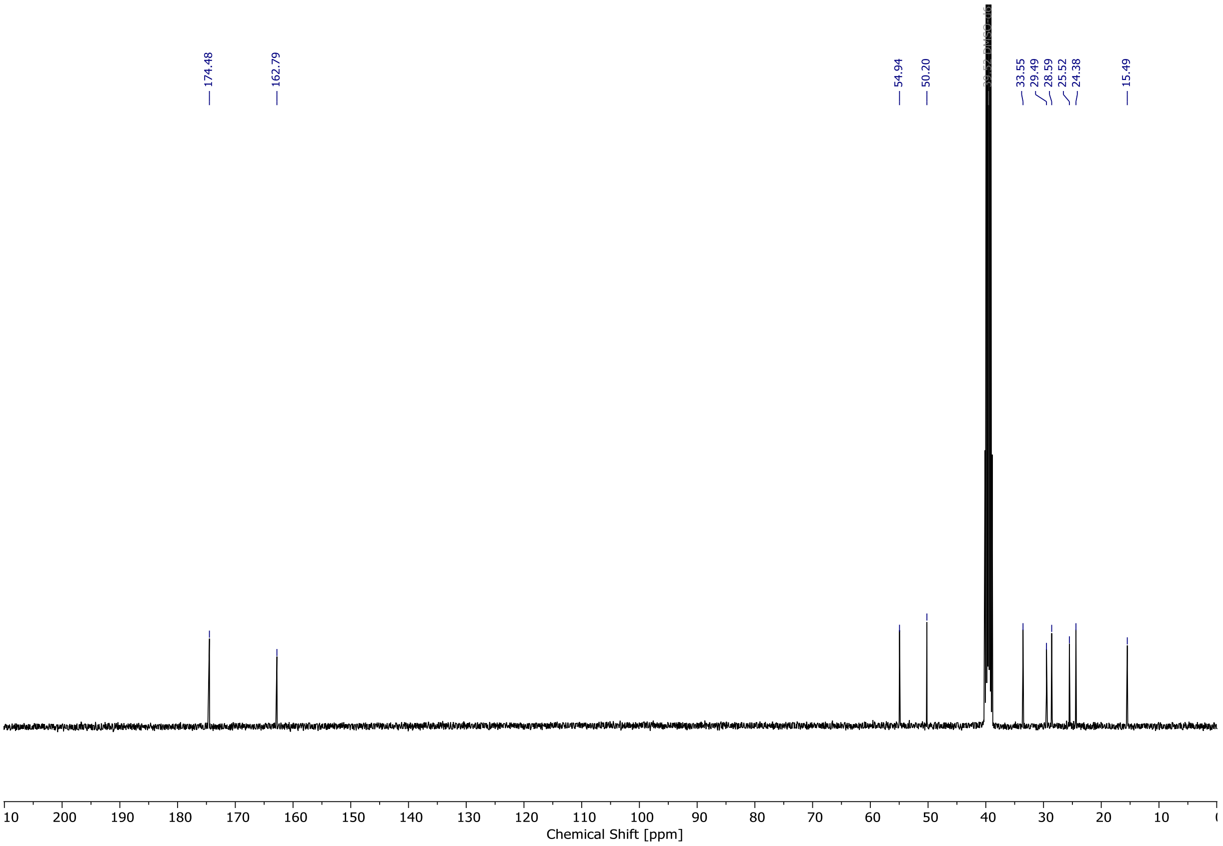

[1]**H NMR** (400 MHz, $CDCl_3$) and [13]**C NMR** (101 MHz, $CDCl_3$)

N-(2-(2-(2-(2-Azidoethoxy)ethoxy)ethoxy)ethyl)-6-((4*R*,5*S*)-5-methyl-2-oxoimidazolidin-4-yl)hexanamide (DTB-PEG[3]-N_3, **16**)

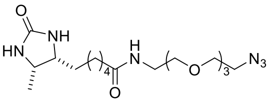

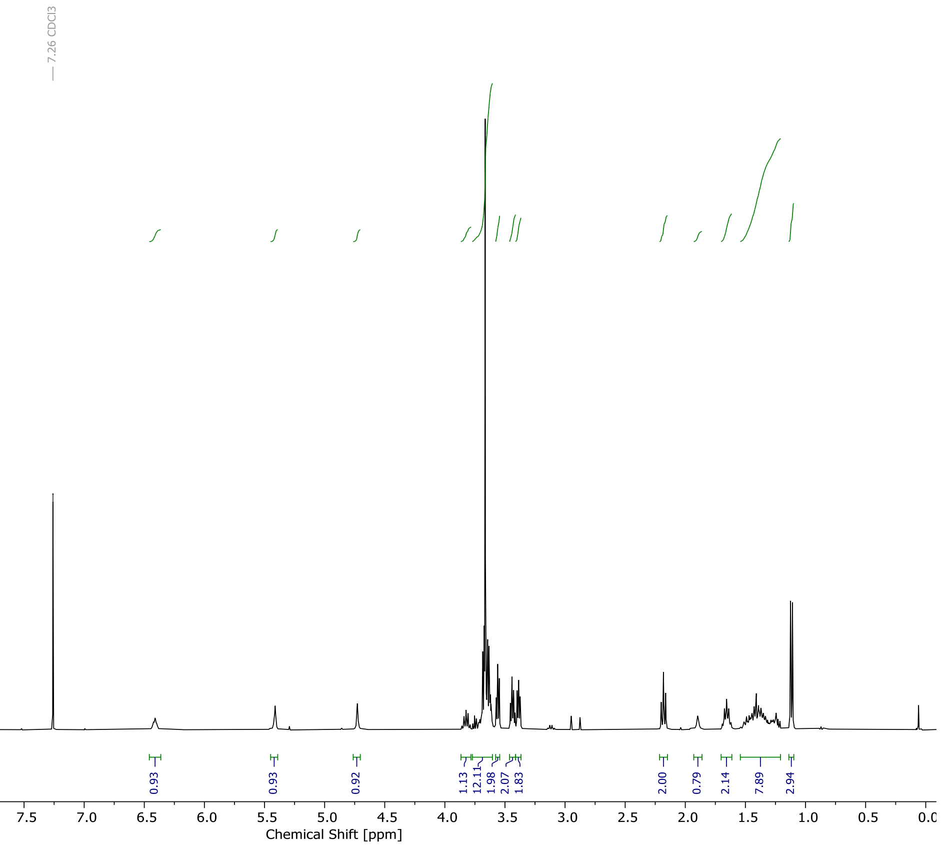

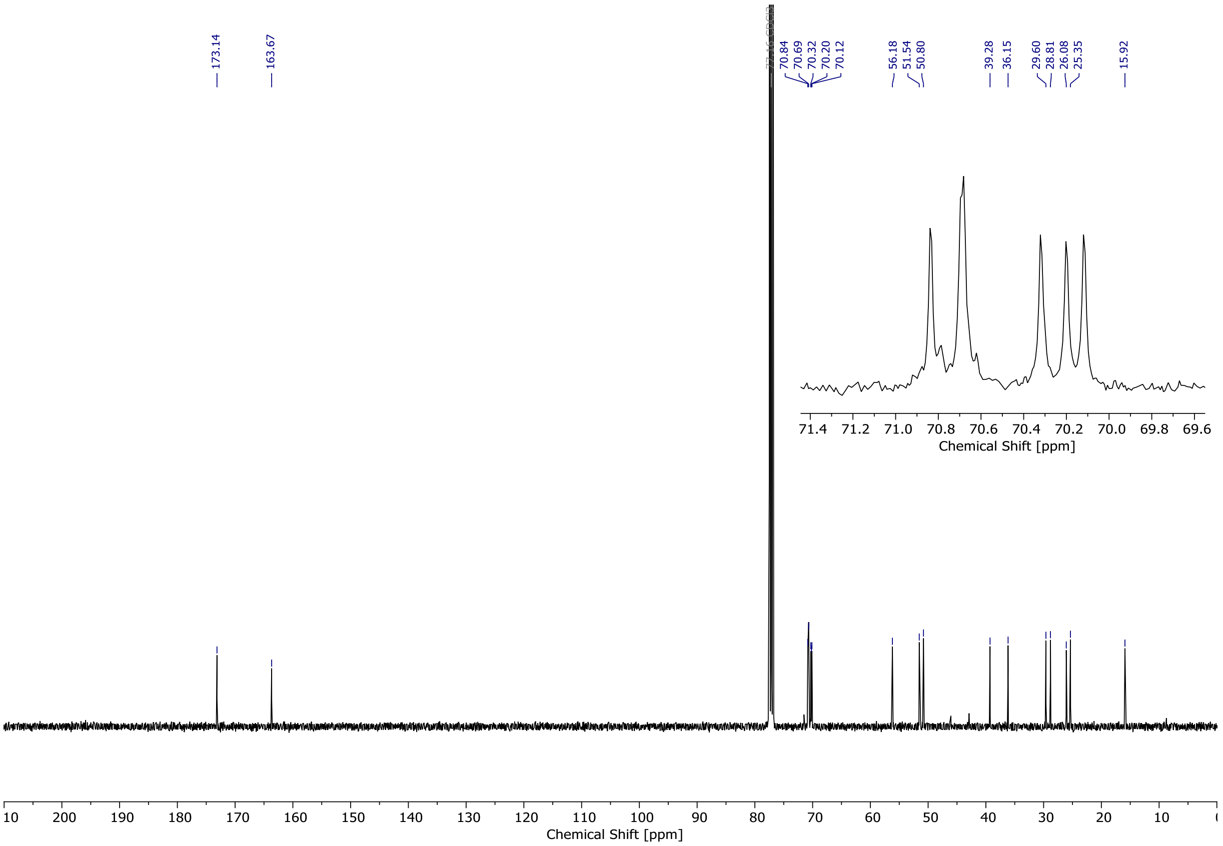

^{1}H NMR (400 MHz, $CDCl_3$), **^{13}C NMR** (101 MHz, $CDCl_3$) and corresponding **COSY**, **HMQC**, **HMBC**

Methyl *N*-((Benzyloxy)carbonyl)-*S*-(3-(2,6-dichloro-3-(1-(18-((4*R*,5*S*)-5-methyl-2-oxoimidazolidin-4-yl)-13-oxo-3,6,9-trioxa-12-azaoctadecyl)-1*H*-1,2,3-triazol-4-yl)phenyl)-3-oxopropyl)-L-cysteinate (Cys-CAPA-DTB, **15**)

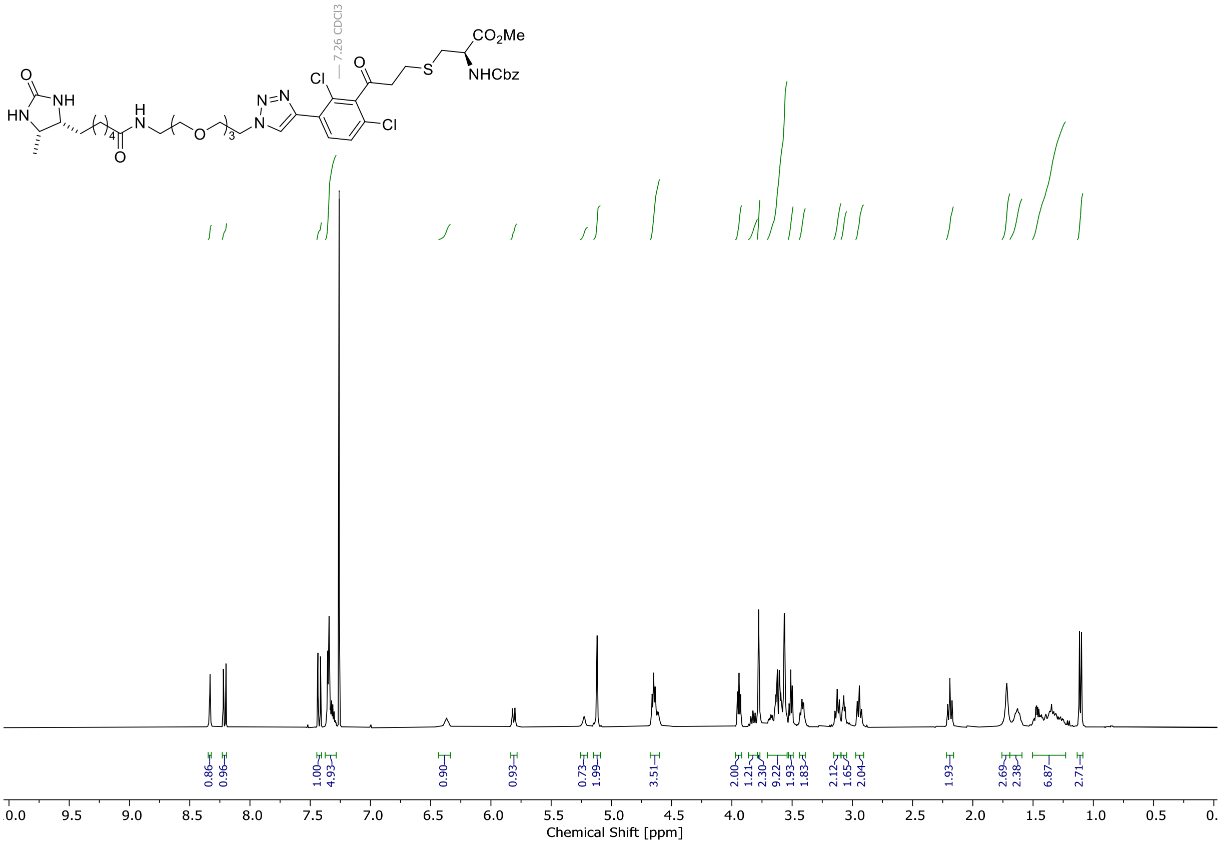

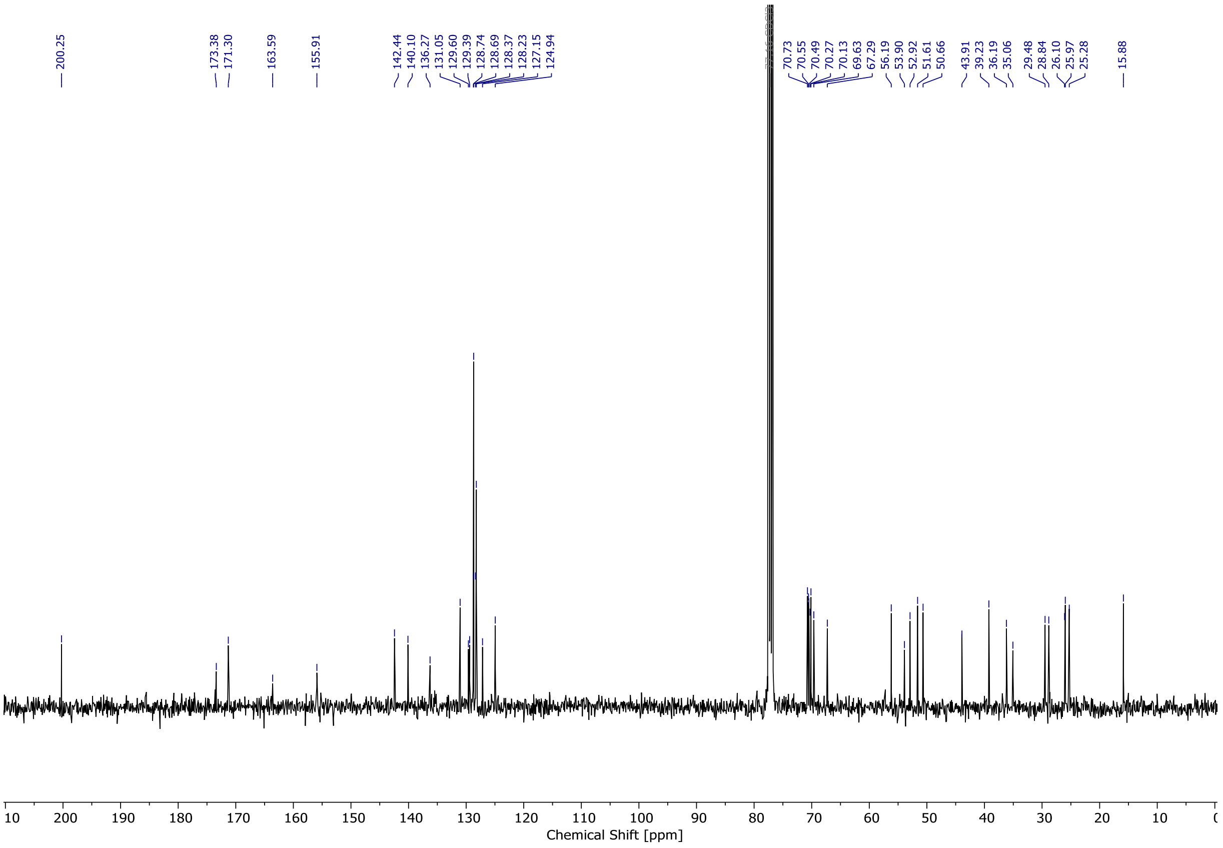

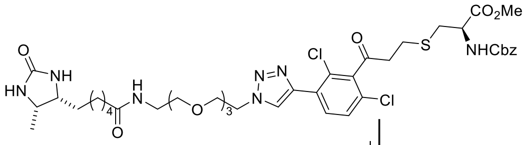

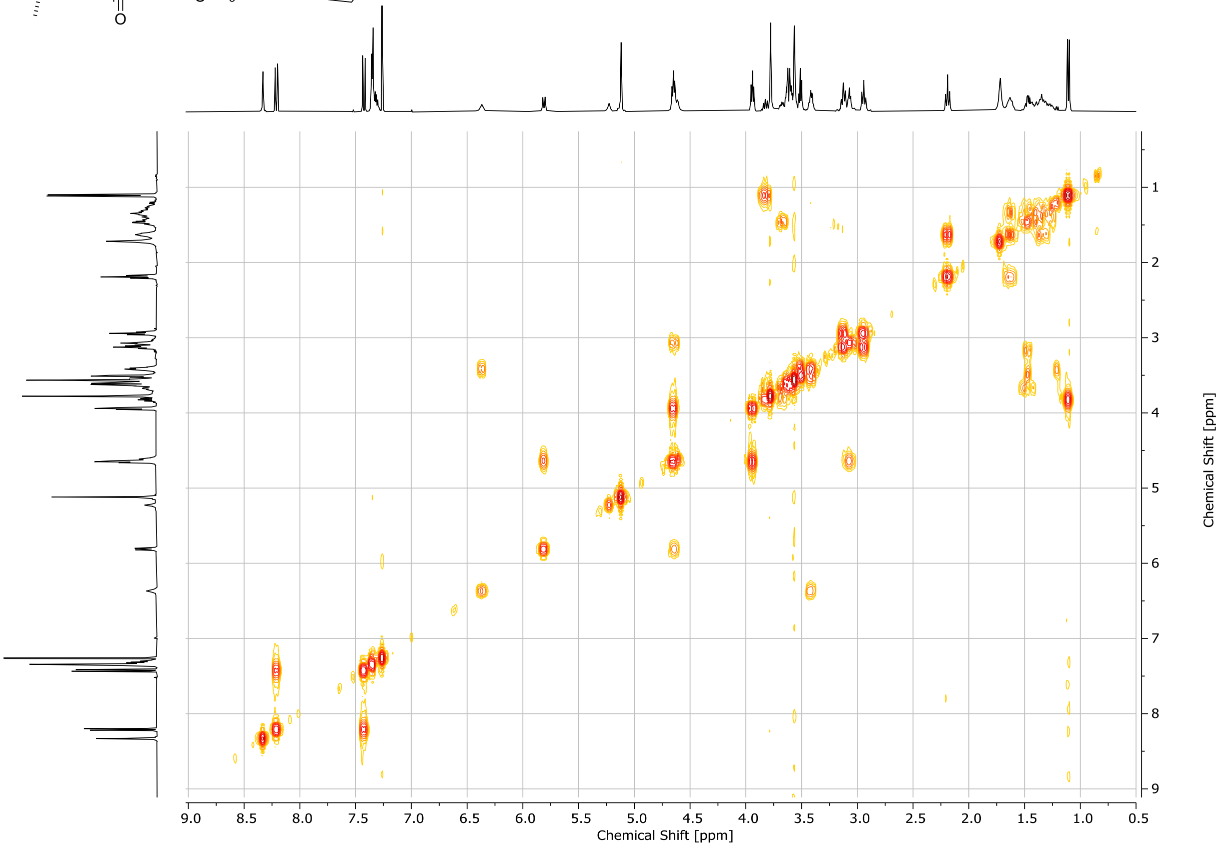
Chemical Shift [ppm]
Chemical Shift [ppm]

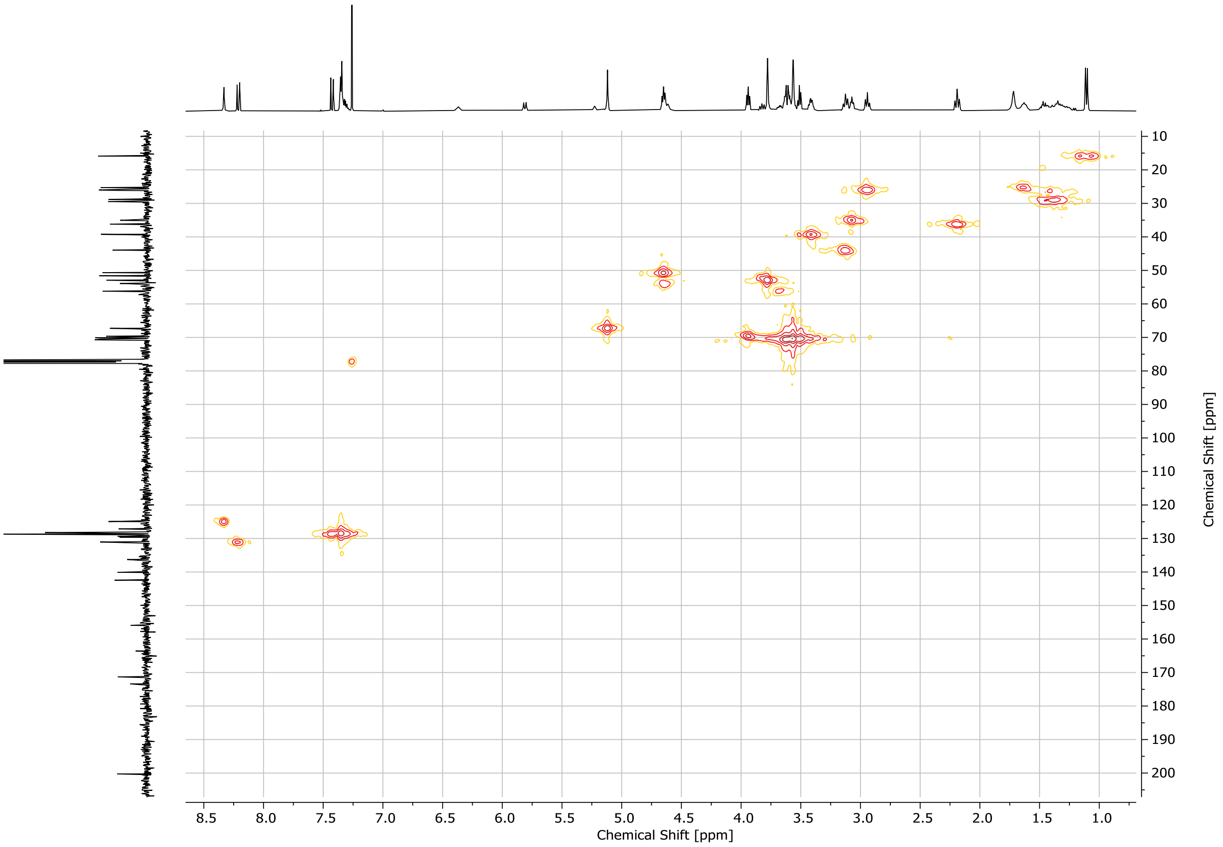
Chemical Shift [ppm]
Chemical Shift [ppm]

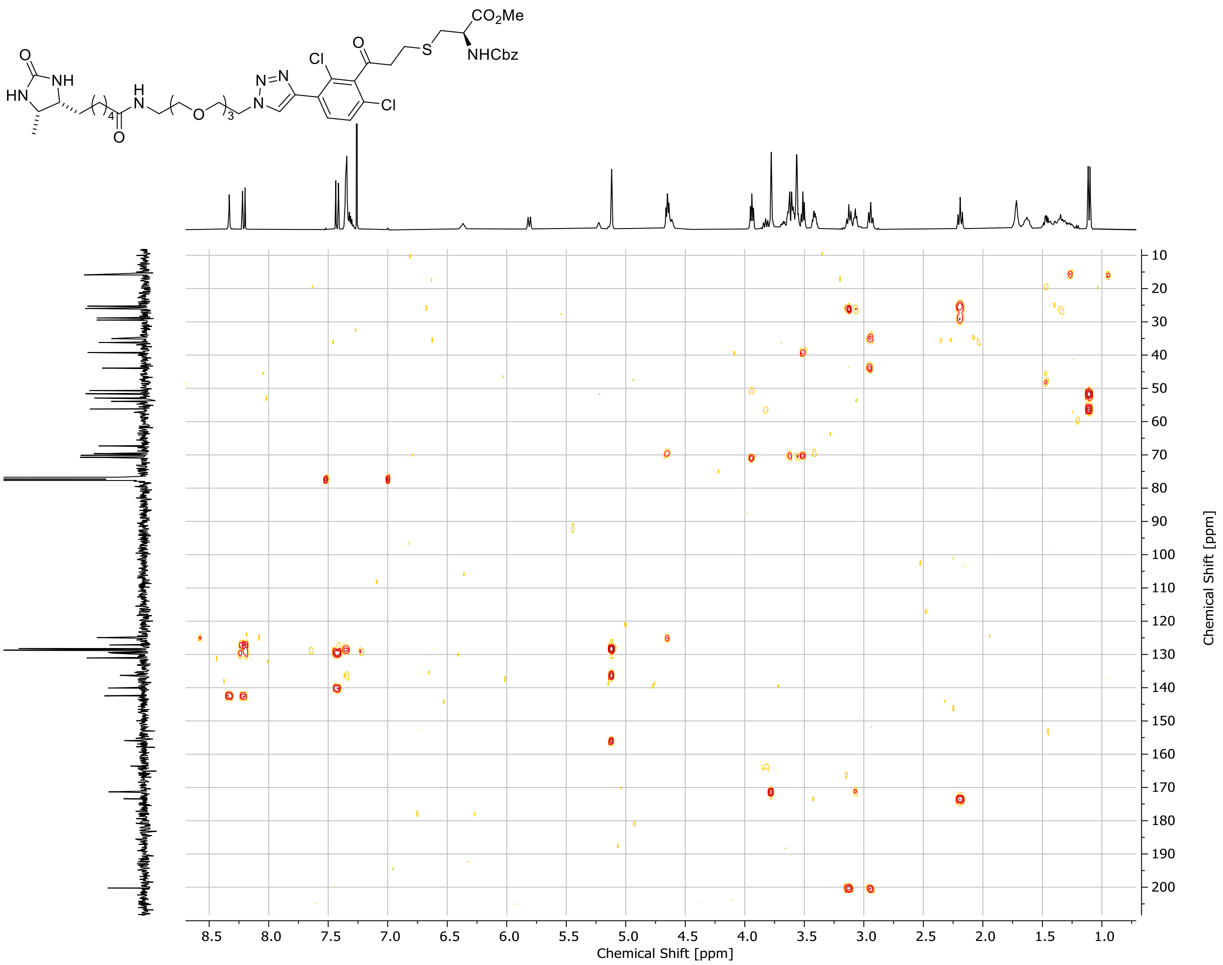
CO2Me
NHCbz
Cl
O
S
N=N
N
Cl
O
NH
HN
H
N
O
4
3
O
Chemical Shift [ppm]
Chemical Shift [ppm]

9. References:

(1) Zhang, X.; Liang, N.; Li, R.; Sun, Z. Application of Halogen-Bonding Catalysis for Markovnikov-Type Hydrothiolation of Alkenes. *Synlett* **2023**, *34* (04), 379–387. https://doi.org/10.1055/a-1984-9105.

(2) Ananda, K.; Suresh Babu, V. V. Deprotonation of Hydrochloride Salts of Amino Acid Esters and Peptide Esters Using Commercial Zinc Dust. *The Journal of Peptide Research* **2001**, *57* (3), 223–226. https://doi.org/10.1111/j.1399-3011.2001.00790.x.

(3) Thuong, M. B. T.; Mann, A.; Wagner, A. Mild Chemo-Selective Hydration of Terminal Alkynes Catalysed by $AgSbF_6$. *Chem. Commun.* **2012**, *48* (3), 434–436. https://doi.org/10.1039/C1CC12928G.

(4) Robles, O.; Serna-Saldívar, S. O.; Gutiérrez-Uribe, J. A.; Romo, D. Cyclopropanations of Olefin-Containing Natural Products for Simultaneous Arming and Structure Activity Studies. *Org. Lett.* **2012**, *14* (6), 1394–1397. https://doi.org/10.1021/ol300105q.

(5) Zanon, P. R. A.; Lewald, L.; Hacker, S. M. Isotopically Labeled Desthiobiotin Azide (isoDTB) Tags Enable Global Profiling of the Bacterial Cysteinome. *Angewandte Chemie* **2020**, *132* (7), 2851–2858. https://doi.org/10.1002/ange.201912075.

(6) Robke, L.; Rodrigues, T.; Schröder, P.; Foley, D. J.; Bernardes, G. J. L.; Laraia, L.; Waldmann, H. Discovery of 2,4-Dimethoxypyridines as Novel Autophagy Inhibitors. *Tetrahedron* **2018**, *74* (35), 4531–4537. https://doi.org/10.1016/j.tet.2018.07.021.

(7) Brauch, S.; Henze, M.; Osswald, B.; Naumann, K.; Wessjohann, L. A.; Van Berkel, S. S.; Westermann, B. Fast and Efficient MCR-Based Synthesis of Clickable Rhodamine Tags for Protein Profiling. *Org. Biomol. Chem.* **2012**, *10* (5), 958–965. https://doi.org/10.1039/C1OB06581E.

(8) Mangubat-Medina, A. E.; Martin, S. C.; Hanaya, K.; Ball, Z. T. A Vinylogous Photocleavage Strategy Allows Direct Photocaging of Backbone Amide Structure. *J. Am. Chem. Soc.* **2018**, *140* (27), 8401–8404. https://doi.org/10.1021/jacs.8b04893.

(9) LaVallie, E. R.; DiBlasio, E. A.; Kovacic, S.; Grant, K. L.; Schendel, P. F.; McCoy, J. M. A Thioredoxin Gene Fusion Expression System That Circumvents Inclusion Body Formation in the E. Coli Cytoplasm. *Nat Biotechnol* **1993**, *11* (2), 187–193. https://doi.org/10.1038/nbt0293-187.

(10) Chen, H.; Wu, D.; Holzinger, J.; Götz, R.; Didier, D.; Schütz, A. K.; Schneider, S.; Kielkowski, P. Aryl Radicals Generated from Aryl Pinacol Boronates Modify Peptides and Proteins. *Eur J Org Chem* **2025**, *28* (7), e202401246. https://doi.org/10.1002/ejoc.202401246.

(11) Frankenfield, A. M.; Ni, J.; Ahmed, M.; Hao, L. Protein Contaminants Matter: Building Universal Protein Contaminant Libraries for DDA and DIA Proteomics. *J. Proteome Res.* **2022**, *21* (9), 2104–2113. https://doi.org/10.1021/acs.jproteome.2c00145.
